# A Metallosupramolecular Receptor for Squaraine Dyes Enabling Ultrafast Dark Resonance Energy Transfer

**DOI:** 10.1002/anie.2203782

**Published:** 2026-02-01

**Authors:** Damien W. Chen, Tejas Deshpande, Sybille Collignon, Farzaneh Fadaei‐Tirani, Sascha Feldmann, Kay Severin

**Affiliations:** ^1^ Institut des Sciences et Ingénierie Chimiques Ecole Polytechnique Fédérale de Lausanne (EPFL) Lausanne Switzerland

**Keywords:** cage, fluorescence, receptor, squaraine dye, supramolecular chemistry

## Abstract

A metal‐organic cage was obtained by combining acridone‐based dipyridyl ligands with Pd^2+^ ions. The cage acts as a potent receptor for squaraine dyes, with a pronounced preference for guests with 2,6‐dihydroxyphenyl substituents. This selectivity profile differs from that of previously reported receptors for squaraine dyes. The acridone‐based cage itself is non‐emissive. Upon its photoexcitation, ultrafast (sub‐ps) dark resonance energy transfer (DRET) to the encapsulated squaraine dyes was observed, resulting in bright, near‐infrared guest emission, with pseudo‐Stokes shifts of up to 440 nm. Upon binding of a chiral dye, chirality transfer to the host could be evidenced by circular dichroism spectroscopy.

## Graphical abstract

A metallosupramolecular receptor binds squaraine dyes with high affinity and enables efficient dark resonance energy transfer (DRET) that generates bright near‐infrared emission with large pseudo‐Stokes shifts.

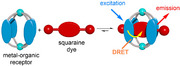

## 1 Introduction

**FIGURE 1:**
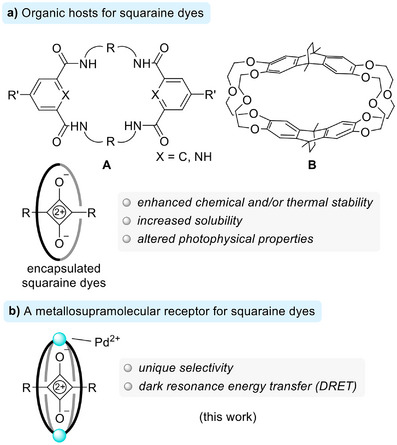
The encapsulation of squaraine dyes by organic hosts can be used to change the chemical and physical properties of the dyes (a). Herein, we report a metallosupramolecular receptor for squaraine dyes that enables dark resonance energy transfer from the host to the dye (b).

The encapsulation of squaraine dyes within organic hosts can significantly modify their chemical and (photo)physical properties [1, 2, 3, 4, 5, 6, 7, 8, 9, 10, 11]. The most commonly employed hosts are tetralactam macrocycles of type **A** (Figure 1a). In 2005, Smith and co‐workers reported in a seminal publication the synthesis of rotaxanes based on tetralactams as ring systems and a squaraine dye as the thread [1]. Encapsulation by the macrocycles was shown to enhance the chemical stability of the dye and suppress the aggregation‐induced broadening of its absorption spectrum. Since then, tetralactams of type **A** have been used extensively for the construction of rotaxanes and pseudo‐rotaxanes with squaraine dyes [2, 3, 4, 5, 6, 7, 8, 9, 10, 11].

The encapsulated dyes display altered photophysical properties. Typically, absorption and emission wavelengths are slightly red‐shifted. The quantum yields can either increase or decrease, depending on the system [1, 2, 3, 4, 5, 6, 7, 8, 9, 10, 11]. In addition to an improved chemical stability, squaraine (pseudo)rotaxanes can display markedly enhanced thermal stability [4]. Different applications have been explored, including near‐infrared fluorescent bioimaging [2, 5, 6], chloride sensing [7, 8], singlet oxygen photosensitization [9, 10], and molecular switching [11].

The cyclophane host **B** (Figure 1a), introduced by Chiu and co‐workers [12, 13], can also complex squaraine dyes, but only in the presence of sodium ions. Other alkali metal ions do not act as templates, and squaraine/host mixtures can be used for the selective optical detection of Na^+^ [13].

Herein, we describe a metallosupramolecular host for squaraine dyes with a distinct selectivity profile (Figure 1b). The receptor itself is non‐luminescent. Upon excitation, ultrafast (sub‐ps) dark resonance energy transfer (DRET) to the squaraine dyes is observed, resulting in bright, near‐infrared emission of the guest chromophore. The findings highlight the potential of metallosupramolecular assemblies in the development of noncovalent DRET systems.

## 2 Results and Discussion

**SCHEME 1:**
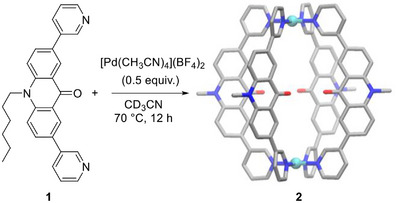
Synthesis of receptor **2**. The structure of the product is based on a crystallographic analysis. Hydrogen atoms, the hexyl side chains, and anions are not shown. Color coding: Pd cyan, C gray, N blue, O red.

For our investigations, we have used the dipyridyl ligand **1** (Scheme 1). The ligand features a highly fluorescent acridone core and two terminal 3‐pyridyl groups. The coordinate vectors of the latter can adopt a co‐planar arrangement, which is well‐suited for the construction of Pd_2_L_4_‐type coordination cages [14, 15]. Ligand **1** was obtained by a Suzuki coupling reaction, and details about its synthesis are given in the Supporting Information, section 2.1.

When a mixture of ligand **1** and [Pd(CH_3_CN)_4_](BF_4_)_2_ (0.5 equiv.) in CD_3_CN was heated to 70°C for 12 h, the formation of a defined complex (**2**) with high apparent symmetry was observed by NMR spectroscopy (see the Supporting Information, Figure S28). Mass spectrometry supported the formation of a dinuclear complex, with dominant peaks for a species of the formula {[Pd_2_(**1**)_4_](BF_4_)*_n_*}^(4–^ *^n^* ^)+^ (*n* = 0, 1) (see the Supporting Information, Figure S27). The solid‐state structure of complex **2** was determined by single‐crystal X‐ray diffraction (XRD), and a simplified graphic representation of the structure is depicted in Scheme 1.

The two Pd^2+^ ions in **2** are bridged by four dipyridyl ligands **1**. The planes defined by the pyridyl groups are slightly tilted with respect to the planes defined by the acridone groups (tilt angle: 30.5–34.4°).

Pd_2_L_4_‐type assemblies are known to have electropositive pyridyl NC–H protons, which can act as hydrogen bond donors to anions or electron‐rich moieties of neutral molecules [16, 17, 18, 19, 20, 21, 22, 23]. Based on the Pd···Pd distance of 12.2(1) Å, which was observed for **2**, we hypothesized that squaraine dyes might be suited guests for this cage.

**SCHEME 2:**
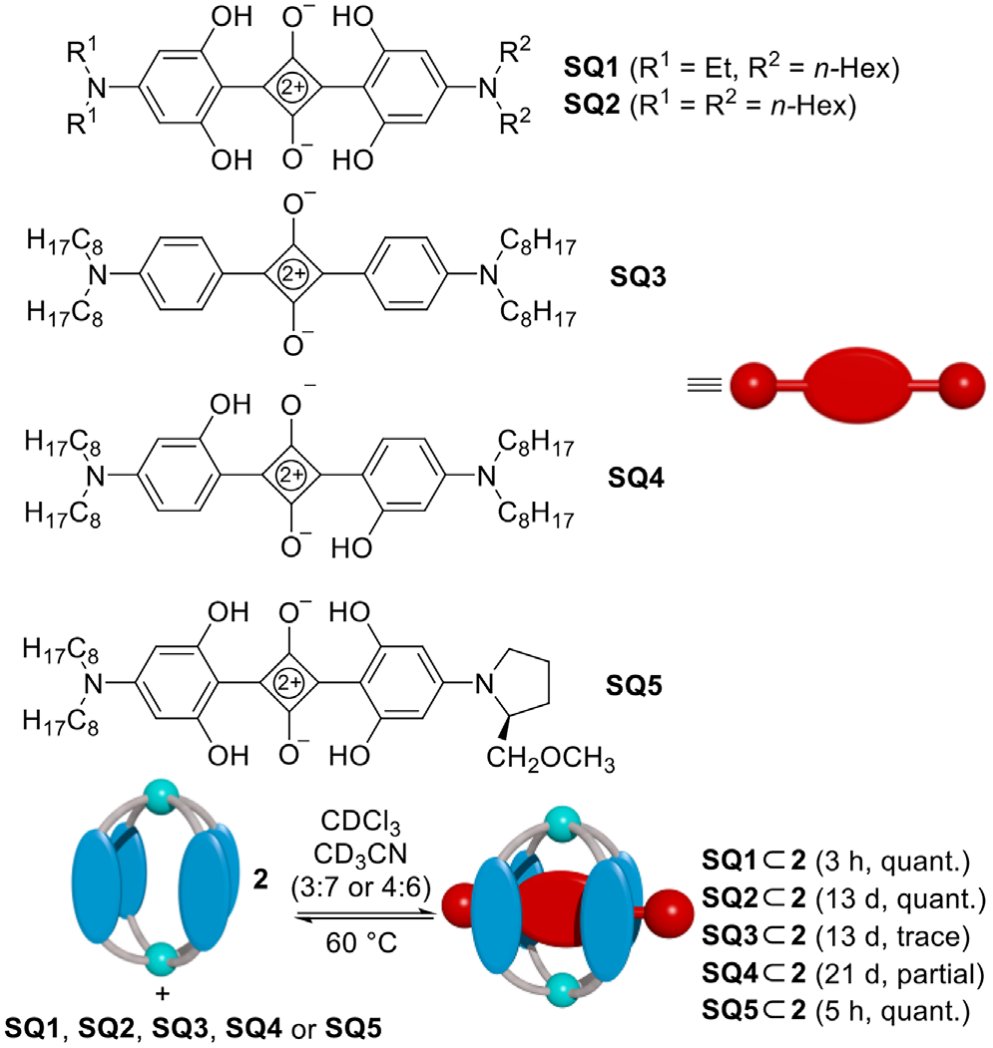
Binding of the squaraine dyes **SQ1–5** by cage **2**, as determined by ^1^H NMR spectroscopy.

Binding studies were performed with five different squaraine dyes, **SQ1**–**5**, the structures of which are depicted in Scheme 2. The dyes **SQ1** and **SQ2** have 2,6‐dihydroxy‐4‐(dialkylamino)phenyl groups attached to the central core. The ethyl and hexyl side chains at the amines were added to improve the solubility of the dyes in organic solvents. Squaraine dye **SQ3** has simple 4‐(di‐*n*‐octylamino)phenyl groups, and **SQ4** has 2‐hydroxy‐4‐(di‐*n*‐octylamino)phenyl substituents. In dye **SQ5**, we have introduced one chiral, proline‐derived side chain, along with solubilizing alkyl side chains.

Host‐guest studies were performed as follows: a solution of equal amounts of receptor **2** and the respective squaraine dye in a mixture of CDCl_3_ and CD_3_CN (3:7 or 4:6) was heated to 60°C, and the uptake was followed by ^1^H NMR spectroscopy.

For the tetra‐hydroxy squaraine dyes **SQ1**, **SQ2**, and **SQ5**, we observed the quantitative formation of the adducts **SQ1**⊂**2**, **SQ2**⊂**2**, and **SQ5⊂2** (see the Supporting Information, Figures S81, S83, and S87). **SQ2**⊂**2** retained the original *D*_4h_ symmetry of receptor **2**. The complexation of the asymmetric dye **SQ1** led to a reduced apparent symmetry of **2**, with twofold splitting of the ^1^H NMR signals, whereas complexation of **SQ5** caused an eightfold splitting (see the Supporting Information, Figures S47, S37, and S73). The reduced symmetry indicates that the dyes cannot rotate when bound to receptor **2**.

**FIGURE 2:**
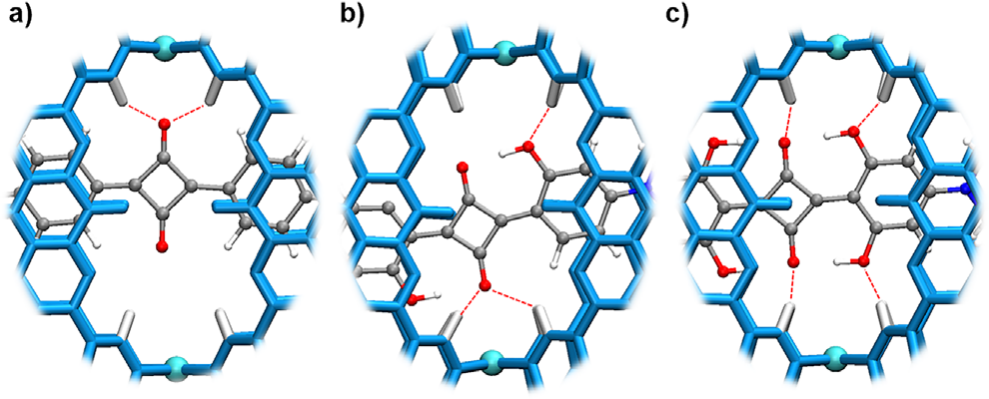
View of the central cavities of the xTB‐optimized structures of the adducts **SQ3⊂2** (a), **SQ4⊂2** (b), and **SQ2⊂2** (c).

Dye **SQ4** was only partially bound after thermal equilibration. By integration of ^1^H NMR signals corresponding to the ‘free’ receptor **2**, ‘free’ guest **SQ4** and **SQ4⊂2**, we were able to derive an association constant of *K_a_* = 1.6 × 10^4^ M^−1^. **SQ3** was only encapsulated in trace amounts after 2 weeks, and longer heating led to decomposition of the dye. The results suggest that the hydroxy groups in 2,6‐position are of importance to achieve efficient binding. This observation is in agreement with the distances measured in xTB‐optimized structures of host‐guest complexes, showing that the additional hydroxy groups engage in hydrogen bonds to the NC–H protons of the receptor (Figure 2). It is worth noting that the reverse selectivity was reported for tetralactam hosts of type **A**. For these receptors, the introduction of *ortho* hydroxy groups on the dye resulted in diminished binding [7, 24].

The adducts **SQ1**⊂**2, SQ2**⊂**2, SQ4**⊂**2** and **SQ5**⊂**2** could also be synthesized by suspending a slight excess of the corresponding dyes in a CD_3_CN solution of **2**. After stirring at 70°C overnight and filtering off the remaining dyes, the cage‐dye adducts were obtained quantitatively. Encapsulation into cage **2** helped solubilizing these squaraine dyes, which are otherwise poorly soluble in acetonitrile.

The ^1^H NMR spectrum of **SQ1**⊂**2** in CD_3_CN showed two well‐separated signals for the OH protons at 13.7 ppm and 8.9 ppm, implying that there are two distinct chemical environments for the OH groups (see the Supporting Information, Figure S37). Similarly, the signals of the OH protons of **SQ5**⊂**2** appeared as two well‐separated groups, with two signals at 13.7 and 13.4 ppm, and two signals at 8.8 and 8.6 ppm (see the Supporting Information, Figure S73). The signal multiplicity suggested an off‐centered complexation of the dyes, as already indicated by the optimized geometries (Figure 2). Further support for such a binding mode was provided by the ^1^H‐^1^H NOESY spectra of **SQ1**⊂**2** and **SQ5**⊂**2** (see the Supporting Information, Figures S44 and S80).

A priori, the preferred off‐centered complexation of **SQ1** and **SQ5** could be a consequence of the asymmetric substitution pattern of the dyes (two different terminal amine groups), or due to an intrinsic preference for a low‐symmetry complexation. To investigate this, we examined if an off‐centered complex also represents the energy minimum of **SQ2**⊂**2**. When increasing the temperature to 45°C, the initially broad signals in the ^1^H NMR spectrum of **SQ2**⊂**2** (CD_3_CN) became sharp. On the other hand, upon cooling to –23°C, a reduced apparent symmetry was observed for both, the host and the guest (see the Supporting Information, Figure S49 and S50). Well‐separated signals of the OH protons were identified at 13.7 and 8.6 ppm. The results indicate that the symmetric dye **SQ2** is also bound preferentially in an off‐centered fashion, and that the high apparent symmetry at room temperature comes from fast exchange on the ^1^H NMR timescale.

**FIGURE 3:**
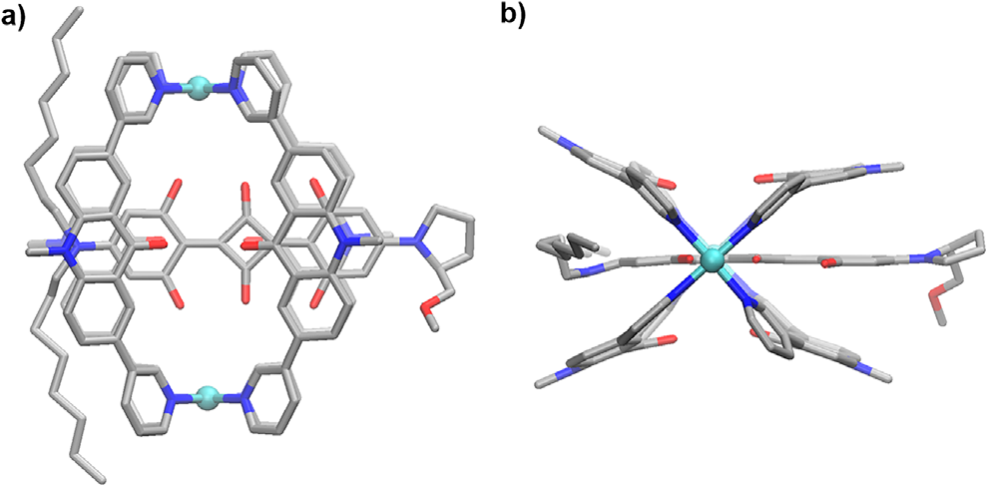
Molecular structure of **SQ5⊂2**, as determined by single‐crystal XRD, viewed from the side (a) and along the Pd···Pd axis (b). Hydrogen atoms, anions, and hexyl chains of the cage are not shown. Color coding: Pd cyan, C gray, N blue, O red.

Single crystals of **SQ5**⊂**2** were obtained by vapor diffusion of diethyl ether and ethyl acetate (1:1) into a solution of the host‐guest complex in CD_3_CN, and a crystallographic analysis was performed (Figure 3).

In agreement with the NMR data and with the optimized structures, only two of the four OH groups participate in hydrogen bonding to the pyridyl NC–H protons. Additional hydrogen bonds are observed between the NC–H protons and the two central O‐atoms of the dye [25]. The aromatic groups of the dye are sandwiched between the acridone groups of the ligand (Figure 3b), with approximately 3.6 Å distance between the planes.

The solid‐state structure of **SQ5**⊂**2** confirmed that the squaraine dye is bound by receptor **2** in a (pseudo)rotaxane‐like fashion [26]. From a geometrical analysis, it appears that the chiral arm of **SQ5** should be able to slide through the opening of the cage, whereas the bulky ‐NOct_2_ groups should impede the threading. This assumption is in line with the observation that the uptake of **SQ5** into receptor **2** is much faster than that of **SQ2** or **SQ4** (hours instead of weeks).

For the strongly bound dyes **SQ1**, **SQ2**, and **SQ5**, a determination of the association constants was hampered by the slow binding kinetics. The fastest complexation was observed for **SQ1**, and this dye was chosen for a quantitative binding analysis. Mixtures of **SQ1** (1.0 µM) and variable amounts of receptor **2** (0–50 µM) in CH_2_Cl_2_/CH_3_CN (1:9) were heated to 70°C for two hours to facilitate the uptake, and then stirred at room temperature for 12 h to allow equilibration. From the changes in the UV‐vis spectra upon variation of the cage concentration, we were able to derive an association constant of *K*_a_ = 7.8 × 10^4^ M^−1^.

**FIGURE 4:**
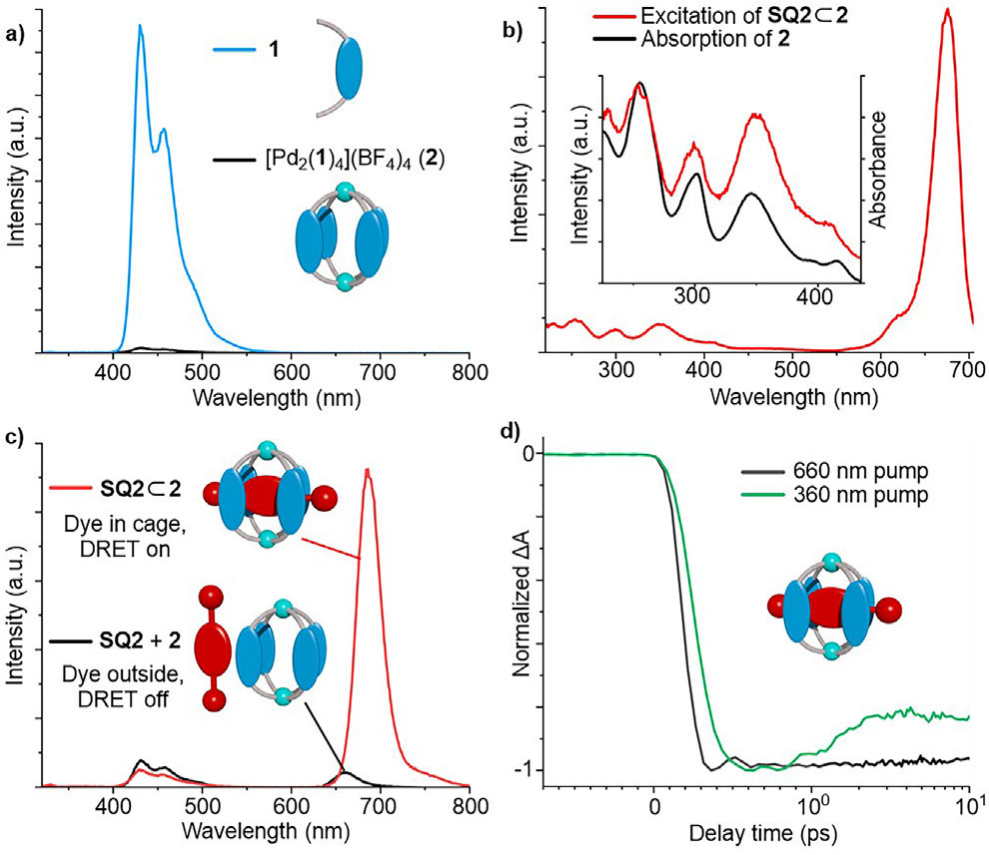
Emission spectra (CH_3_CN, *λ*_ex_ = 300 nm) of **1** (4.0 µM) and **2** (1.0 µM) (a). Excitation spectrum (CH_3_CN, *λ*_em_ = 715 nm) of **SQ2⊂2**, insert: zoom of the 225–430 nm region, overlayed with the absorption spectrum of **2** (b). Emission spectra (CH_3_CN, *λ*_ex_ = 300 nm) of **SQ2⊂2** (1.0 µM) and **SQ2 + 2** (1.0 µM each) (c). The excited state dynamics of **SQ2⊂2**, measured at a probe wavelength of 680 nm, after photoexcitation at 360 nm and 660 nm (d). To minimize dissociation, all measurements were performed immediately after dilution of concentrated stock solutions.

Palladium‐based cages are generally non‐luminescent [27]. Similarly, the fluorescence of ligand **1** was efficiently quenched upon coordination to Pd^2+^ (Figure 4a).

Solutions of the squaraine dyes **SQ1**, **SQ2**, or **SQ5** in CH_3_CN (1.0 µM) showed absorption and emission maxima at around 640 nm and 660 nm, respectively. When bound in cage **2**, both maxima were red‐shifted by approximately 25–30 nm (See the Supporting Information, Table S1). A similar red‐shift upon encapsulation was also reported for tetralactam macrocycles [2, 3, 4, 5, 6, 7, 9, 10, 11].

The excitation spectrum of the cage‐dye adducts showed not only a band around 675 nm, characteristic of direct excitation of the encapsulated squaraine dyes, but also sharp features in the UV region, with three new maxima at 253 nm, 300 nm, and 350 nm (Figure 4b). These features coincide with the absorption pattern of the cage. Excitation of the host‐guest complexes at any of these three wavelengths results in a bright red emission from the dyes, reaching pseudo‐Stokes shifts of up to 440 nm (Figure 4c, and Supporting Information, Figure S106). This observation suggests resonance energy transfer from the acridone‐based ligand to the squaraine dyes. Encapsulation of the dyes was found to be a prerequisite for efficient energy transfer: mixtures of the receptor **2** and ‘free’ squaraine dyes showed very weak emission when excited at these wavelengths (Figure 4c).

The utilization of donors with an internal quenching mechanism in resonance energy transfer pairs was first reported by Chang and co‐workers in 2014 [28]. The pairs were constructed by covalent linkage of a low quantum yield donor to a bright emitter. Resonance energy transfer was found to outcompete the internal quenching of the donor, resulting in efficient acceptor emission upon excitation of the donor. Since then, several dark resonance energy transfer (DRET) systems have been developed [29, 30, 31, 32, 33, 34, 35, 36, 37, 38, 39, 40]. Attractive spectroscopic features of such systems are large pseudo‐Stokes shifts and minimal fluorescence leakage from the donors. Most reported DRET pairs rely on covalently linked donors and acceptors, where donor emission is suppressed *via* internal rotation [29, 30, 31, 32, 33, 34, 35, 36, 37, 38, 39]. The use of metallosupramolecular assemblies for DRET is unprecedented.

The rates of fluorescence quenching of receptor **2** and energy transfer in the host‐guest complex **SQ2**⊂**2** were examined by ultrafast transient absorption spectroscopy. In both samples, the ground state bleaching (GSB) of the cage after photoexcitation at 360 nm was either not resolved, or masked by stronger photo‐induced absorption (PIA). A plausible explanation is that the depopulation of the ligand's excited state happens on sub‐300 fs timescales [41, 42, 43]. The rate of energy transfer in **SQ2**⊂**2** could be estimated from the GSB of the encapsulated dye: after photoexcitation at 360 nm (exciting the cage) or 660 nm (directly exciting the dye), the rise time of the GSB signal at 680 nm was 0.6 ps and 0.4 ps, respectively (Figure 4d). The difference between the two should correspond to the rate of energy transfer. It is worth noting that the energy transfer in this system is fast compared to most DRET or FRET systems [28, 40, 44, 45, 46, 47]. We attribute this observation to the very short distance between the donor and acceptor.

We also investigated how the chirality of **SQ5**⊂**2** might give rise to chiroptical properties. A circular dichroism (CD) dissymmetry factor of *g* = 1.0 × 10^−4^ (peak value at 377 nm) was found for the encapsulated dye (see the Supporting Information, Figure S108). The maximum signal corresponds to an absorption of the cage, indicating chirality transfer from **SQ5** to the receptor. No measurable circularly polarized luminescence could be detected within instrument sensitivity [48].

## 3 Conclusions

To conclude: a novel high‐affinity receptor for squaraine dyes has been developed. The receptor can be accessed in a straightforward manner by thermal equilibration of an acridone‐based dipyridyl ligand with Pd^2+^. Comprehensive binding studies have revealed a pronounced preference for dyes with 2,6‐dihydroxyphenyl substituents. This selectivity profile differs markedly from that reported for tetralactam macrocycles, which are the most extensively studied squaraine receptors.

The receptor itself is non‐emissive. Excitation of the acridone scaffold triggers ultrafast dark resonance energy transfer to the encapsulated squaraine dyes, resulting in bright, near‐infrared emission, with large pseudo‐Stokes shifts of up to 440 nm. The findings demonstrate that metallosupramolecular assemblies offer significant potential for the design of DRET systems.

## Conflicts of Interest

The author declares no conflicts of interest.

## Supporting information




**Supporting File 1**: anie71340‐sup‐0001‐SuppMat.pdf.

## Data Availability

The data that supports the findings of this study are available in the supplementary material of this article
