# Supplementary material for: A Metallosupramolecular Receptor for Squaraine Dyes Enabling Ultrafast Dark Resonance Energy Transfer
Source: Angew Chem Int Ed Engl. 2026 Feb 1;65(11):e2203782. doi: 10.1002/anie.2203782 (PMC12970521; doi:10.1002/anie.2203782)
Supplement: Supplementary file 1 — Supporting File 1: anie71340‐sup‐0001‐SuppMat.pdf. [file ANIE-65-e2203782-s001.pdf]

A Metallosupramolecular Receptor for Squaraine Dyes Enabling Ultrafast Dark Resonance Energy Transfer

Damien. W. Chen, Tejas Deshpande, Sybille Collignon, Farzaneh Fadaei-Tirani, Sascha Feldmann, and Kay Severin*

D. W. Chen, T. Deshpande, S. Collignon, F. Fadaei-Tirani, S. Feldmann, and K. Severin

Institut des Sciences et Ingénierie Chimiques, Ecole Polytechnique Fédérale de Lausanne (EPFL), 1015, Lausanne, Switzerland

E-mail: kay.severin@epfl.ch

Table of Contents

1. **General** 2

2. **Syntheses and characterization** 4

   2.1. **Ligand and dyes** 4

   2.2. **Cage and host-guest complexes** 20

3. **Host-guest studies** 54

   3.1. **NMR titration experiments** 54

   3.2. **UV-vis absorption titration of SQ1 + 2.** 62

4. **Photophysical properties** 63

   4.1. **UV-vis absorption and fluorescence** 63

   4.2. **Unthreading kinetics monitored by absorption and fluorescence** 69

   4.3. **Dark resonance energy transfer in SQ2⊂2** 71

   4.4. **Photoluminescence quantum yield** 73

   4.5. **Circular dichroism** 73

   4.6. **Ultrafast transient absorption** 74

5. **Crystallographic Data** 75

   5.1. **$[Pd_2(1)_4](CF_3SO_3)_4$** 75

   5.2. **$[Pd_2(1)_4(SQ5)](BF_4)_4$** 77

6. **Computational details** 80

7. **References** 82

1. General

All reagents were obtained from commercial sources and used without further purification unless stated otherwise. 2,7-dibromo-10-hexylacridin-9(10H)-one[1] di-*n*-octylaniline[2] and **SQ2**[3] were synthesized following procedures reported in the literature.

NMR spectra were measured on a Bruker Avance III HD spectrometer (^{1}H: 600 MHz, ^{13}C: 151 MHz) equipped with a Prodigy BBFO$_z$ 5 mm cryoprobe, a Bruker Avance III spectrometer (^{1}H: 400 MHz) equipped with a BBFO$_z$ 5 mm probe, a Bruker Avance III spectrometer (^{1}H: 400 MHz) equipped with a Prodigy BBO 5 mm cryoprobe and a Bruker Avance II spectrometer (^{1}H: 800 MHz) equipped with a 5 mm CPTCI$_{xyz}$ cryoprobe. The chemical shifts are reported in parts per million (ppm) using the solvent residual signal as a reference. The spectra were recorded at 298 K if not stated otherwise. The acquisition time and relaxation delay for quantitative 1H NMR measurements were set to > 4 seconds and > 10 seconds respectively.

Mass spectrometric analyses were conducted using an LTQ Orbitrap Elite FTMS instrument (Thermo Scientific, Bremen, Germany) operated in positive ion mode and equipped with a robotic chip-based nanoelectrospray ionization (nano-ESI) source (TriVersa Nanomate, Advion Biosciences, Ithaca, NY, USA). Data acquisition and instrument control were performed using the standard Thermo Scientific software suite, while the ion source was operated via Chipsoft 8.3.1 (Advion Biosciences). Samples (5 µL) were diluted in acetonitrile and loaded onto a 96-well plate (Eppendorf, Hamburg, Germany). The ionization voltage was set to +1.2 kV, with a gas pressure of 0.30 psi, and the ion transfer capillary maintained at 80 °C. FTMS spectra were acquired in the 200–3000 m/z range in reduced profile mode, with a resolution of 120k at m/z 400. Each spectrum was recorded with one microscan and a maximum injection time of 1000 ms.

UV-Vis spectra were acquired on a Cary 60 spectrometer (Aglient Technologies) equipped with a multiple cuvette holder coupled to a thermostat, which was calibrated to hold a temperature of 298 K using ethanol as circulating fluid. Emission and excitation spectra were recorded on a Cary Eclipse fluorescence spectrometer (Varian). Second harmonic artifacts were suppressed using the “auto” option for excitation and emission filters.

The Femtosecond transient absorption (fs-TA) experiments were performed using a setup based on modules supplied by Light Conversion, with a 1030 nm seed laser (PHAROS, Light Conversion, Yb:KGW lasing medium, 340 mW pulse energy, 50 fs duration, operated at 10 kHz repetition rate). The pump beam (360 nm or 660 nm, ca. 650 µm in diameter) was generated using an optical parametric amplification (OPA) unit (ORPHEUS, Light Conversion), which generated a signal and an idler from the fundamental 1030 nm seed (fluence ca. 5.5 µJ/cm^2). The probe beam (ca. 200 µm in diameter) was generated from the second harmonic of the fundamental, or the fundamental, using supercontinuum generation in a sapphire crystal. The probe

generated from the 515 nm second harmonic spanned from ca. 380–500 nm. The probe generated from the 1030 nm fundamental spanned from ca. 500–900 nm. The pump-probe delay was controlled over a range of 7 ns by changing probe path length via a multi-pass delay stage, and the pump was passed through an optical chopper (75 Hz), where a beamsplitter/photodiode combination was used to divide and sort measurements into pumped and unpumped. The probe beam was passed into a grating spectrograph (Andor Kymera 193i) and recorded using a Si NMOS photodiode array detector (256 pixels). The broadband transient absorption spectra were chirp corrected during post-processing by fitting a third order polynomial to the onset of the signal at very early (sub-ps) timescales.

Steady-state photoluminescence quantum yield measurements in solution (CH_3CN or CH_3CN/CH_2Cl_2 (9:1)) were performed using a commercial FLS1000 (Edinburgh Instruments), equipped with a xenon lamp (continuous-wave) and a Czerny-Turner type monochromator (1800 grooves/mm) as part of the excitation arm, allowing tunable excitation wavelength. The sample in solution (1 cm cuvette pathlength) was placed in a commercial integrating sphere (Edinburgh Instruments) coated with polytetrafluoroethylene and equipped with a focusing lens and a collection lens. After the collection lens, a Czerny-Turner type monochromator and a photomultiplier tube (PMT900) were used as the detection arm. The excitation wavelength was chosen as 350 nm. The sample cuvette and a corresponding blank solvent cuvette were measured twice each for their emission to ensure reproducibility.

The circular dichroism (CD) spectra of the samples in solution were recorded using a JASCO J-1500 spectrometer equipped with the standard PMT detector (163–950 nm). Spectra were collected between 800 and 250 nm with a 1 nm step size and a 1 second integration time. All CD spectra were corrected for linear dichroism and linear birefringence artifacts by recording the spectra for four different sample orientations.

2. Syntheses and characterization

2.1. Ligand and dyes

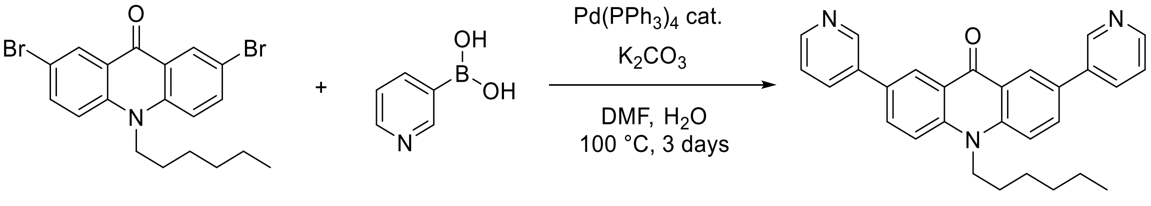

Scheme S1. Synthesis of **1**.

Ligand **1** was synthesized in analogy to a reported procedure.[4] A suspension of 2,7-dibromo-10-hexylacridin-9(10H)-one (284 mg, 650 μmol, 1 equiv.), 3-pyridyl boronic acid (243 mg, 1.98 mmol, 3 equiv.) and K_2CO_3 (916 mg, 6.63 mmol, 10 equiv.) in N,N-dimethylformamide (20 mL) and H_2O (4 mL) was degassed by vacuum/nitrogen cycles. $Pd(PPh_3)_4$ (50 mg, 0.043 mmol, 0.07 equiv.) was added, and the mixture was stirred at 100 °C under nitrogen for 3 days. The product was extracted with chloroform (3 × 40 mL), the combined organic fractions were dried over $MgSO_4 \cdot 2H_2O$ and concentrated under reduced pressure to give a dark yellow solid. The crude product was separated by column chromatography (100% ethyl acetate to ethyl acetate/methanol (9:1)), yielding **1** as a yellow solid that has blue fluorescence (180 mg, 0.42 mmol, 64%). ^{1}H NMR (400 MHz, $CDCl_3$) δ 8.99 (dd, *J* = 2.4, 0.9 Hz, 2H), 8.86 (d, *J* = 2.4 Hz, 2H), 8.63 (dd, *J* = 4.8, 1.6 Hz, 2H), 8.10 – 7.99 (m, 4H), 7.67 (d, *J* = 9.0 Hz, 2H), 7.42 (ddd, *J* = 8.0, 4.8, 0.9 Hz, 2H), 4.45 (t, 2H), 2.07 – 1.96 (m, 2H), 1.66 – 1.61 (m, 2H), 1.53 – 1.37 (m, 4H), 0.97 (t, *J* = 7.0 Hz, 3H) (Figure S1). ^{13}C NMR (151 MHz, $CDCl_3$) δ 177.91, 148.74, 148.21, 141.53, 135.30, 134.36, 132.69, 131.09, 126.37, 123.90, 123.04, 115.96, 46.68, 31.69, 27.48, 26.79, 22.82, 14.17 (Figure S2). HRMS (ESI/QTOF) *m*/*z*: $[M + H]^+$ Calcd for $C_{29}H_{28}N_3O^+$ 434.2227; Found 434.2226.

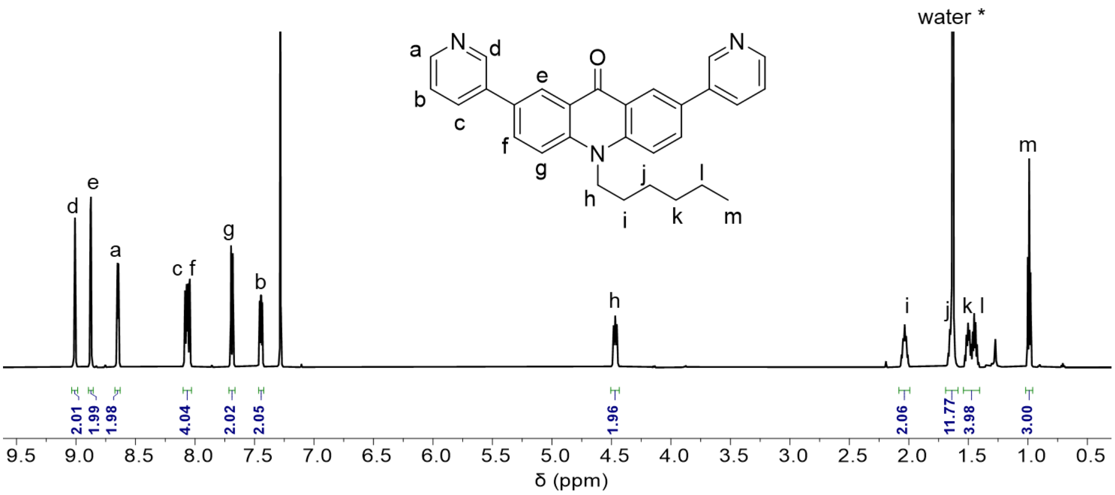

Figure S1. ^{1}H NMR spectrum (600 MHz, $CDCl_3$) of **1**. *$CDCl_3$ was neutralized by passing it through alumina, resulting in the water signal at 1.56 ppm visible in all ^{1}H spectra in this section.

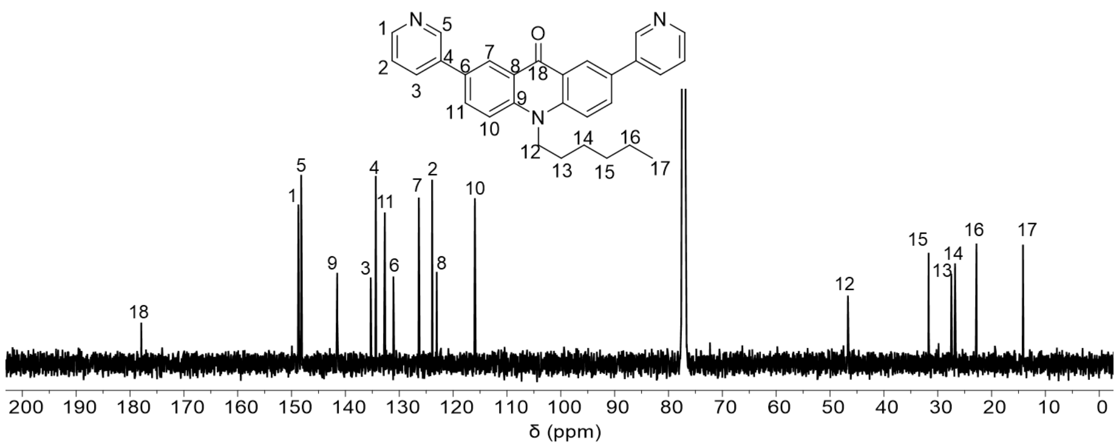

Figure S2. ^{13}C NMR spectrum (151 MHz, $CDCl_3$) of **1**.

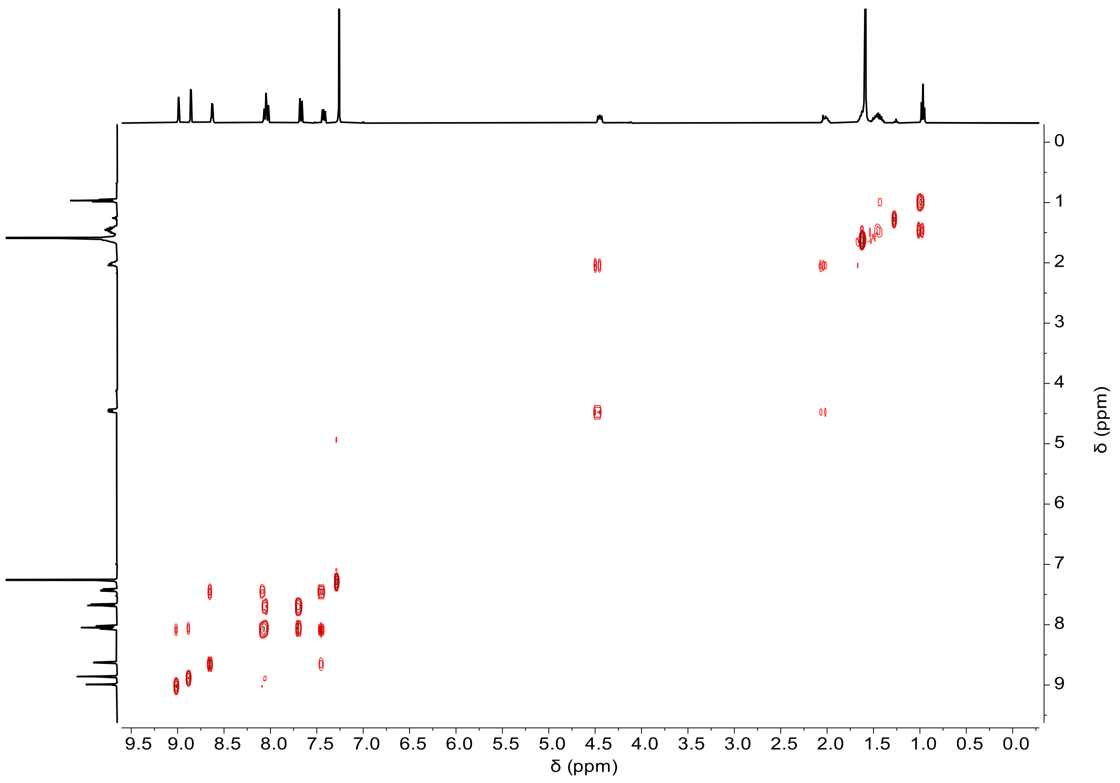

Figure S3. ^{1}H-^{1}H COSY NMR spectrum (400 MHz, $CDCl_3$) of **1**.

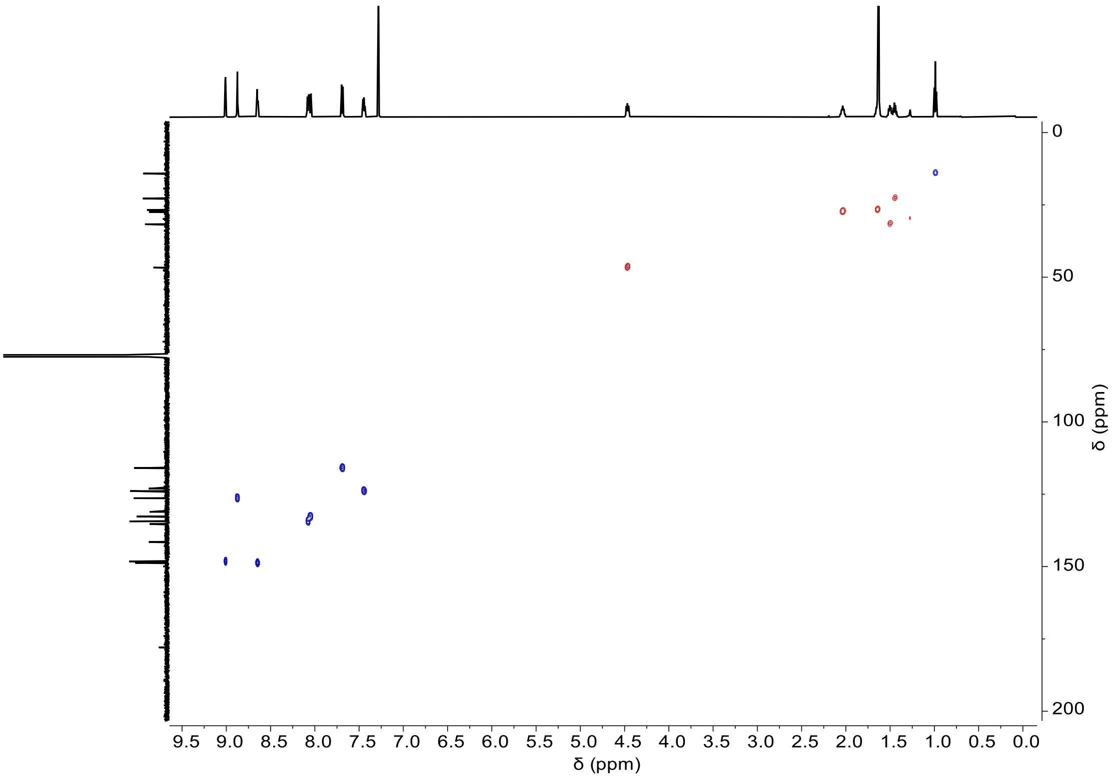

Figure S4. ^{1}H-^{13}C HSQC NMR spectrum (600 MHz, $CDCl_3$) of **1**.

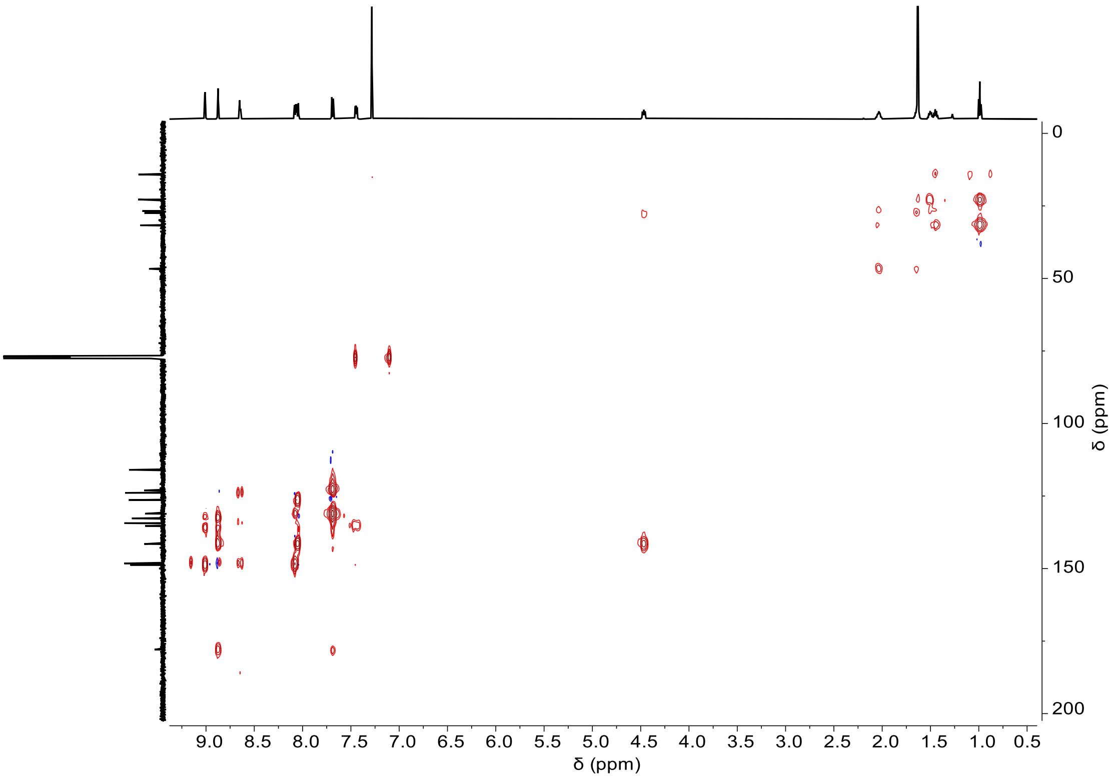

Figure S5. ^{1}H-^{13}C HMBC NMR spectrum (600 MHz, $CDCl_3$) of **1**.

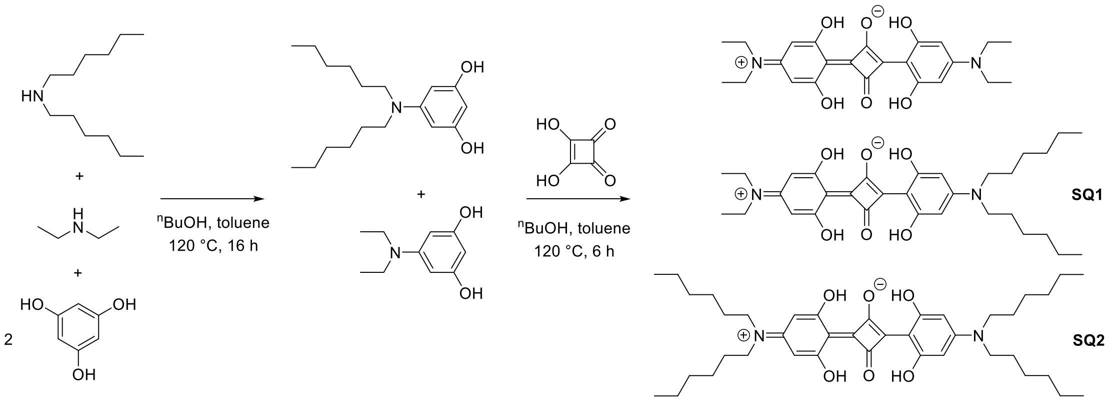

Scheme S2. Synthesis of **SQ1**

Di-n-hexylamine (578 mg, 3.1 mmol, 1 equiv.), diethylamine (0.32 mL, 3.1 mmol, 1 equiv.) and phloroglucinol (788 mg, 6.2 mmol, 2 equiv.) were dissolved in toluene (20 mL) and *n*-butanol (10 mL). The mixture was heated to reflux and stirred overnight, with water removed azeotropically *via* a Dean-Stark setup. The orange mixture was briefly cooled down to room temperature, and squaric acid (352 mg, 3.1 mmol, 1 equiv.) was added. After reaching reflux again, the mixture quickly turned blue and showed red fluorescence under ambient light. Heating was continued for 6 h. After cooling down to room temperature, solvents were concentrated under reduced pressure, MeOH (30 mL) was added, and the mixture was left overnight at –20 °C to promote precipitation. The green solid was isolated by filtration, and the three squaraine dyes were separated by column chromatography (hexane/DCM (8:2) to 100% DCM). **SQ1** was obtained from the second colored fraction as a green solid (214 mg, 0.39 mmol, 13%). ^{1}H NMR (400 MHz, $CDCl_3$) δ 10.99 (s, 2H), 10.97 (s, 2H), 5.80 (s, 2H), 5.77 (s, 2H), 3.43 (q, J = 7.1 Hz, 4H), 3.32 (t, J = 8.0 Hz, 4H), 1.68 – 1.58 (m, 4H), 1.36 – 1.29 (m, 12H), 1.24 (t, J = 7.1 Hz, 6H), 0.90 (t, J = 6.4 Hz, 6H) (Figure S6). ^{13}C NMR (151 MHz, $CDCl_3$) δ 181.46, 163.03, 162.93, 161.34, 161.23, 158.03, 157.63, 102.56, 102.44, 93.76, 93.54, 51.81, 45.52, 31.69, 28.11, 26.79, 22.75, 14.14, 13.30 (Figure S7). HRMS (ESI/QTOF) *m*/*z*: $[M + H]^+$ Calcd for $C_{32}H_{45}N_2O_6^+$ 553.3272; Found 553.3274.

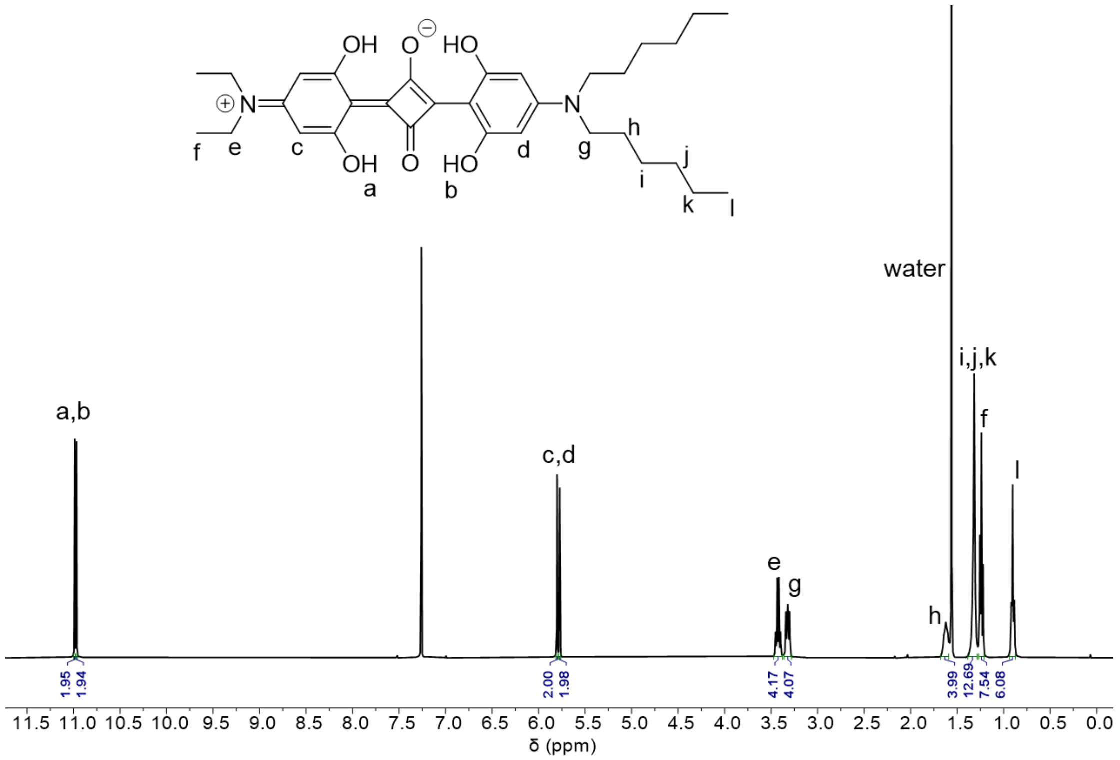

Figure S6. ^{1}H NMR spectrum (400 MHz, $CDCl_3$) of **SQ1**.

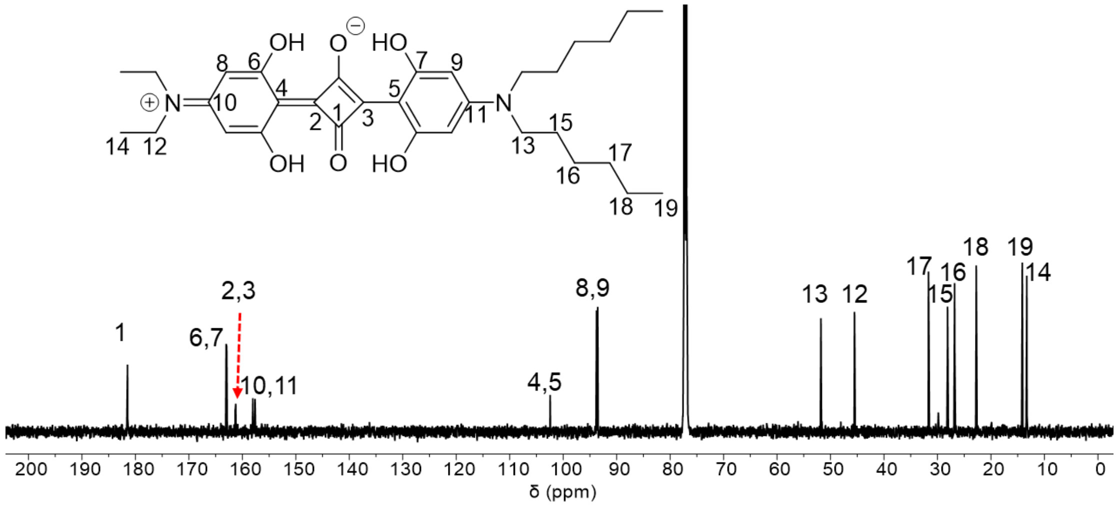

Figure S7. ^{13}C NMR spectrum (151 MHz, $CDCl_3$) of **SQ1**.

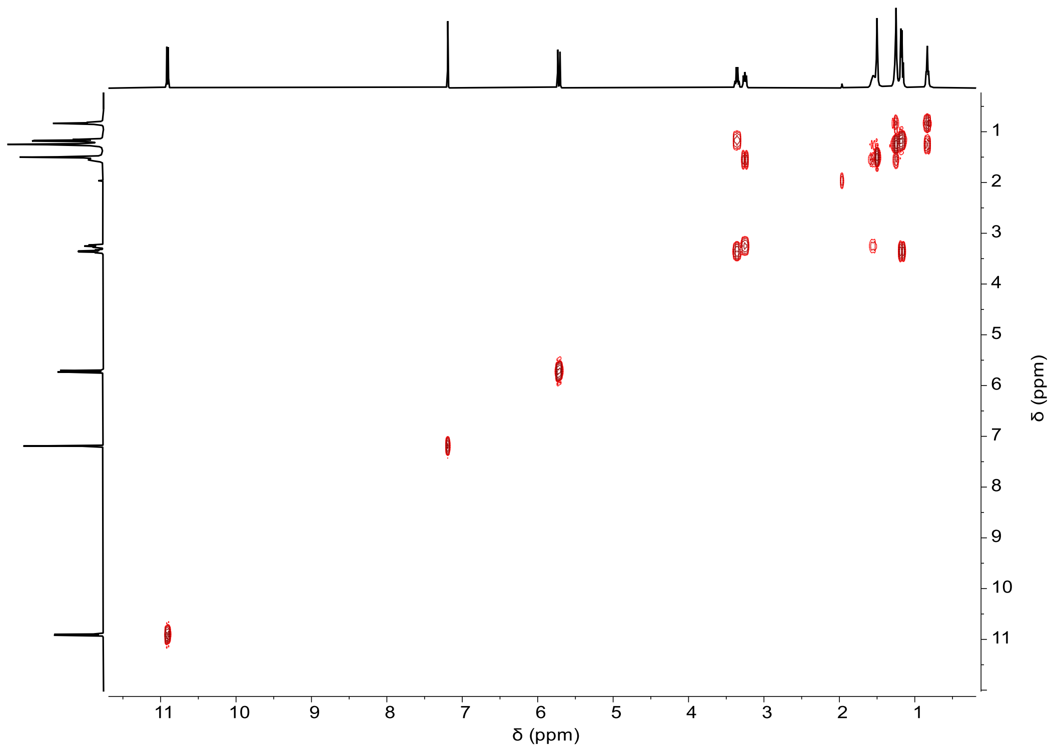

Figure S8. ^{1}H-^{1}H COSY NMR spectrum (400 MHz, $CDCl_3$) of **SQ1**.

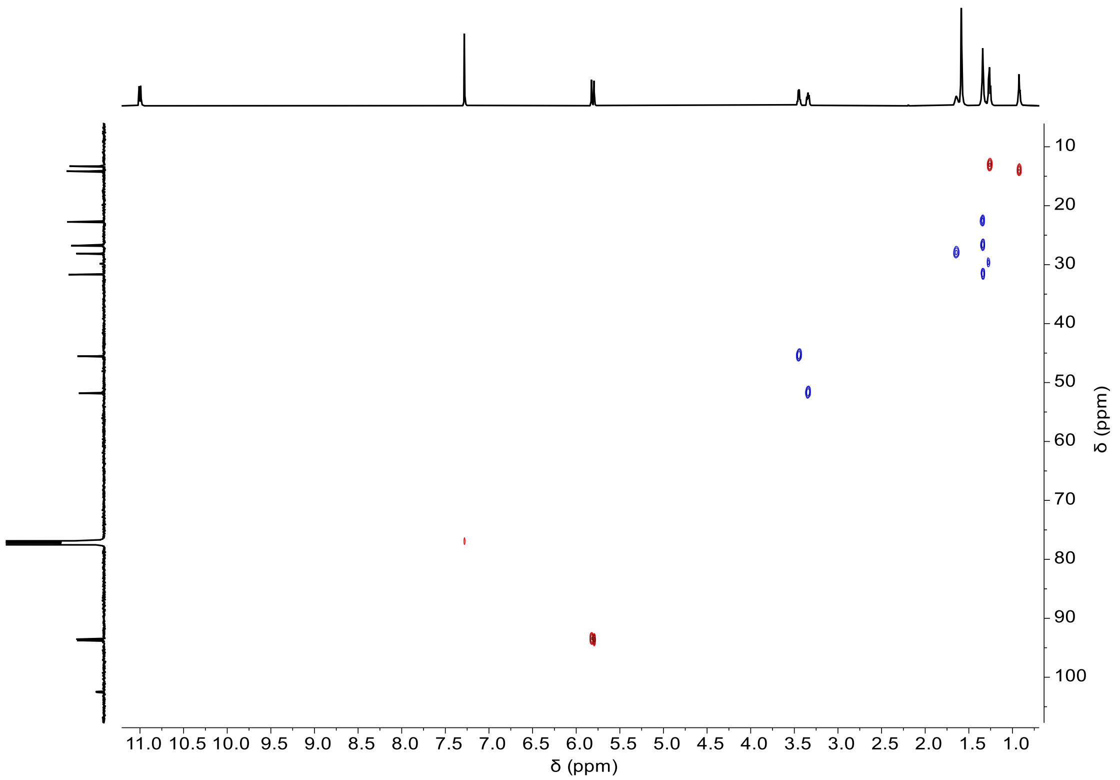

Figure S9. ^{1}H-^{13}C HSQC NMR spectrum (600 MHz, $CDCl_3$) of **SQ1**.

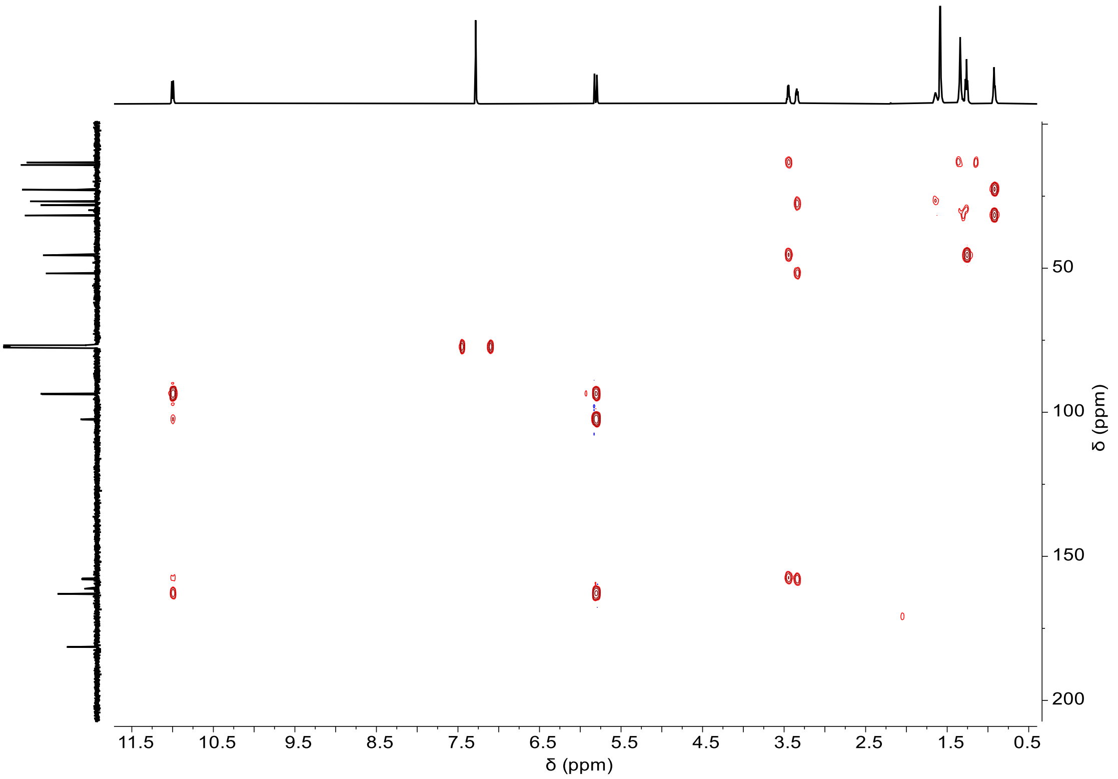

Figure S10. ^{1}H-^{13}C HMBC NMR spectrum (600 MHz, $CDCl_3$) of **SQ1**.

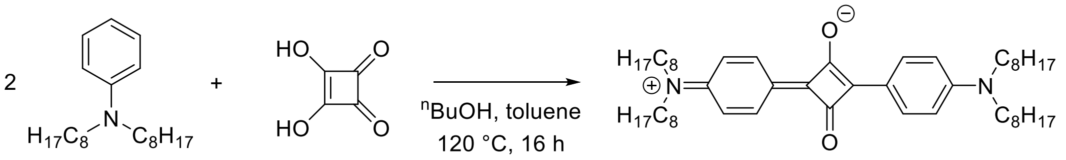

SQ3

Scheme S3. Synthesis of **SQ3**

Di-*n*-octylaniline (664 mg, 2.09 mmol, 2 equiv.) and squaric acid (120 mg, 1.05 mmol, 1 equiv.) were dissolved in toluene (15 mL) and *n*-butanol (15 mL). The mixture was heated to reflux and stirred overnight, with water removed azeotropically *via* a Dean-Stark setup. The dark blue solution was cooled to room temperature and concentrated under reduced pressure. The solids were isolated by filtration, and recrystallized from DCM and methanol, yielding **SQ3** as a shiny golden solid (217 mg, 0.30 mmol, 29%). ^{1}H NMR (400 MHz, $CDCl_3$) δ 8.35 (d, J = 9.1 Hz, 4H), 6.70 (d, J = 9.3 Hz, 4H), 3.46 – 3.36 (m, 8H), 1.64 (p, J = 7.1 Hz, 8H), 1.38 – 1.25 (m, 40H), 0.89 (t, 12H) (Figure S11). ^{13}C NMR (201 MHz, $CDCl_3$) δ 187.55, 183.67, 153.46, 133.34, 119.64, 112.37, 51.58, 31.90, 29.48, 29.36, 27.63, 27.14, 22.76, 14.23 (Figure S12). HRMS (nanochip-ESI/LTQ-Orbitrap) *m*/*z*: $[M]^+$ Calcd for $C_{48}H_{76}N_2O_2^+$ 712.5901; Found 712.5932.

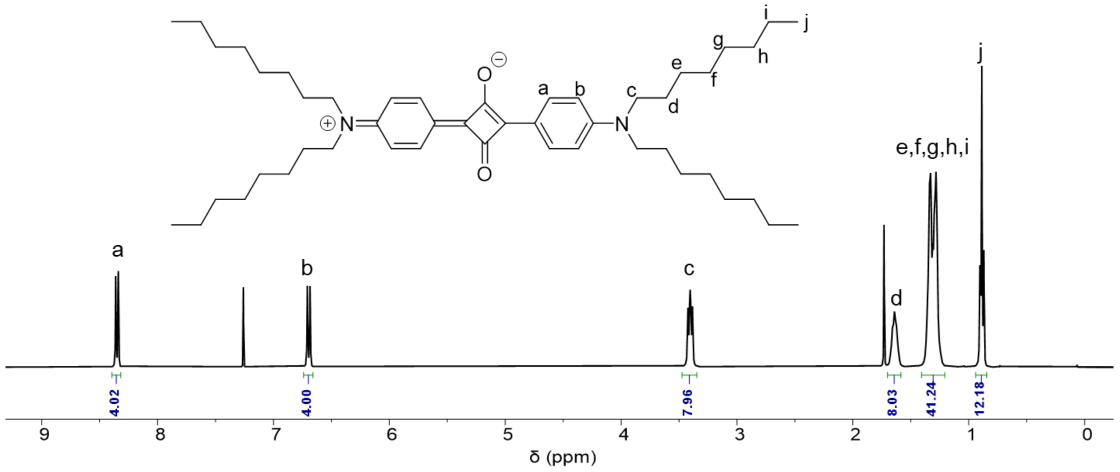

Figure S11. ^{1}H NMR spectrum (400 MHz, $CDCl_3$) of **SQ3**.

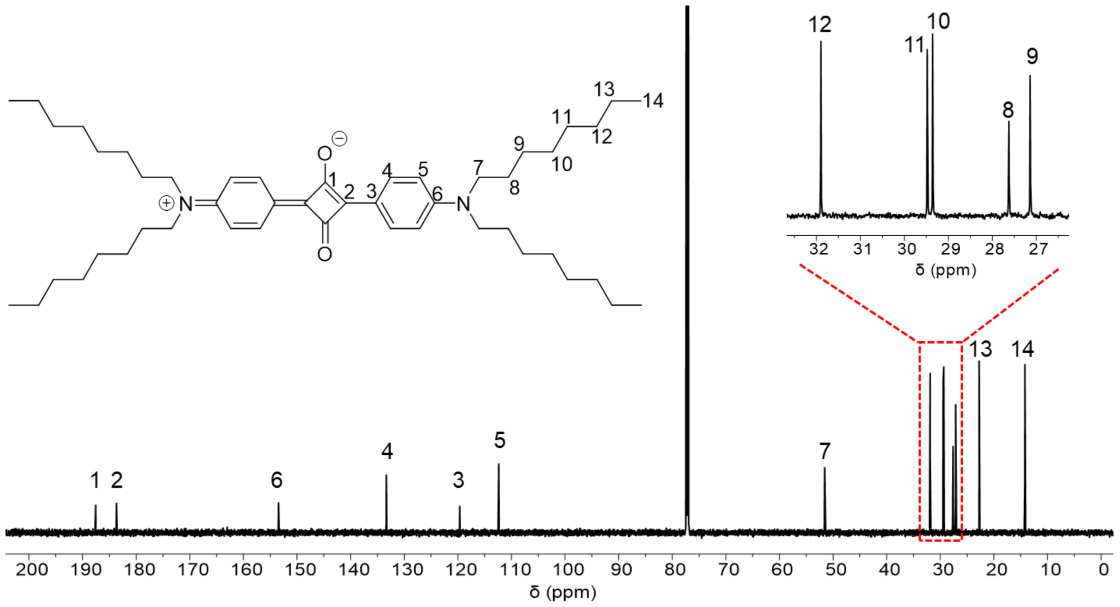

Figure S12. ^{13}C NMR spectrum (201 MHz, $CDCl_3$) of **SQ3**.

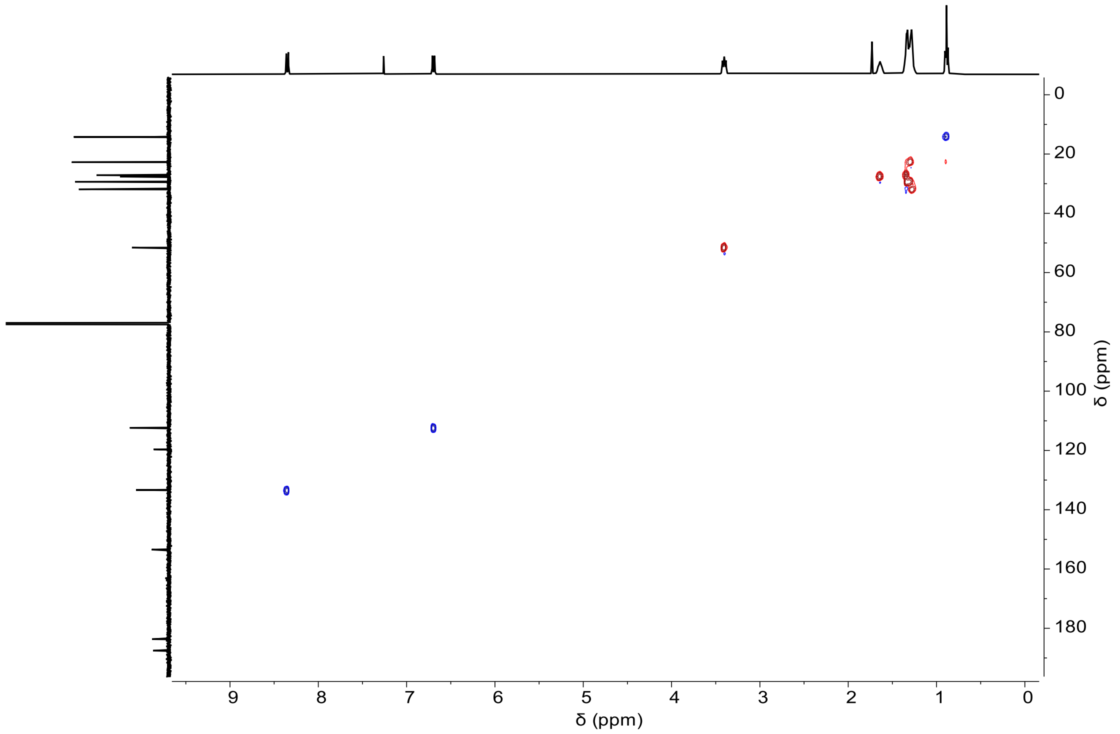

Figure S13. ^{1}H-^{13}C HSQC NMR spectrum (600 MHz, $CDCl_3$) of **SQ3**.

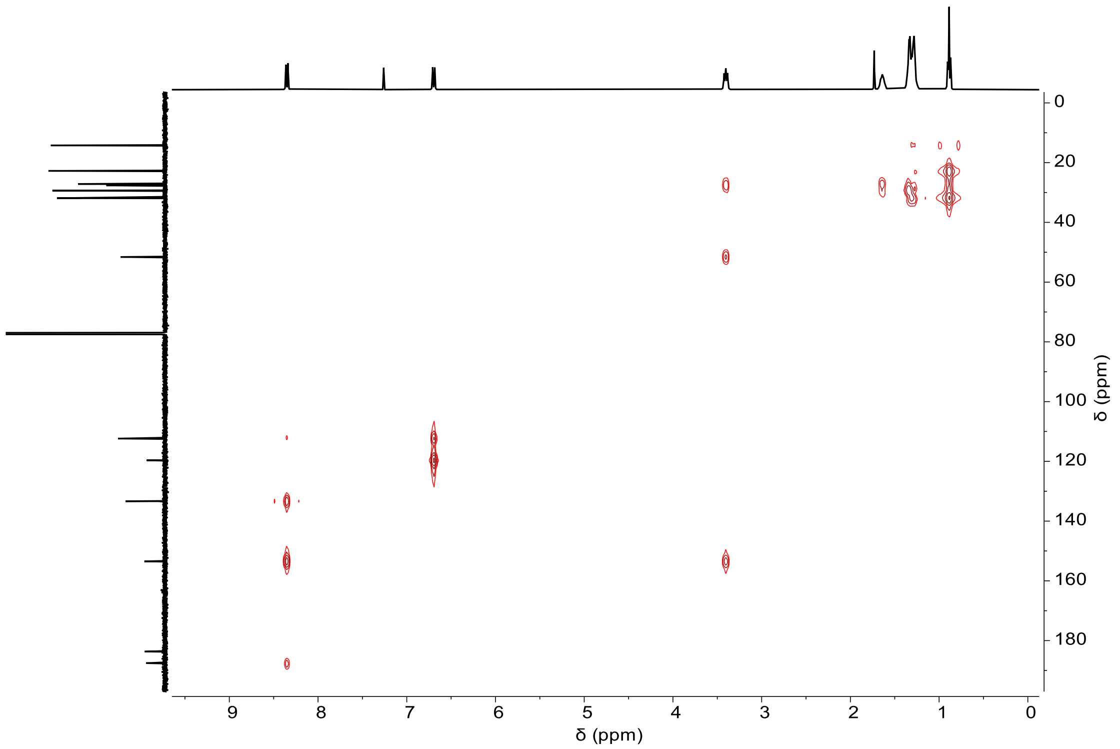

Figure S14. ^{1}H-^{13}C HMBC NMR spectrum (600 MHz, $CDCl_3$) of **SQ3**.

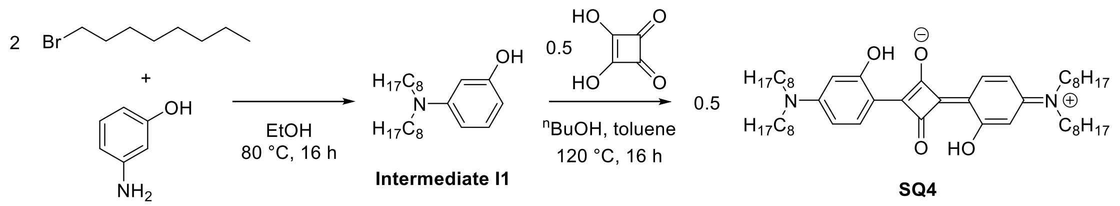

Scheme S4. Synthesis of **SQ4**

The synthesis of 3-(di-*n*-octylamino)phenol (**Intermediate I1**) was modified from a reported procedure.[5] Namely, a larger excess (3.4 equiv.) of 1-bromooctane was used in order to push the formation of the di-octylated product.

SQ4 was synthesized from **Intermediate I1** following a literature procedure,[6] and was obtained as a green powder with golden shine (377 mg, 0.51 mmol, 78%). ^{1}H NMR showed that the product was a 76:24 mixture of *trans* and *cis* isomers. The fact that the two isomers are in equilibrium, and that the *trans* form is the major isomer was previously studied by Kazmaier *et al.*[7] ^{1}H NMR (600 MHz, $CDCl_3$) δ 12.10 (s, (2*0.76)H), 11.39 (s, (2*0.24)H), 8.04 (d, *J* = 9.1 Hz, (2*0.24)H), 7.89 (d, *J* = 9.2 Hz, (2*0.76)H), 6.35 – 6.30 (m, (2*0.24)H), 6.14 – 6.08 (m, (2*0.24)H), 3.38 (t, *J* = 7.9 Hz, 8H), 1.68 – 1.62 (m, 8H), 1.38 – 1.24 (m, 40H), 0.91 (t, *J* = 6.9 Hz, 12H) (Figure S15). ^{13}C NMR (151 MHz, $CDCl_3$) δ 183.60, 183.17, 179.63, 173.70, 172.24, 164.51, 163.90, 156.51, 156.48, 132.94, 132.56, 109.94, 109.81, 107.72, 107.61, 98.64, 51.76, 31.91, 29.49, 29.37, 27.88, 27.14, 22.76, 14.23 (Figure S16). HRMS (ESI/QTOF) *m*/*z*: $[M]^+$ Calcd for $C_{48}H_{76}N_2O_4^+$ 744.5800; Found 744.5804.

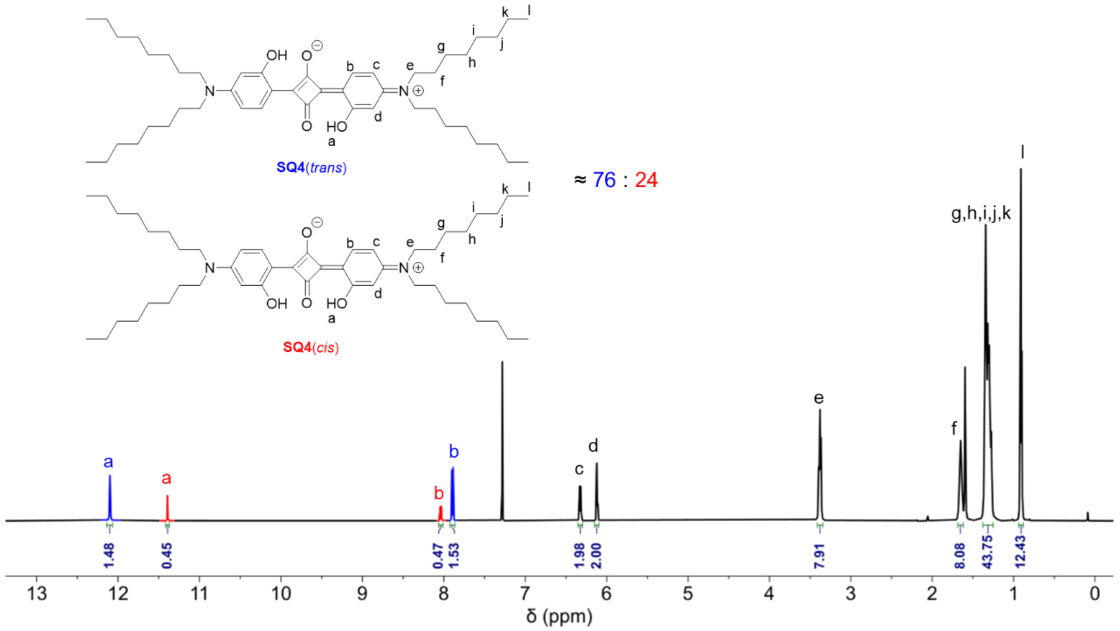

Figure S15. ^{1}H NMR spectrum (600 MHz, $CDCl_3$) of **SQ4**.

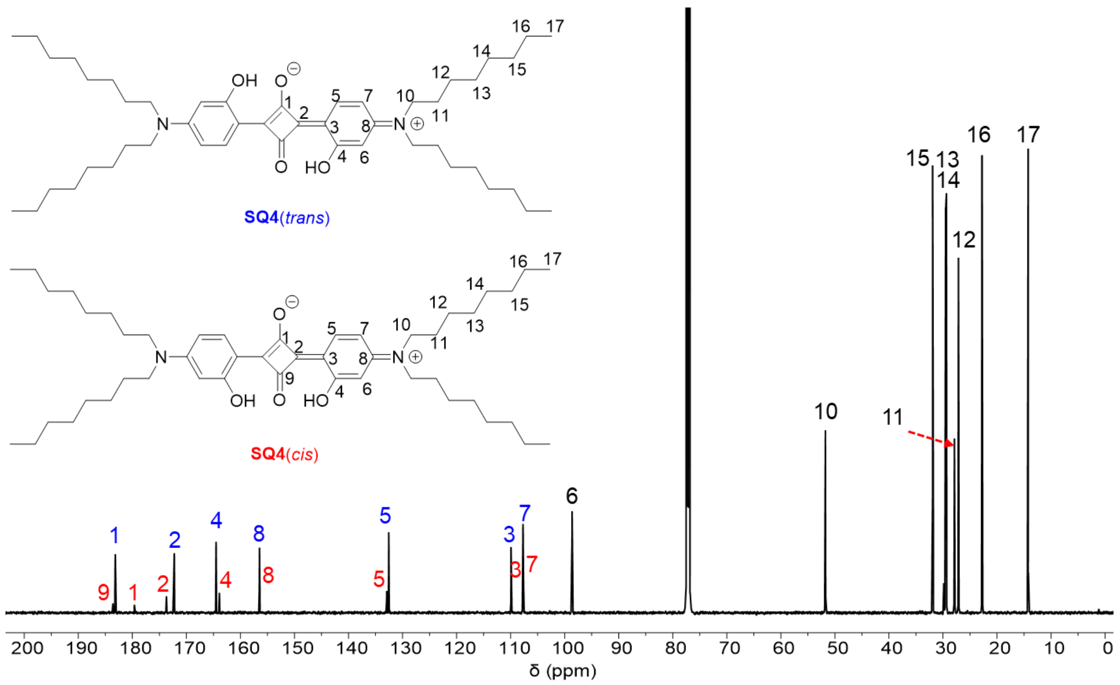

Figure S16. ^{13}C NMR spectrum (600 MHz, $CDCl_3$) of **SQ4**.

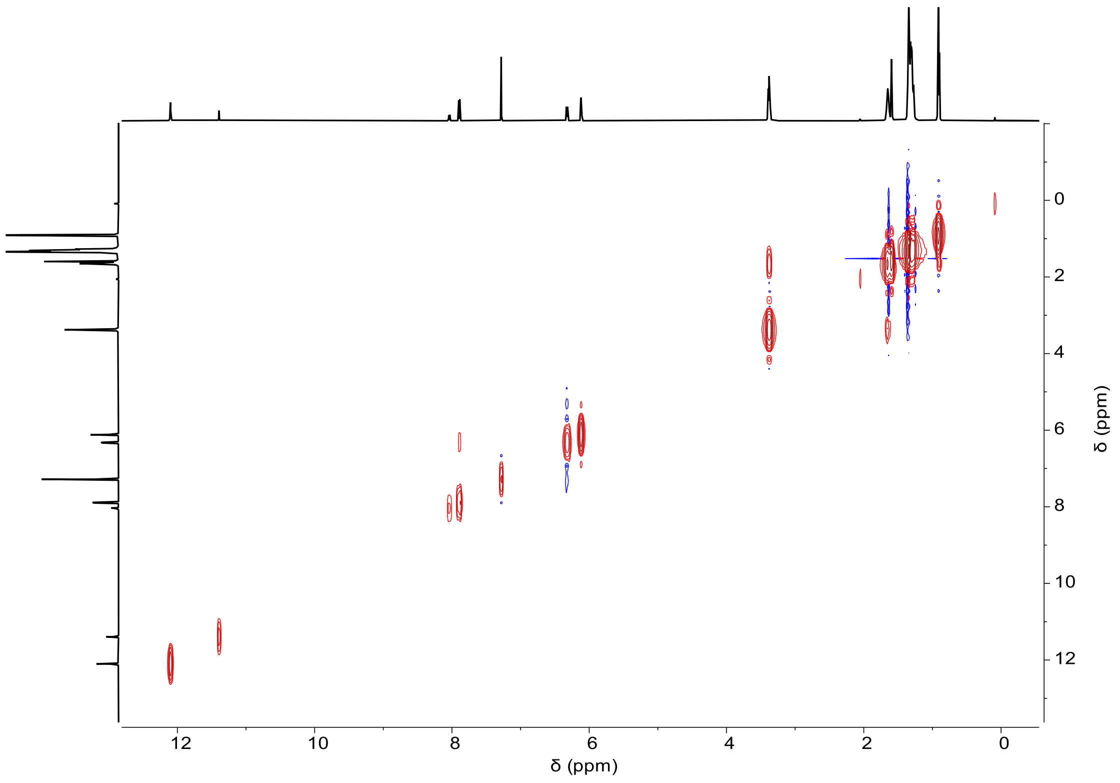

Figure S17. ^{1}H-^{1}H COSY NMR spectrum (600 MHz, $CDCl_3$) of **SQ4**.

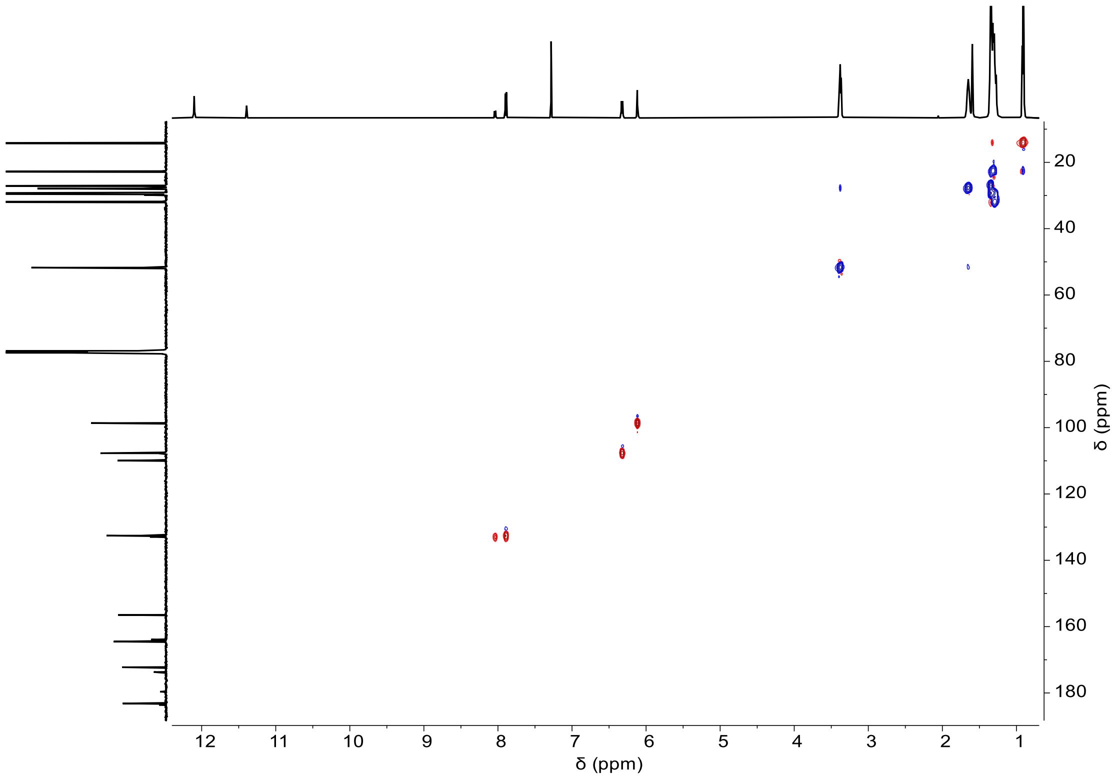

Figure S18. ^{1}H-^{13}C HSQC NMR spectrum (600 MHz, $CDCl_3$) of **SQ4**.

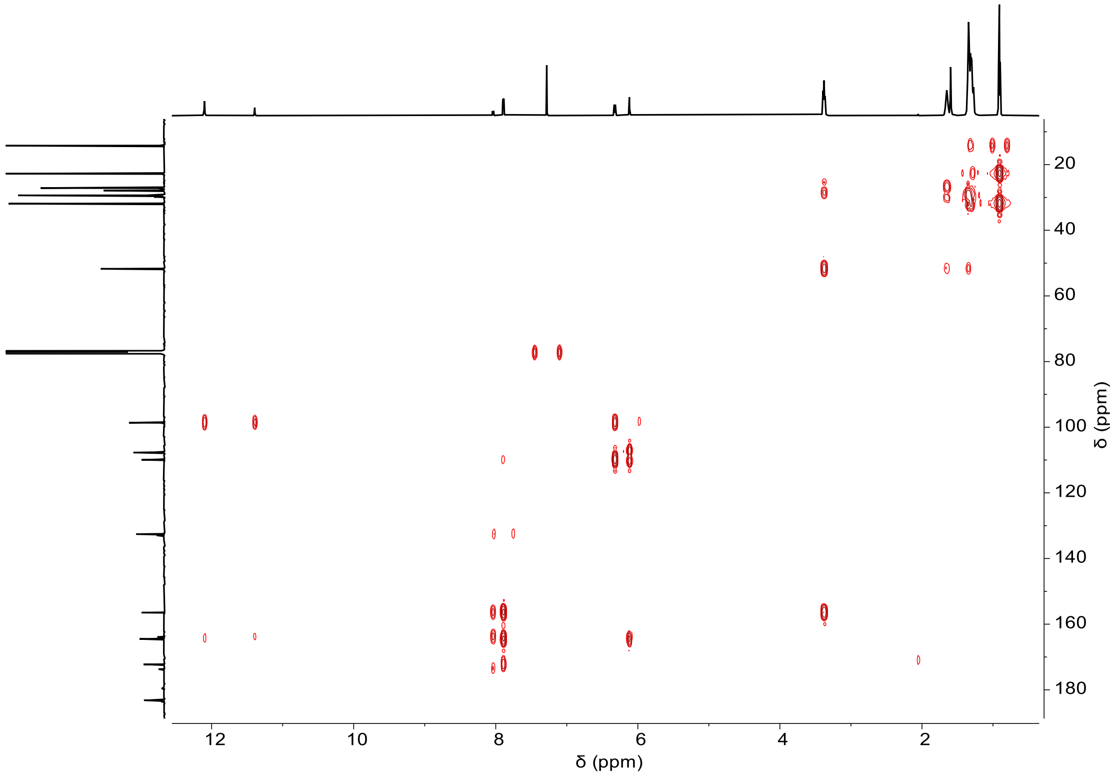

Figure S19. ^{1}H-^{13}C HMBC NMR spectrum (600 MHz, $CDCl_3$) of **SQ4**.

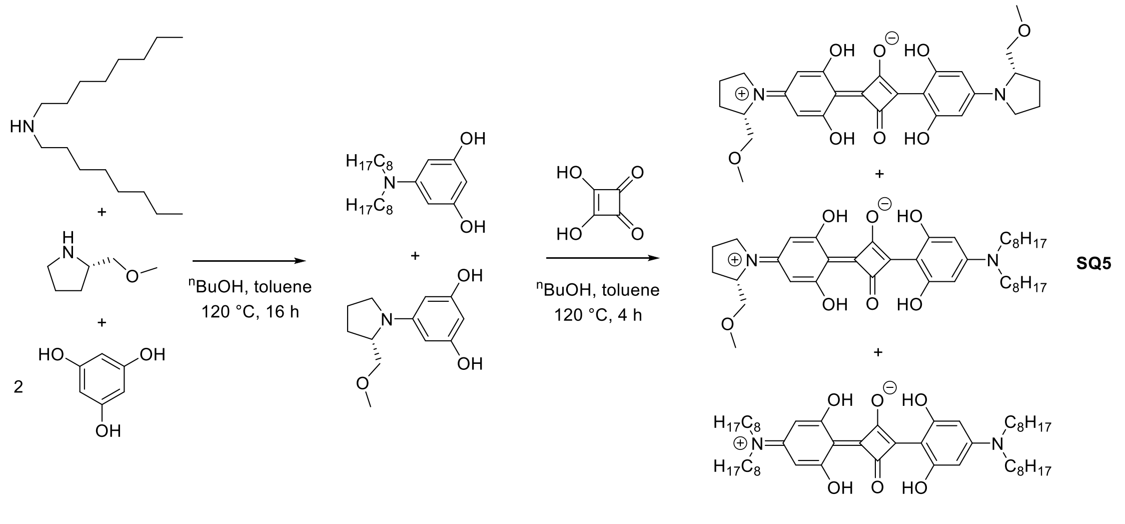

Scheme S5. Synthesis of **SQ5**

(*S*)-(+)-2-(Methoxymethyl)pyrrolidine (302 mg, 2.62 mmol, 1 equiv.), di-*n*-octylamine (0.8 mL, 2.65 mmol, 1 equiv.), and phloroglucinol (660 mg, 5.23 mmol, 2 equiv.) were dissolved in toluene (20 mL) and *n*-butanol (20 mL). The mixture was heated to reflux and stirred overnight, with water removed azeotropically *via* a Dean-Stark setup. The yellow mixture was briefly cooled down to room temperature, and squaric acid (299 mg, 2.62 mmol, 1 equiv.) was added. After reaching reflux again, the mixture quickly turned blue and showed red fluorescence under ambient light. Heating was continued for 4 h. After cooling down, solvents were concentrated under reduced pressure, MeOH (20 mL) was added, and the mixture was left overnight at –20 °C to promote precipitation. The green solid was isolated by filtration, and the three squaraine dyes were separated by column chromatography (hexane/DCM (7:3) to DCM/MeOH (9:1)). **SQ5** was obtained from the second colored fraction as a green shiny solid (207 mg, 0.32 mmol, 12 %). ^{1}H NMR (600 MHz, $CDCl_3$) δ 11.00 (s, 2H), 10.97 (s, 2H), 5.77 (s, 4H), 4.05 (td, *J* = 7.5, 3.5 Hz, 1H), 3.52 (td, *J* = 8.9, 4.4 Hz, 1H), 3.48 (dd, *J* = 9.5, 3.5 Hz, 1H), 3.38 – 3.34 (m, 4H), 3.32 (t, *J* = 7.7 Hz, 4H), 3.29 – 3.26 (m, 1H), 2.14 – 1.96 (m, 4H), 1.67 – 1.60 (m, 4H), 1.37 – 1.22 (m, 20H), 0.89 (t, *J* = 7.0 Hz, 6H) (Figure S20). ^{13}C NMR (151 MHz, $CDCl_3$) δ 181.44, 163.02, 162.74, 161.62, 161.35, 158.19, 156.65, 102.81, 102.69, 94.65, 93.81, 72.31, 59.39, 58.76, 51.84, 48.96, 31.90, 29.47, 29.37, 28.50, 28.16, 27.13, 22.89, 22.76, 14.22 (Figure S21). HRMS (ESI/QTOF) *m*/*z*: $[M + H]^+$ Calcd for $C_{38}H_{55}N_2O_7^+$ 651.4004; Found 651.4000.

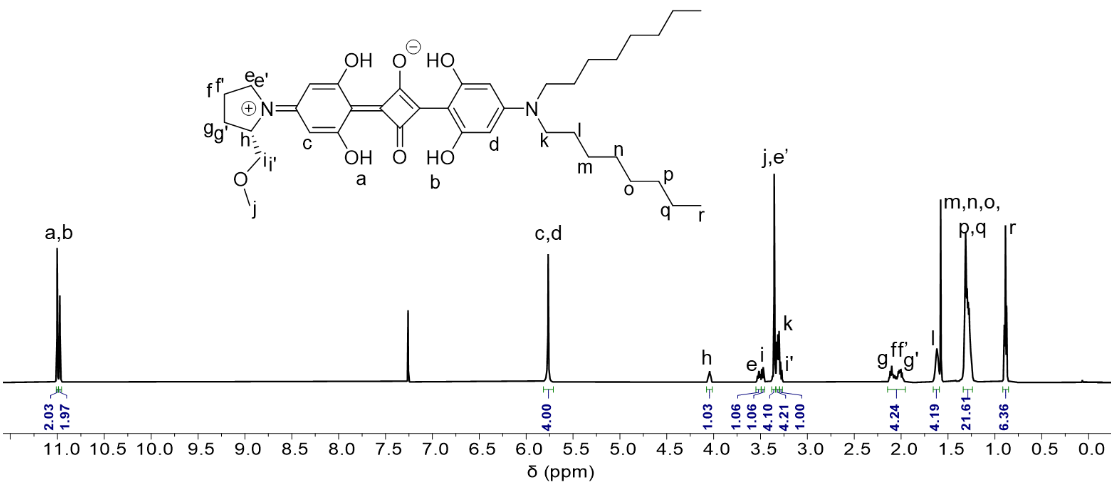

Figure S20. ^{1}H NMR spectrum (600 MHz, $CDCl_3$) of **SQ5**.

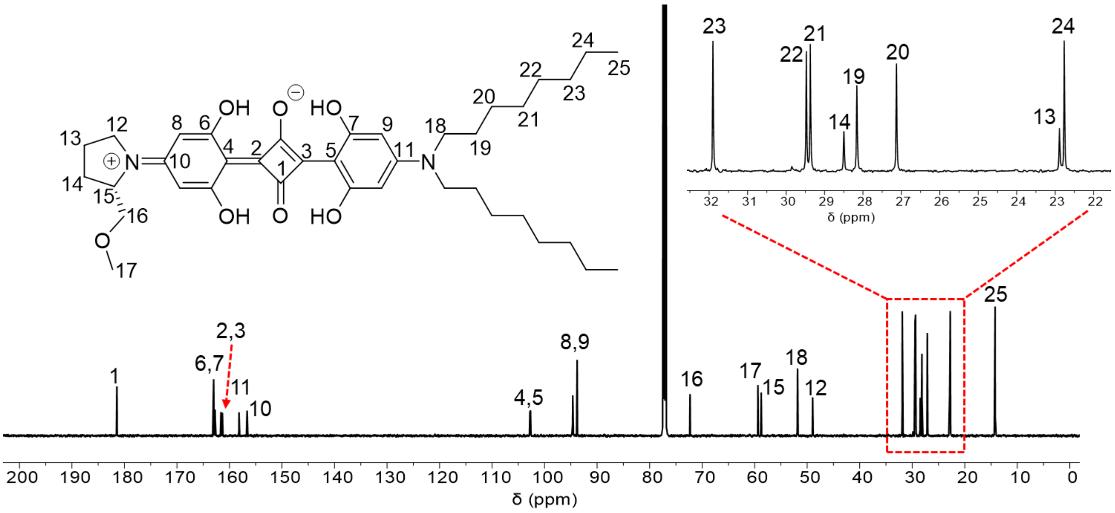

Figure S21. ^{13}C NMR spectrum (151 MHz, $CDCl_3$) of **SQ5**.

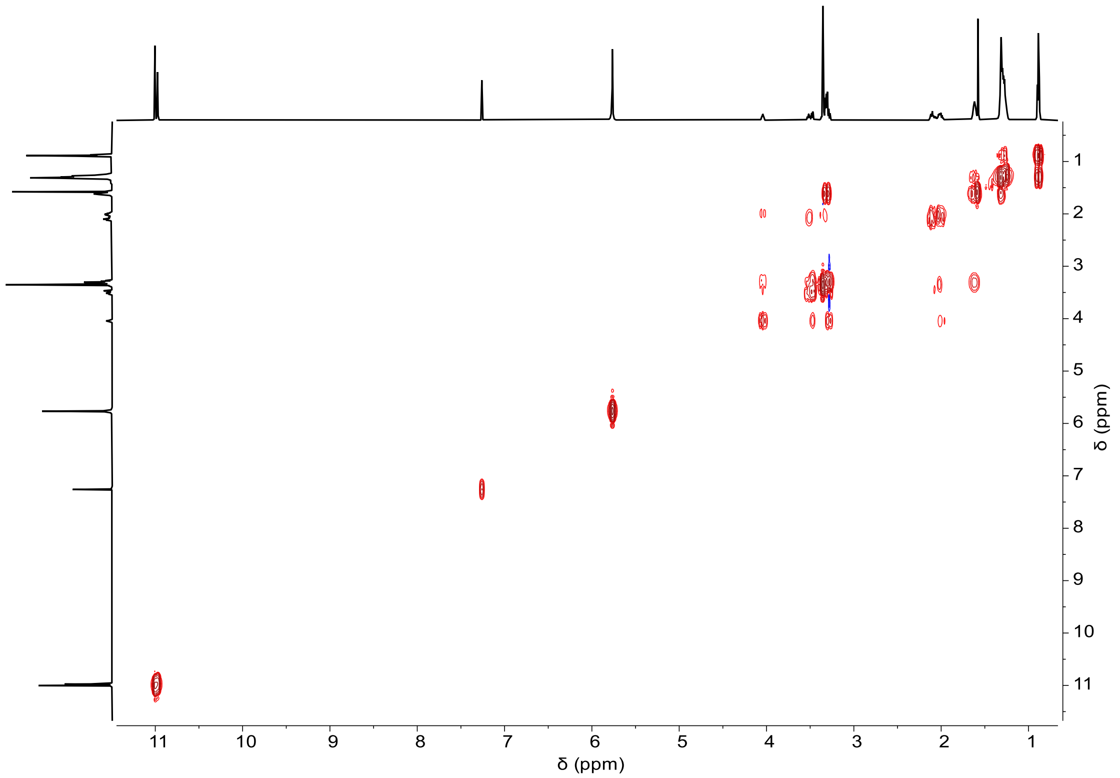

Figure S22. ^{1}H-^{1}H COSY NMR spectrum (400 MHz, $CDCl_3$) of **SQ5**.

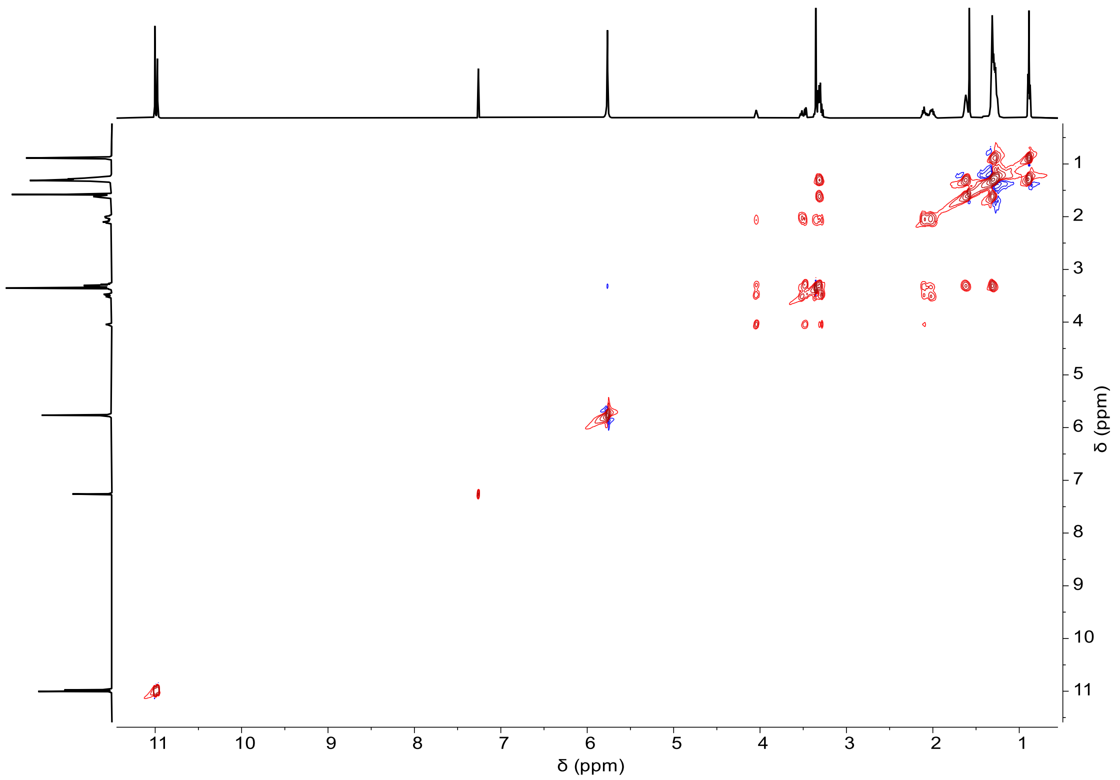

Figure S23. ^{1}H-^{1}H TOCSY NMR spectrum (400 MHz, $CDCl_3$) of **SQ5**.

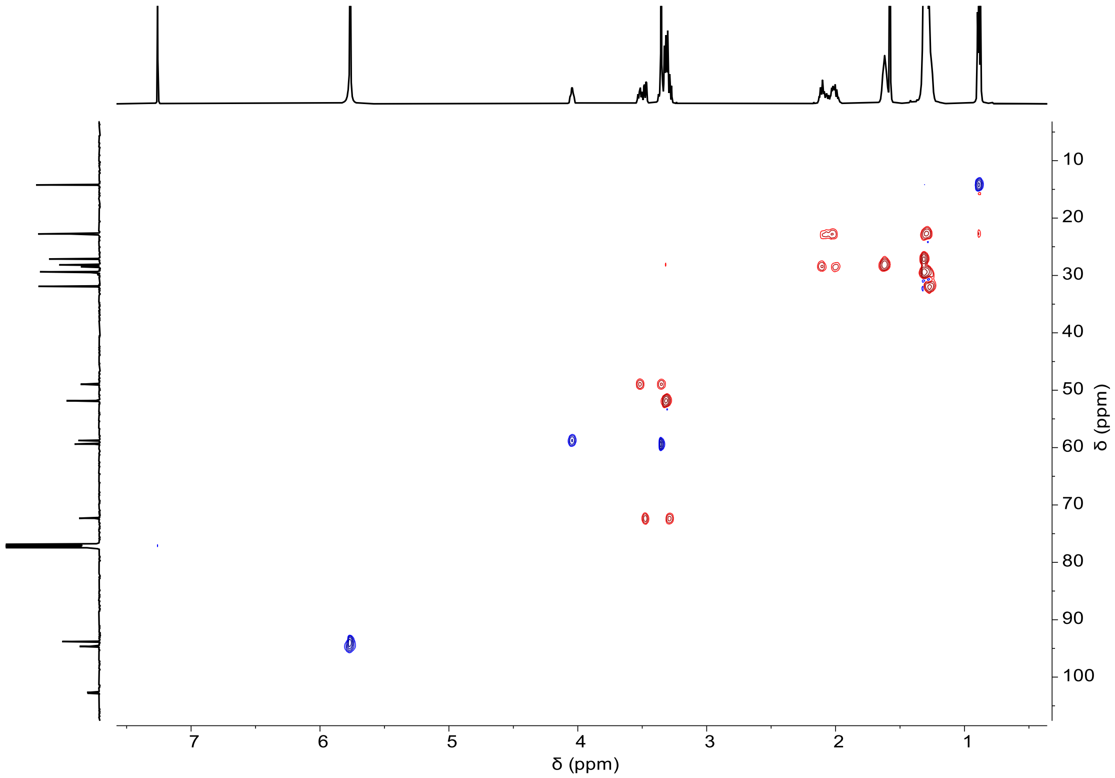

Figure S24. ^{1}H-^{13}C HSQC NMR spectrum (600 MHz, $CDCl_3$) of **SQ5**.

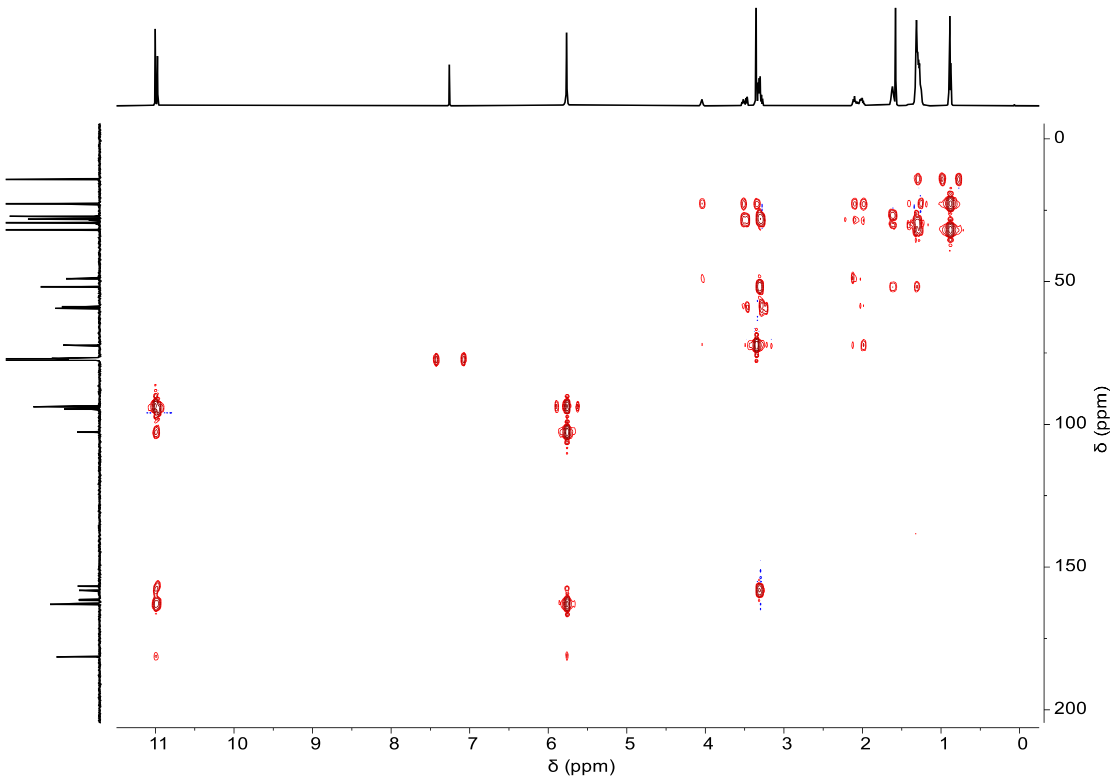

Figure S25. ^{1}H-^{13}C HMBC NMR spectrum (600 MHz, $CDCl_3$) of **SQ5**.

2.2. Cage and host-guest complexes

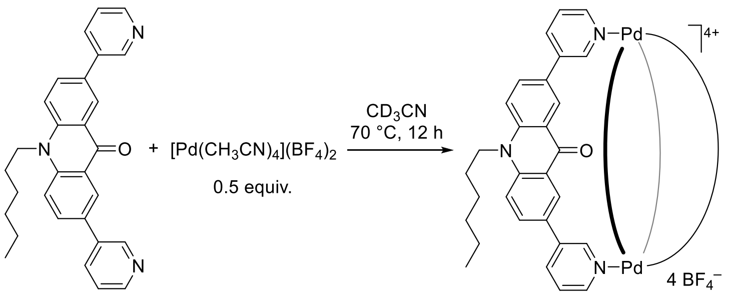

Scheme S6. Synthesis of $[Pd_2(\mathbf{1})_4](BF_4)_4$ (**2**)

To a suspension of **1** (4.00 mg, 9.23 µmol, 4.0 equiv.) in CD_3CN (2 mL) was added 286 µL of a 17.85 mM stock solution of $[Pd(CH_3CN)_4](BF_4)_2$ in CD_3CN (5.10 µmol, 2.2 equiv.). The suspension was stirred overnight at 70 °C.

In most cases, ^1H NMR spectroscopy showed the complete conversion of **1** into **2**, and the obtained solution was used directly without purification. In rare cases, if ^1H NMR spectroscopy showed the presence of impurities, the solution was concentrated under reduced pressure, and added dropwise into diethyl ether (50 mL). The precipitate was isolated by centrifugation, washed with diethyl ether, and dried under vacuum, yielding **2** as a yellow powder.

^1H NMR (400 MHz, CD_3CN) δ 9.73 (d, J = 2.1 Hz, 8H), 9.21 (d, J = 5.7 Hz, 8H), 9.11 (d, J = 2.5 Hz, 8H), 8.32 (d, J = 8.1 Hz, 8H), 8.01 (dd, J = 9.0, 2.5 Hz, 8H), 7.79 (d, J = 9.1 Hz, 8H), 7.72 (dd, J = 8.1, 5.7 Hz, 8H), 4.37 (t, J = 8.1 Hz, 8H), 1.84 – 1.71 (m, 8H), 1.55 – 1.43 (m, 8H), 1.36 – 1.21 (m, 16H), 0.84 (t, J = 6.9 Hz, 12H) (Figure S28).

^{13}C NMR (151 MHz, CD_3CN) δ 177.87, 150.07, 149.95, 143.29, 139.70, 138.82, 133.55, 128.87, 128.34, 126.44, 123.63, 47.19, 32.08, 27.84, 26.81, 23.20, 14.16 (Figure S29).

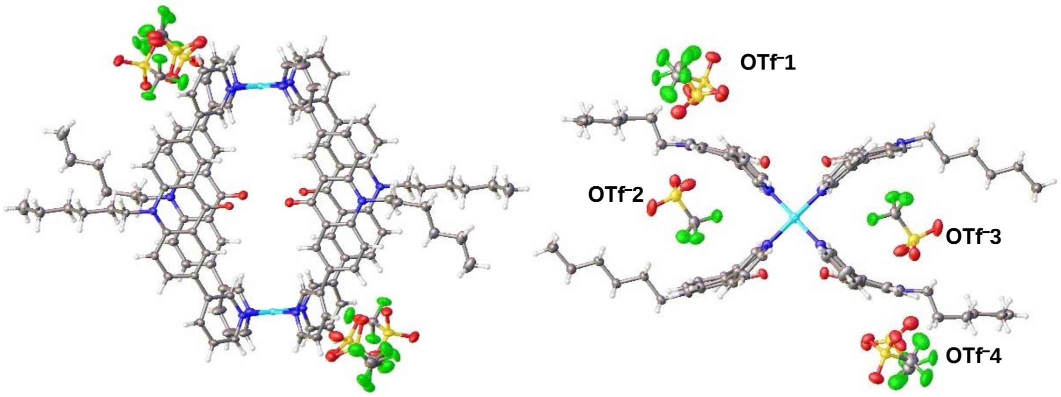

Figure S26. Solid-state structure of **2** as determined by single-crystal XRD, with thermal ellipsoids drawn at the 30% probability level. C: grey, N: blue, O: red, H: white, F: green, S: yellow, and Pd: cyan. OTf^-1 and OTf^-4 are disordered over two positions.

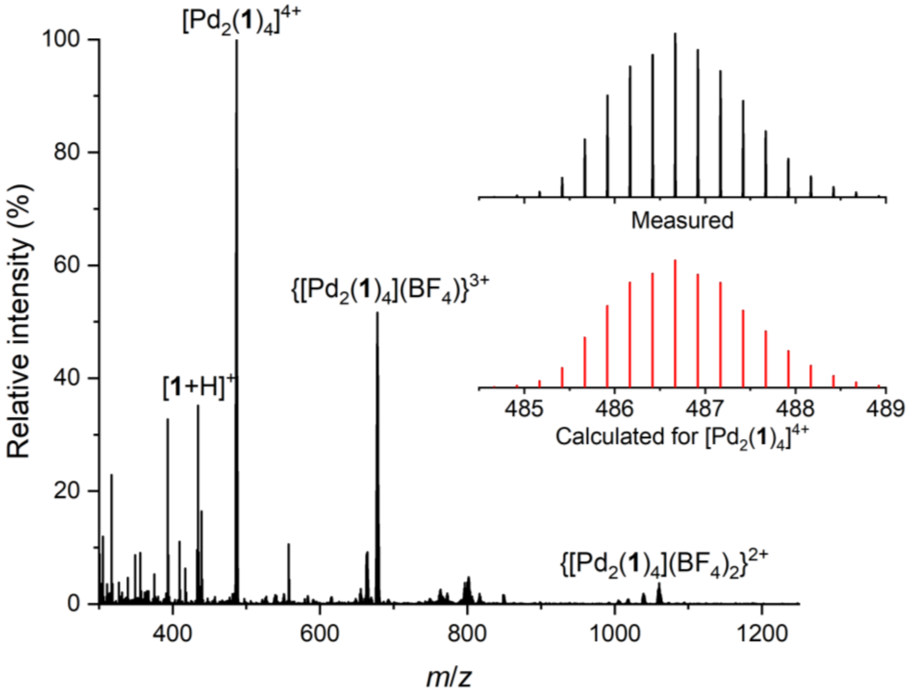

Figure S27. High-Resolution ESI mass spectrum of **2**.

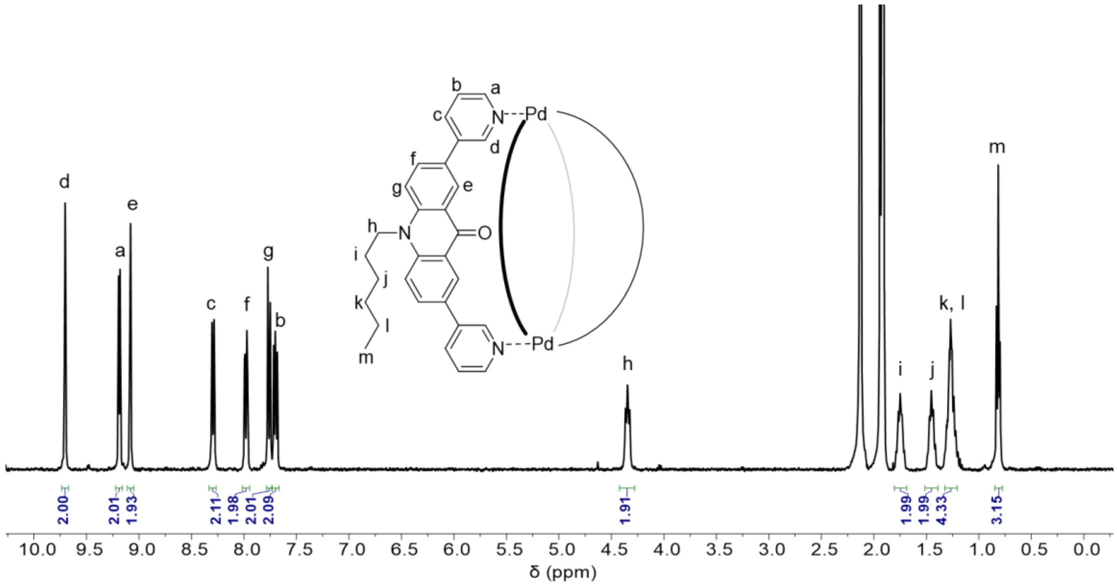

Figure S28. ^{1}H NMR spectrum (400 MHz, CD_3CN) of **2**.

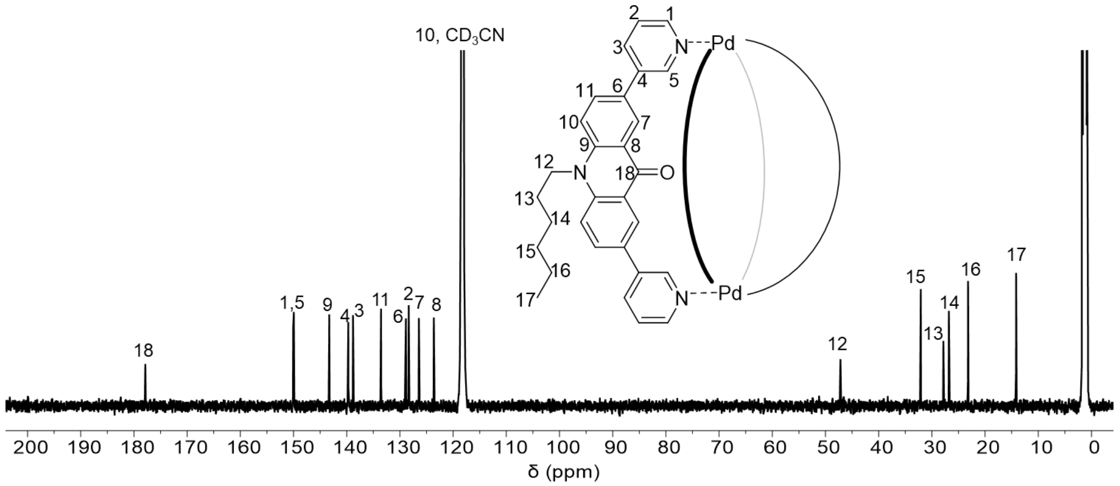

Figure. S29. ^{13}C NMR spectrum (151 MHz, CD_3CN) of **2**.

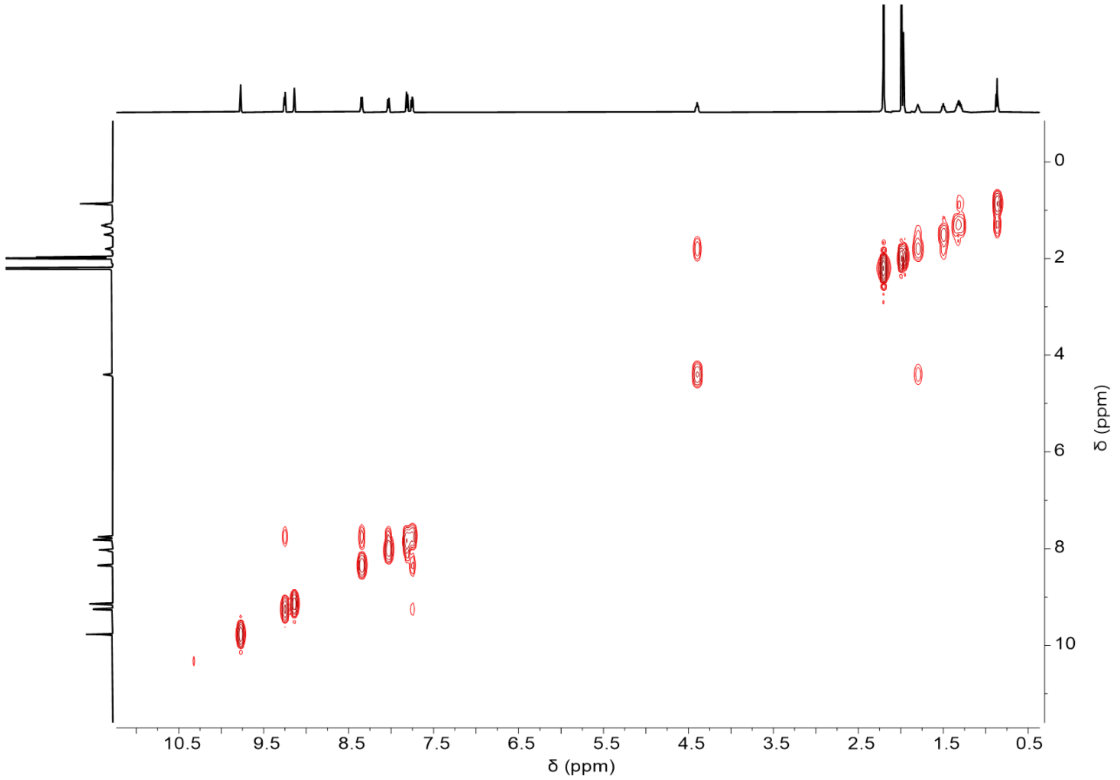

Figure S30. ^{1}H-^{1}H COSY NMR spectrum (600 MHz, CD_3CN) of **2**.

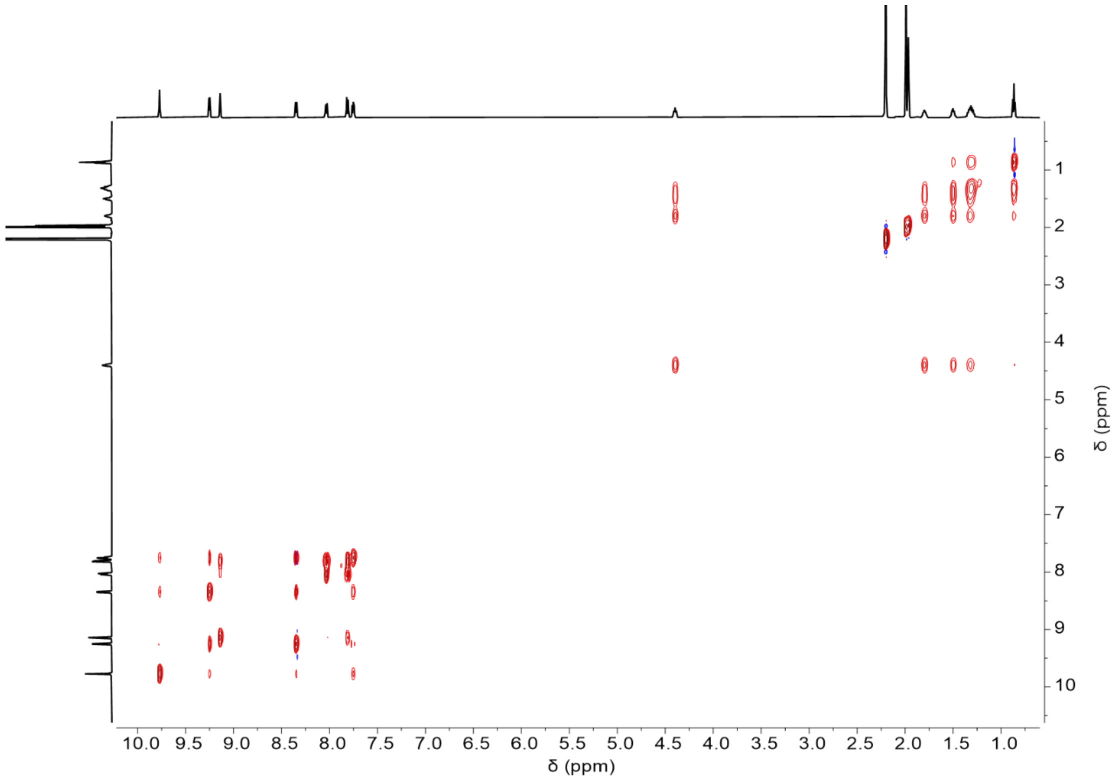

Figure S31. ^{1}H-^{1}H TOCSY NMR spectrum (600 MHz, CD_3CN) of **2**.

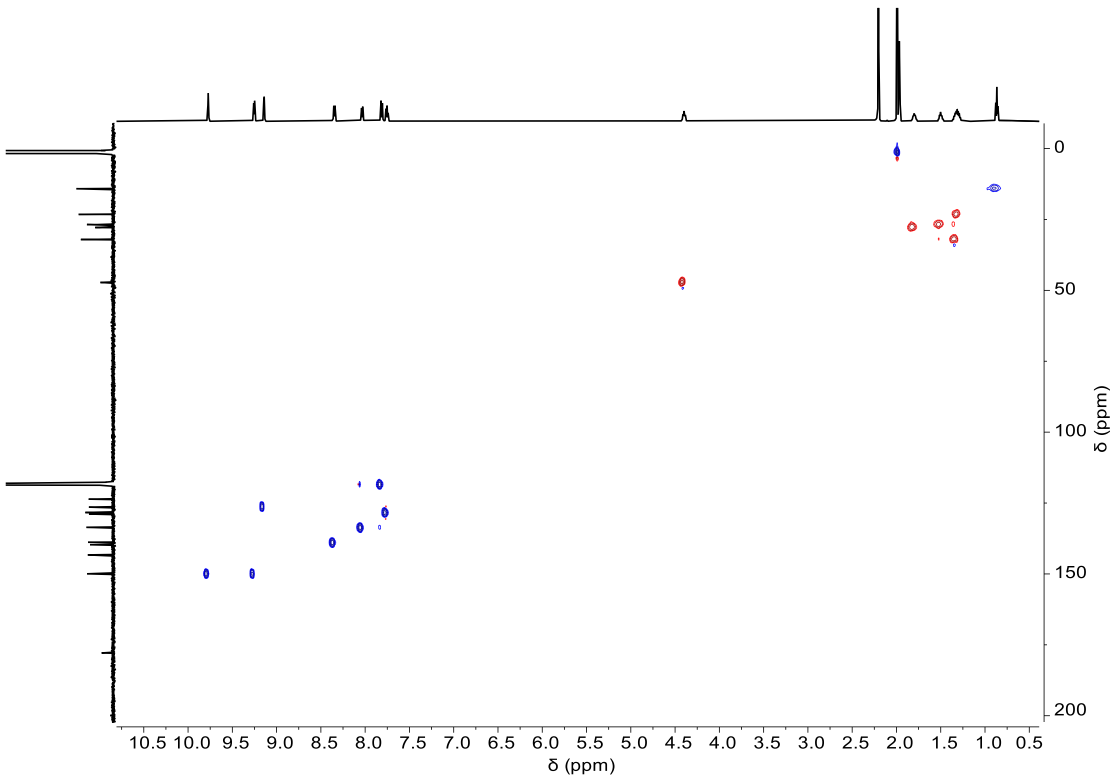

Figure S32. ^{1}H-^{13}C HSQC NMR spectrum (600 MHz, CD_3CN) of **2**.

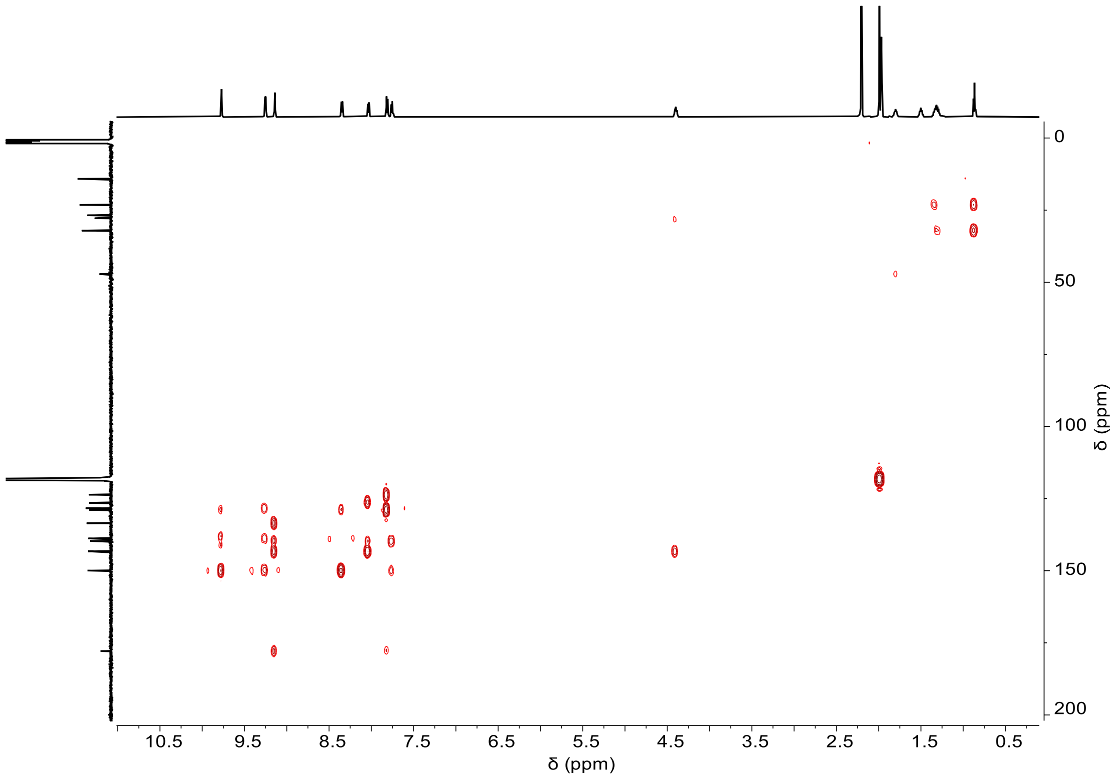

Figure S33. ^1H-^{13}C HMBC NMR spectrum (600 MHz, CD_3CN) of **2**.

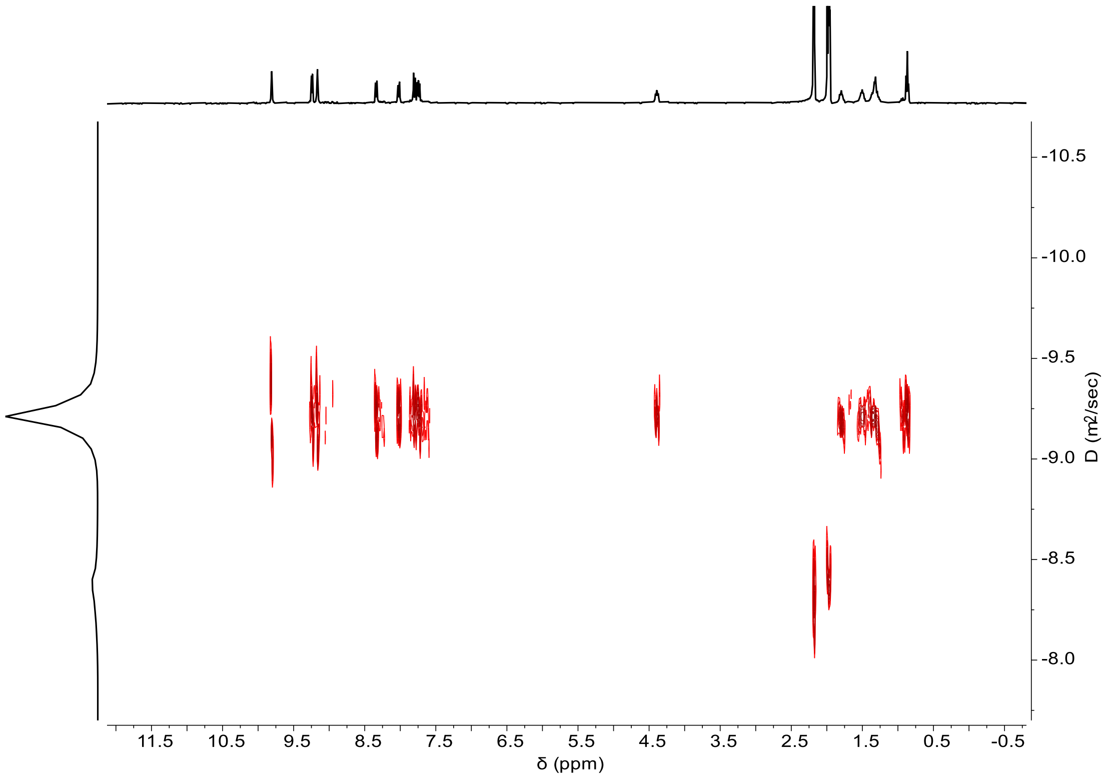

Figure S34. ^1H DOSY NMR spectrum (400 MHz, CD_3CN) of **2**, D = –9.20 m^2/s.

General procedure for the synthesis of **SQ⊂2** (**SQ** = **SQ1**, **SQ2**, **SQ4**, **SQ5**)

A slight excess (1.2 equiv.) of the corresponding squaraine dye was added to a CD_3CN solution of **2**. The suspension was stirred overnight at 70 °C. ^1H NMR spectroscopy was used to confirm that the empty cage **2** was no longer present. The resulting suspension was filtered through a PTFE hydrophilic syringe filter, yielding the host-guest complex as a dark green solution, which was used directly without further purification.

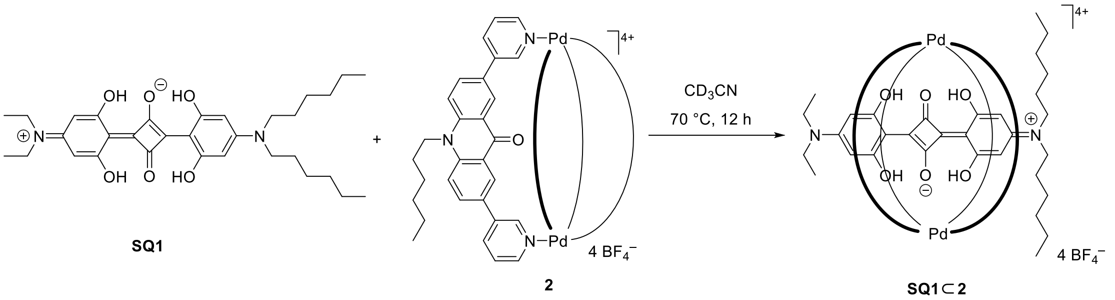

Scheme S7. Synthesis of **SQ1⊂2**.

^1H NMR (600 MHz, CD_3CN) δ 13.61 (s, 2H), 10.30 (d, *J* = 2.1 Hz, 4H), 9.91 (d, *J* = 1.9 Hz, 4H), 9.48 (dd, *J* = 5.9, 1.2 Hz, 4H), 9.37 (dd, *J* = 5.9, 1.3 Hz, 4H), 8.98 (d, *J* = 2.5 Hz, 4H), 8.94 (d, *J* = 2.5 Hz, 4H), 8.85 (s, 2H), 8.39 (d, *J* = 8.0 Hz, 4H), 8.32 (d, *J* = 8.1 Hz, 4H), 8.00 (dd, *J* = 9.0, 2.5 Hz, 4H), 7.87 (dd, *J* = 8.9, 2.5 Hz, 4H), 7.80 (dd, *J* = 8.0, 5.9 Hz, 4H), 7.76 (dd, *J* = 7.9, 5.9 Hz, 4H), 7.67 (d, *J* = 8.9 Hz, 4H), 7.48 (d, *J* = 8.8 Hz, 4H), 4.67 (s, 2H), 4.46 (s, 2H), 4.26 (t, *J* = 8.4 Hz, 4H), 3.88 (t, *J* = 7.5 Hz, 4H), 3.25 – 3.14 (m, 4H), 1.82 – 1.75 (m, 4H), 1.67 – 1.59 (m, 4H), 1.53 (p, *J* = 7.6 Hz, 4H), 1.45 – 1.40 (m, 4H), 1.40 – 1.22 (m, 20H), 1.11 (t, *J* = 5.8 Hz, 6H), 0.90 (t, *J* = 7.4 Hz, 12H), 0.57 – 0.47 (m, 8H), 0.17 – 0.11 (m, 4H), –0.10 (t, *J* = 7.3 Hz, 6H), –0.28 – –0.36 (m, 4H) (Figure S37).

^{13}C NMR (151 MHz, CD_3CN) δ 184.84, 177.48, 176.05, 163.59, 162.09, 161.75, 158.00, 156.98, 156.45, 150.05, 149.80, 143.44, 142.97, 140.69, 139.96, 138.85, 138.49, 133.59, 132.91, 128.77, 128.70, 128.24, 127.47, 126.86, 123.48, 123.41, 117.85, 95.84, 91.77, 50.70, 45.77, 32.08, 31.94, 30.99, 27.91, 26.81, 26.76, 26.51, 26.13, 23.34, 23.20, 23.15, 14.21, 14.18, 13.87, 13.65 (Figure S38).

.

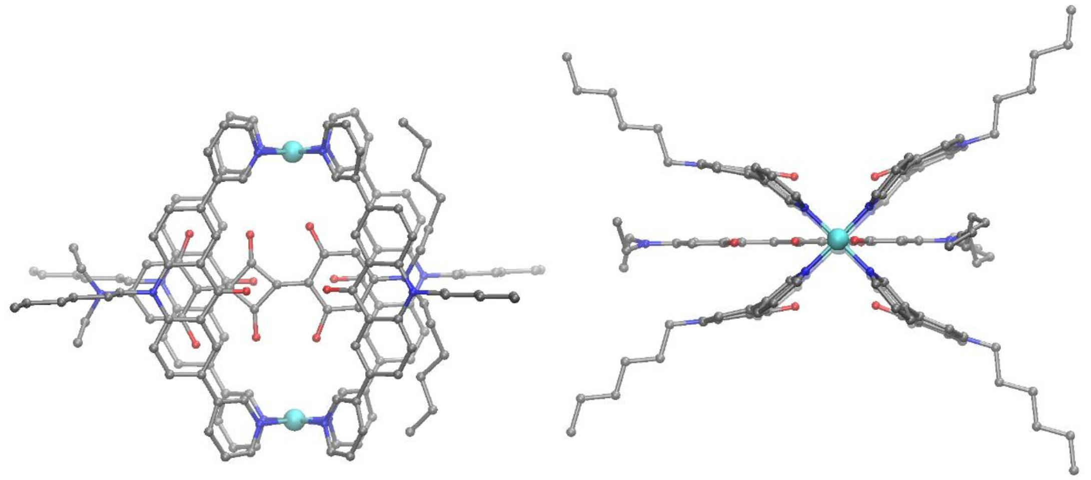

Figure S35. xTB-optimized structure of **SQ1**⊂**2**, viewed from the side and from the Pd-Pd axis. Hydrogens are omitted for clarity. C: gray, N: blue, O: red, Pd: cyan. For details, see Section 6.

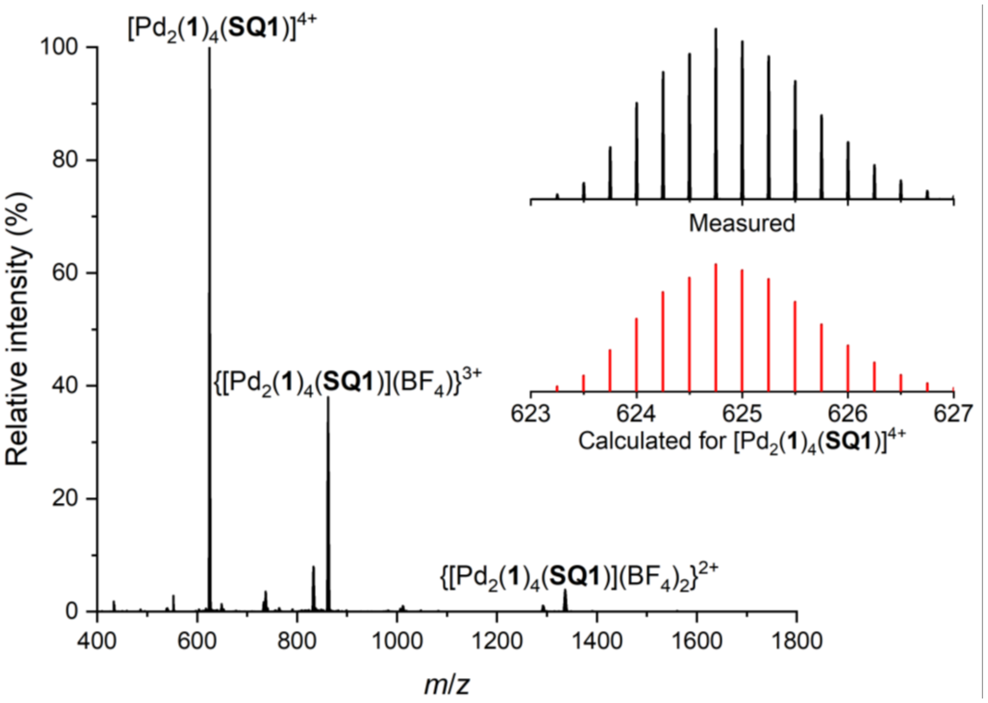

Figure S36. High-Resolution ESI mass spectrum of **SQ1**⊂**2**.

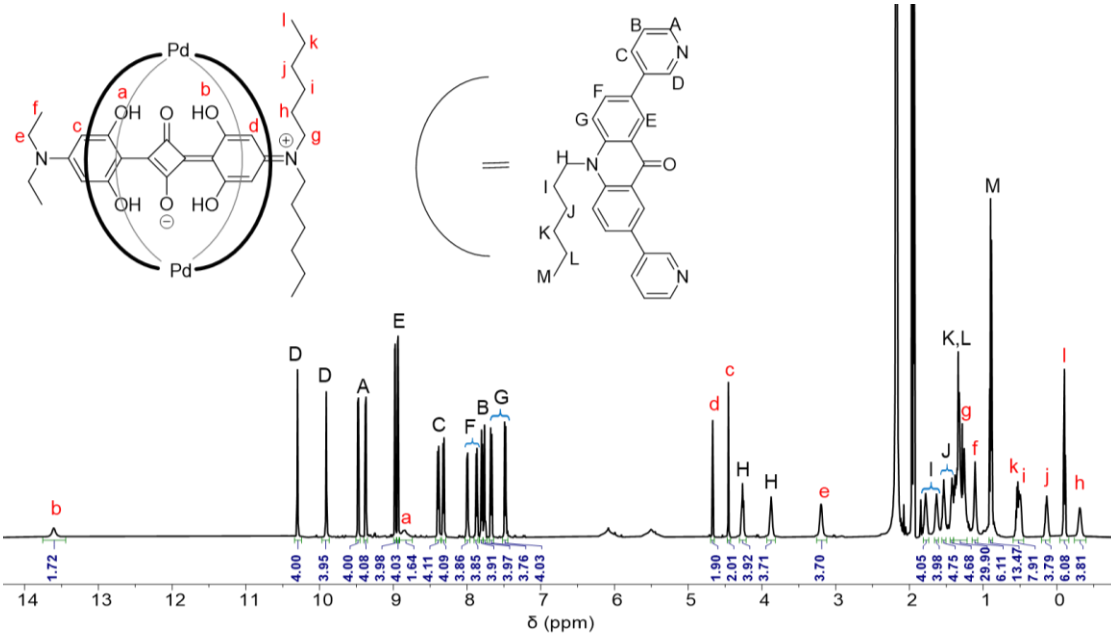

Figure S37. ^{1}H NMR spectrum (600 MHz, CD_3CN) of **SQ1⊂2**. Protons A-J of the cage are split into two sets of signals, showing that the encapsulation of **SQ1** resulted in a lower symmetry, in this case $D_{4h} \rightarrow C_{2v}$. Alternating protons (h, j, l) on the long arm of **SQ1** are especially shielded (–0.31 to 0.14 ppm), possibly due to C–H --- π interactions with the ligand's acridone rings, also observed in the xTB-optimized structure.

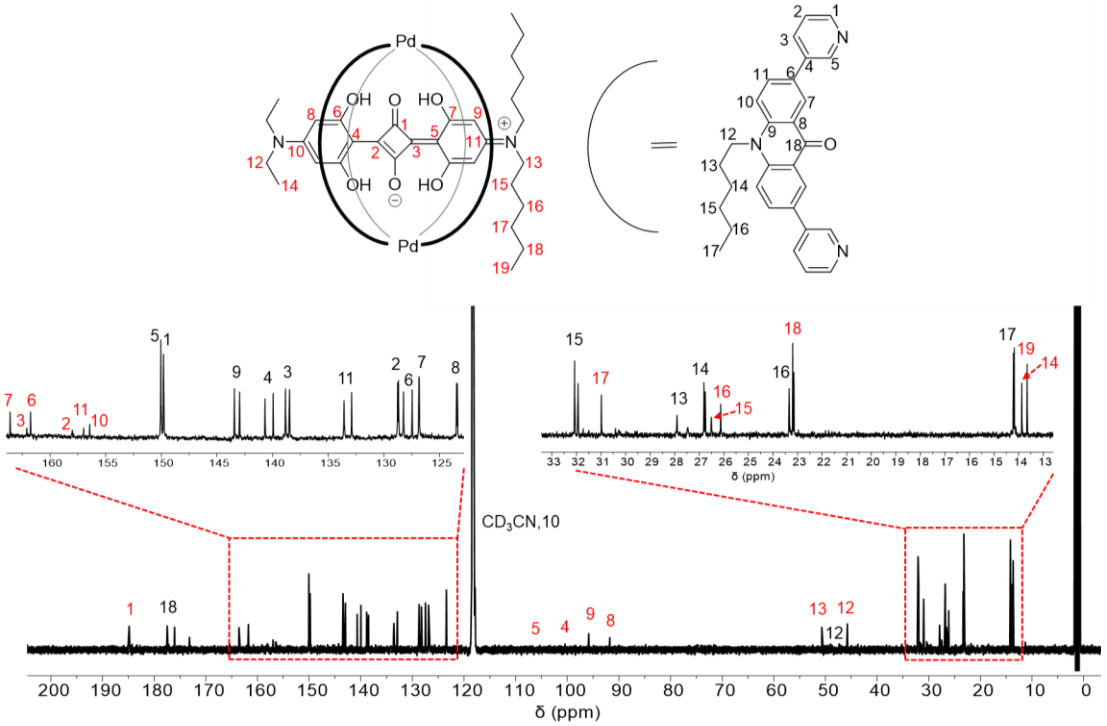

Figure S38. ^{13}C NMR spectrum (600 MHz, CD_3CN) of **SQ1⊂2**.

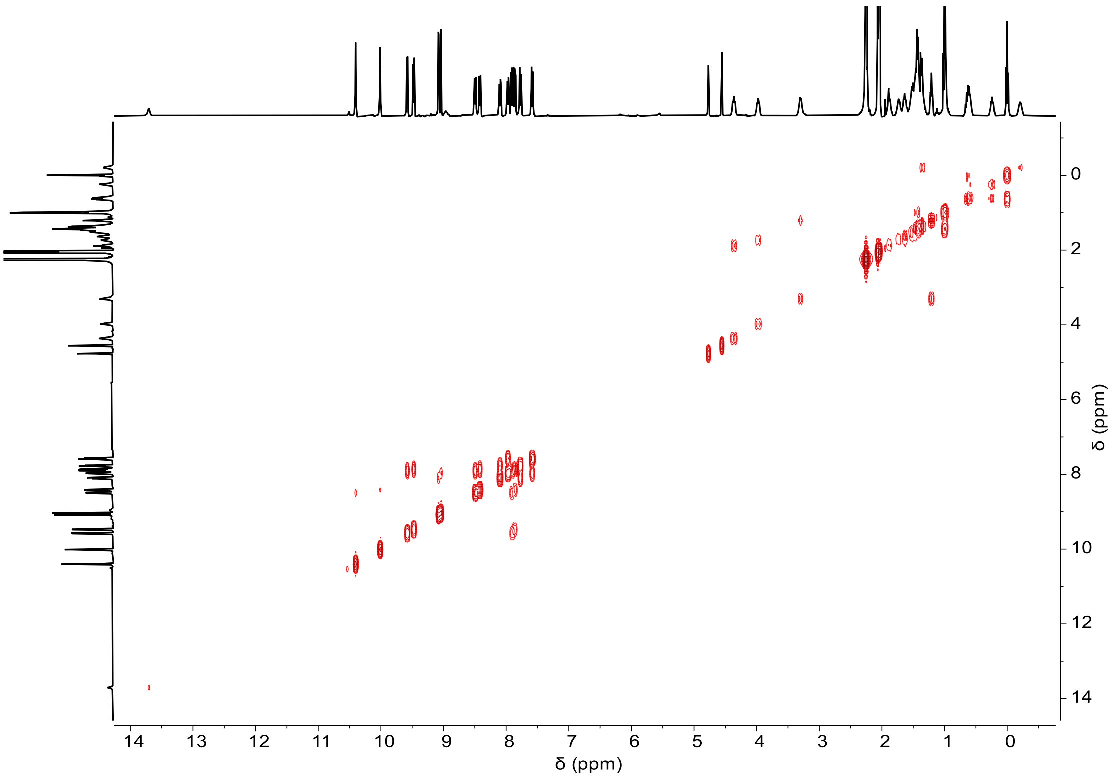

Figure S39. ^{1}H-^{1}H COSY NMR spectrum (400 MHz, CD_3CN) of **SQ1⊂2**.

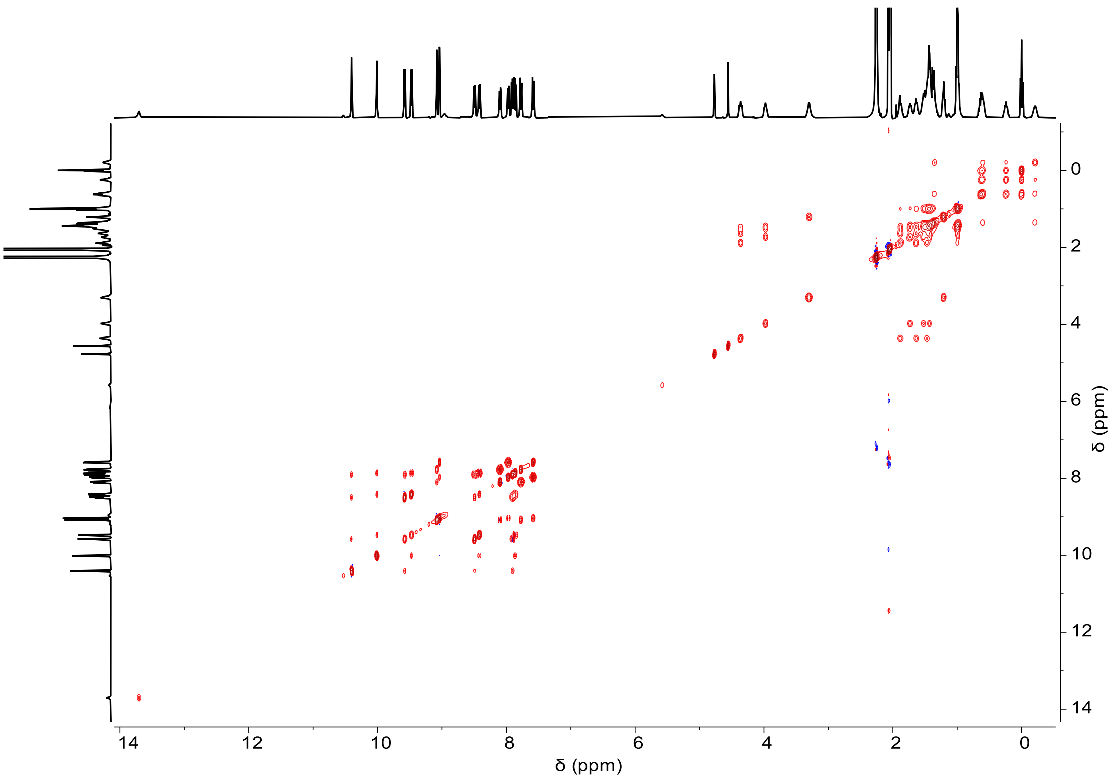

Figure S40. ^{1}H-^{1}H TOCSY NMR spectrum (400 MHz, CD_3CN) of **SQ1⊂2**.

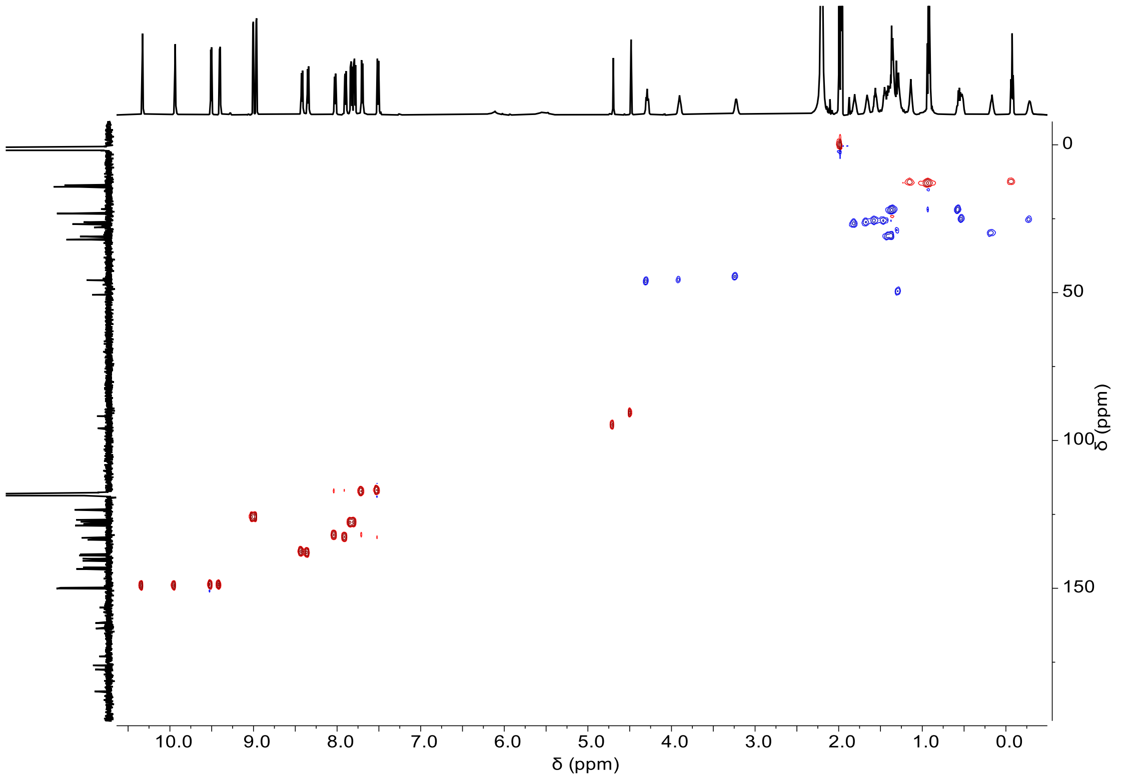

Figure S41. ^{1}H-^{13}C HSQC NMR spectrum (600 MHz, CD_3CN) of **SQ1⊂2**.

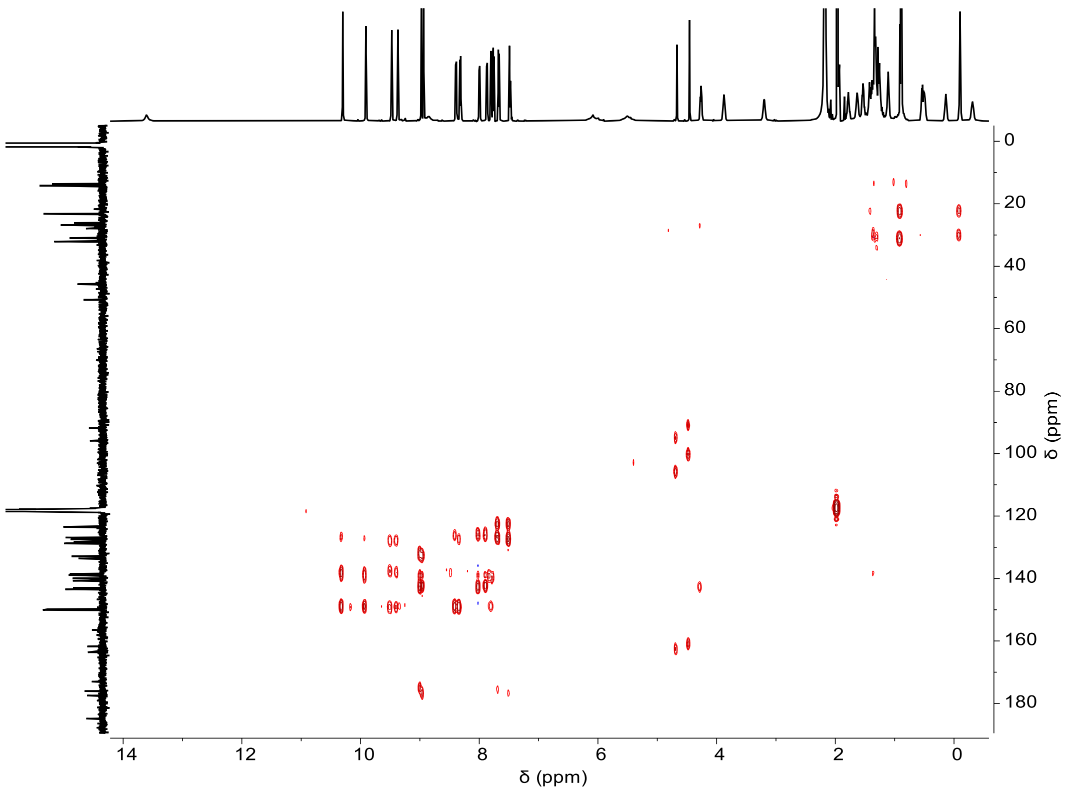

Figure S42. ^{1}H-^{13}C HMBC NMR spectrum (600 MHz, CD_3CN) of **SQ1⊂2**.

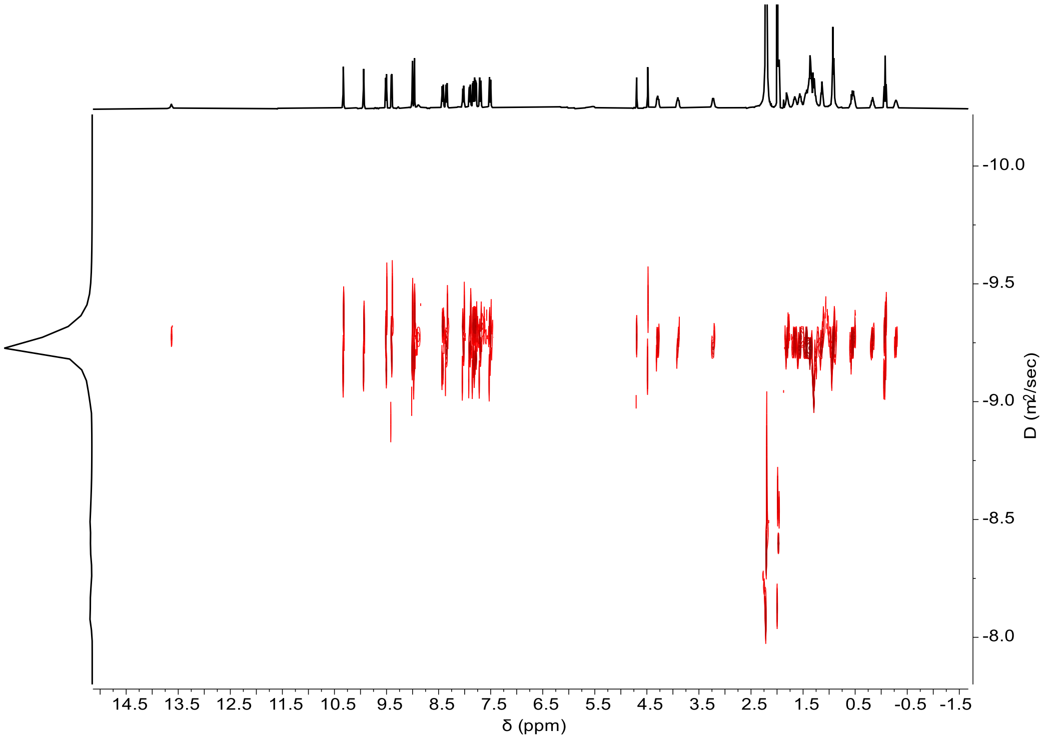

Figure S43. ^{1}H DOSY NMR spectrum (400 MHz, CD_3CN) of **SQ1⊂2**, $D = -9.22$ m^2/s.

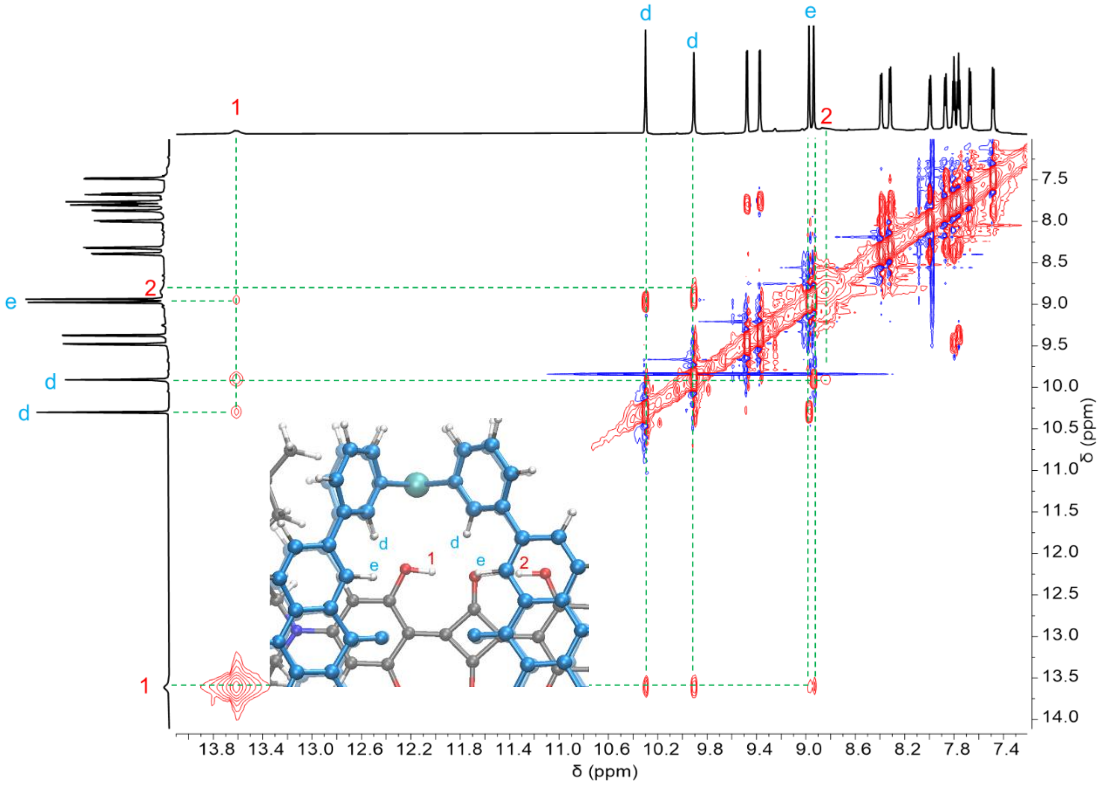

Figure S44. ^{1}H-^{1}H NOESY NMR spectrum (800 MHz, CD_3CN) of **SQ1⊂2**. Insert: binding motif illustrated with the xTB-optimized structure. Green dotted lines highlight cross peaks between OH protons of **SQ1** (H_1 and H_2) and inward-pointing protons of **2** (H_d and H_e). The centered H_1 is close to all 8 H_d protons (in 2 environments), while the off-centered H_2 is only close to 4 of the H_d protons.

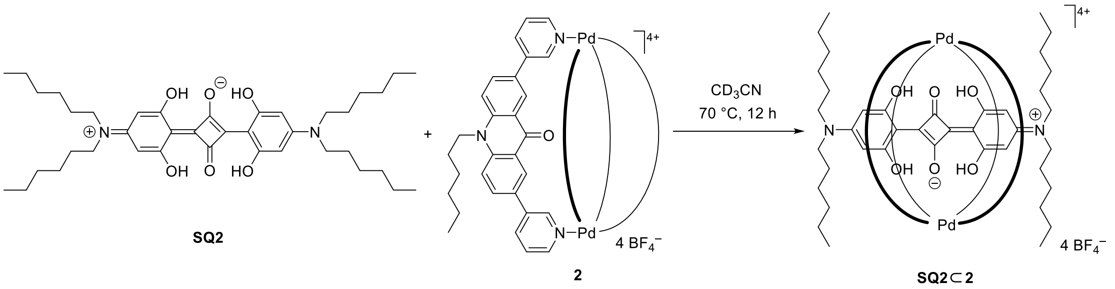

Scheme S8. Synthesis of **SQ2⊂2**.

The NMR characterization was performed at 345 K because **SQ2** shuttles between two degenerate binding positions in **2**, and the exchange is on fast to intermediate timescale at R.T., causing many signals to be invisible. For details, see variable temperature NMR studies (Figure S49 and S50).

^{1}H NMR (345 K, 800 MHz, CD_3CN) δ 11.24 (br. s, 4H), 10.13 (d, *J* = 2.5 Hz, 8H), 9.52 – 9.40 (m, 8H), 9.01 (d, *J* = 2.5 Hz, 8H), 8.38 (d, *J* = 7.8 Hz, 8H), 7.95 (dd, *J* = 8.9, 2.5 Hz, 8H), 7.80 (dd, *J* = 7.8, 6.0 Hz, 8H), 7.57 (d, *J* = 8.5 Hz, 8H), 4.58 (s, 4H), 4.10 (t, *J* = 6.9 Hz, 8H), 2.21 (br. s, 8H), 1.86 – 1.71 (m, 8H), 1.51 (p, *J* = 7.3 Hz, 8H), 1.45 – 1.25 (m, 16H), 1.05 – 0.99 (m, 8H), 0.97 – 0.88 (m, 20H), 0.85 (br. s, 8H), 0.65 (br. s, 8H), 0.54 (t, *J* = 7.4 Hz, 12H) (Figure S48).

^{13}C NMR (345 K, 151 MHz, CD_3CN) δ 185.43, 177.11, 163.30, 161.09, 157.60, 150.50, 150.36, 143.85, 140.93, 139.14, 133.61, 129.20, 128.31, 127.44, 124.15, 104.67, 94.55, 51.86, 47.61, 32.47, 32.08, 28.32, 28.26, 27.29, 27.10, 23.61, 23.59, 14.46, 14.32 (Figure S52).

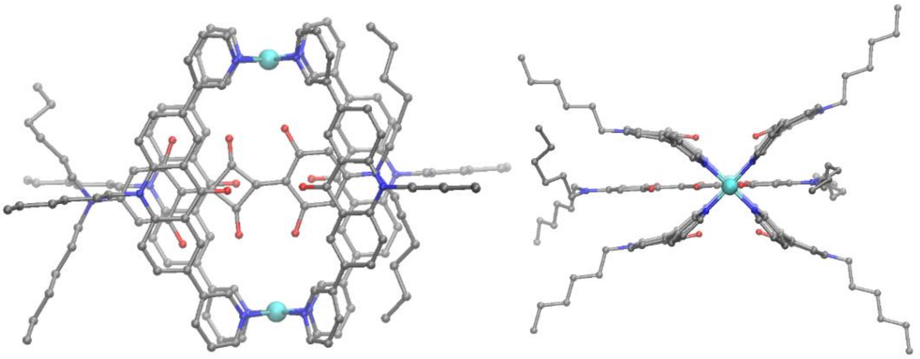

Figure S45. xTB-optimized structure of **SQ2⊂2**, viewed from the side and from the Pd-Pd axis. Hydrogens are omitted for clarity. C: gray, N: blue, O: red, Pd: cyan. For details, see Section 6.

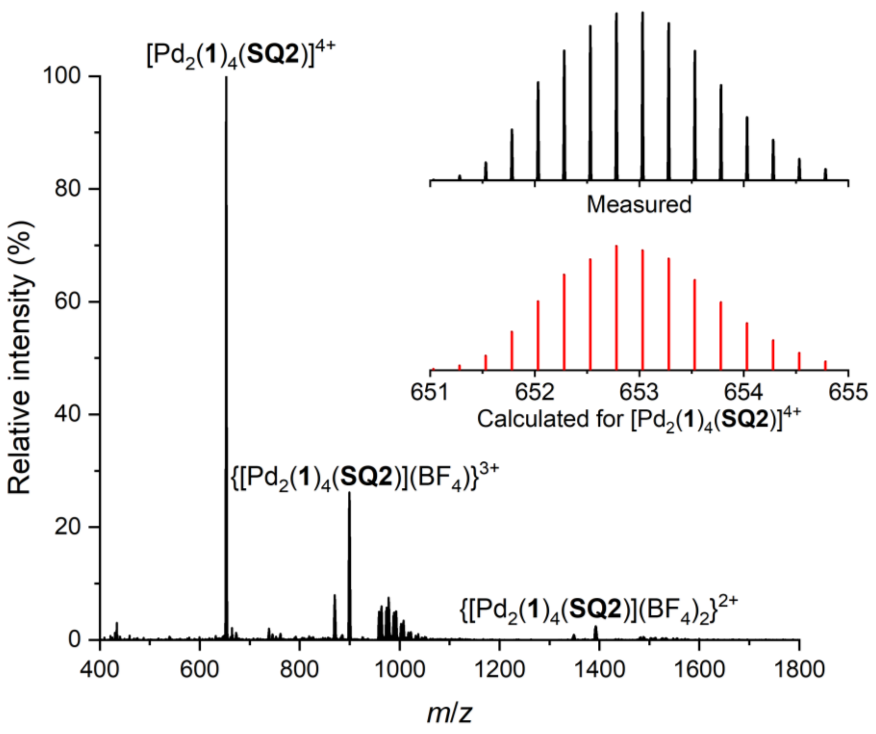

Figure S46. High-Resolution ESI mass spectrum of **SQ2⊂2**.

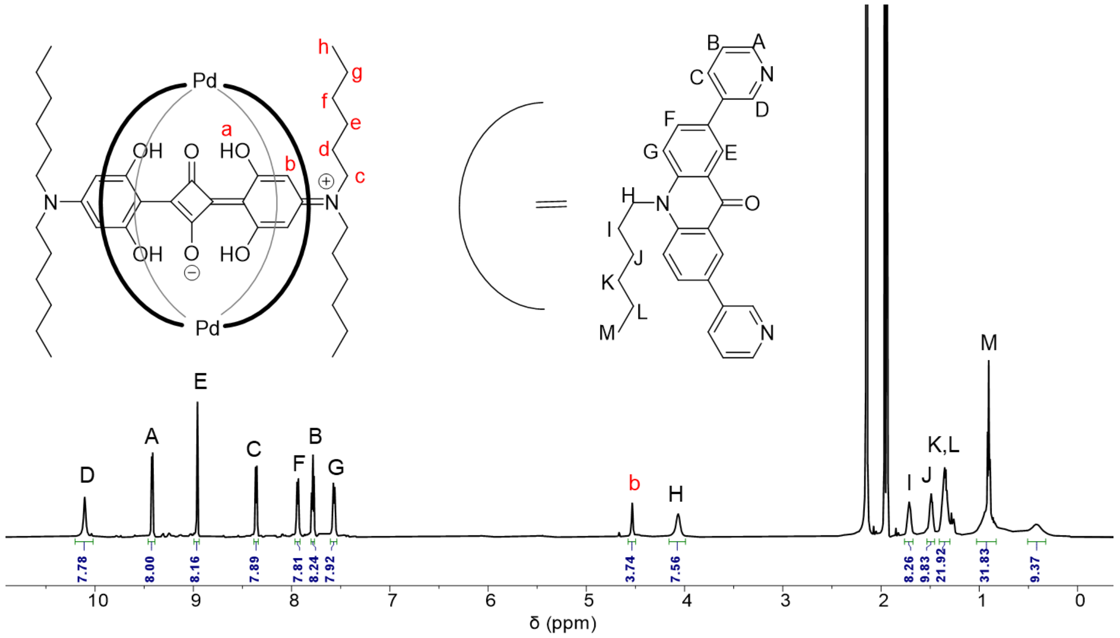

Figure S47. ^{1}H NMR spectrum (298 K, 600 MHz, CD_3CN) of **SQ2⊂2**. As explained above, due to the dye shuttling between two positions with an intermediate exchange rate at R.T., the dye's OH protons are not visible, and the dye's alkyl protons appear as broad signals in the 0.4–2 ppm region.

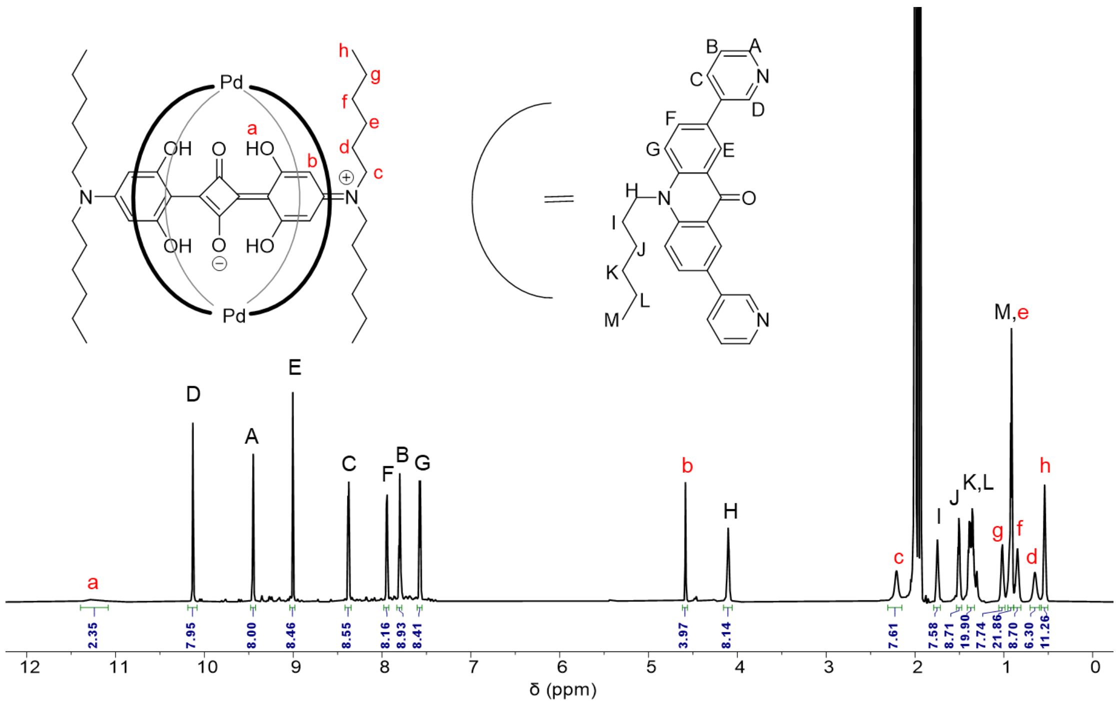

Figure S48. ^{1}H NMR spectrum (345 K, 800 MHz, CD_3CN) of **SQ2⊂2**. At this temperature, the shuttling becomes fast on the NMR timescale, and as a result all protons are visible, with a high apparent symmetry.

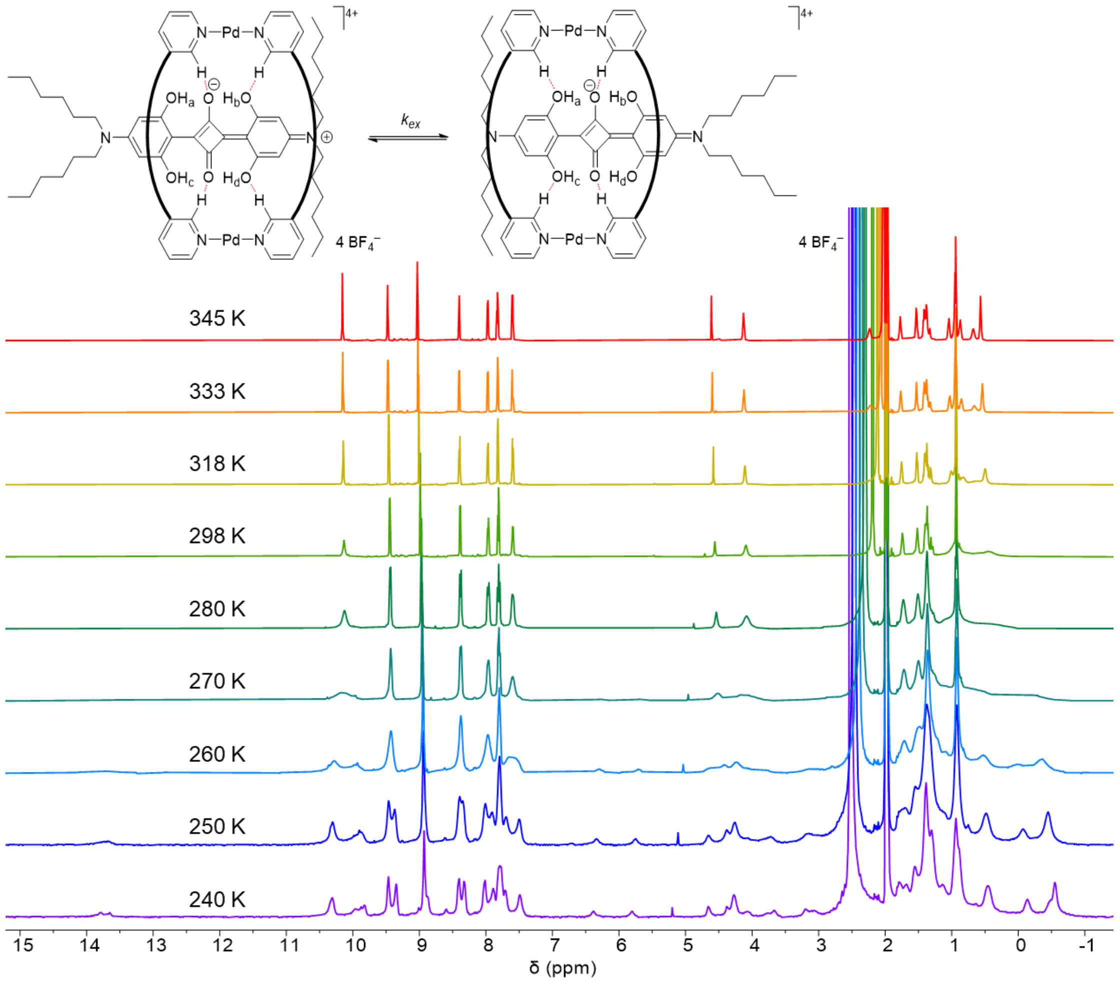

Figure S49. ^{1}H NMR spectra (CD$_3$CN, 345 K–298 K: 800 MHz, 280 K–240 K: 400 MHz) of **SQ2⊂2**. The OH protons and alkyl protons of **SQ2** are in intermediate to fast exchange regime at R.T., and appear broad or invisible. At 318 K or above, their exchange rates become fast on ^{1}H NMR timescale, and they appear as one set of signals; at 260 K or below, their exchange rates become slow, and they appear as two sets of signals.

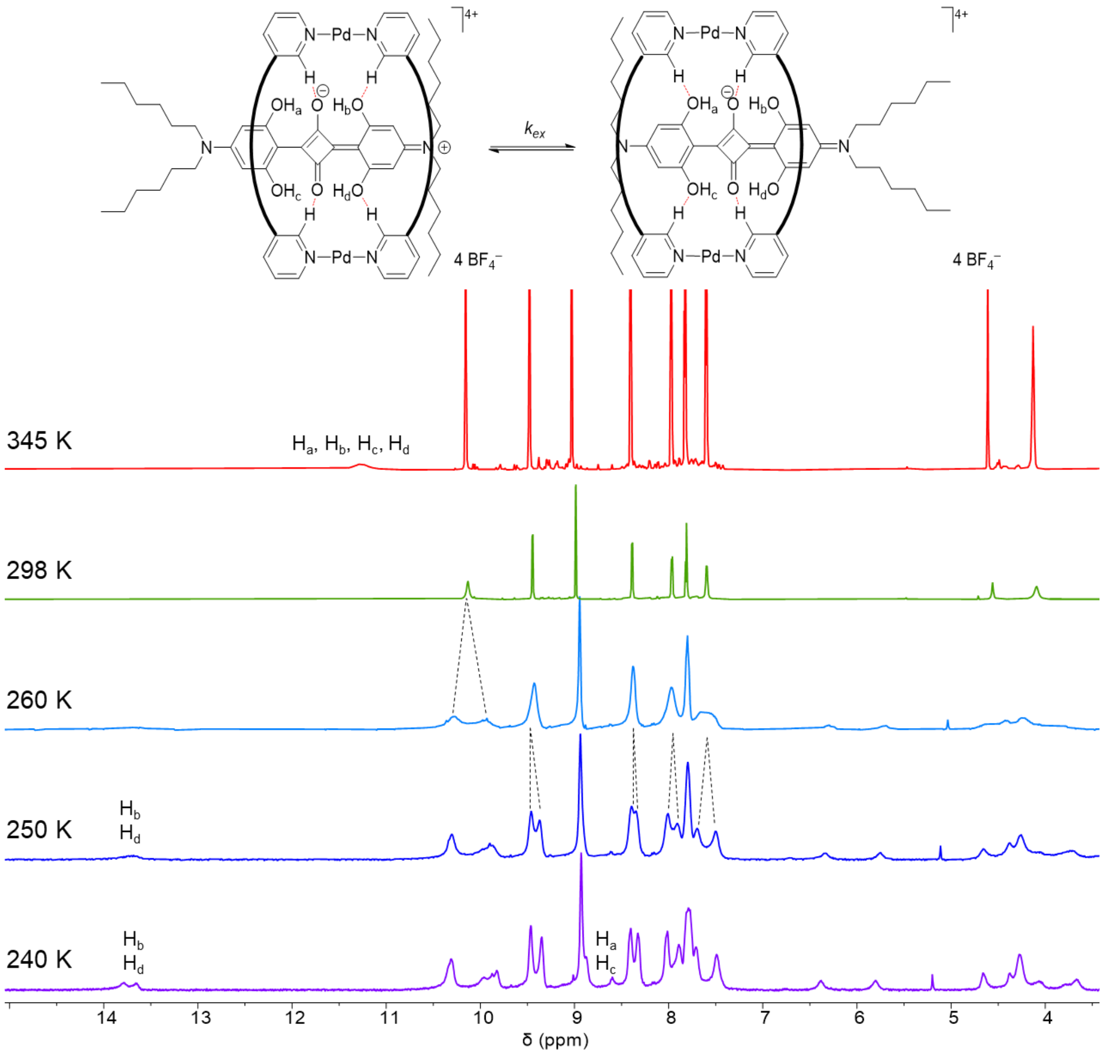

Figure S50. Zoom-in of the aromatic region of the ^{1}H NMR spectra (CD_3CN, 345 K and 298 K: 800 MHz, 260 K–240 K: 400 MHz) of **SQ2⊂2**. Dotted lines highlight the loss of symmetry of the cage's protons upon cooling.

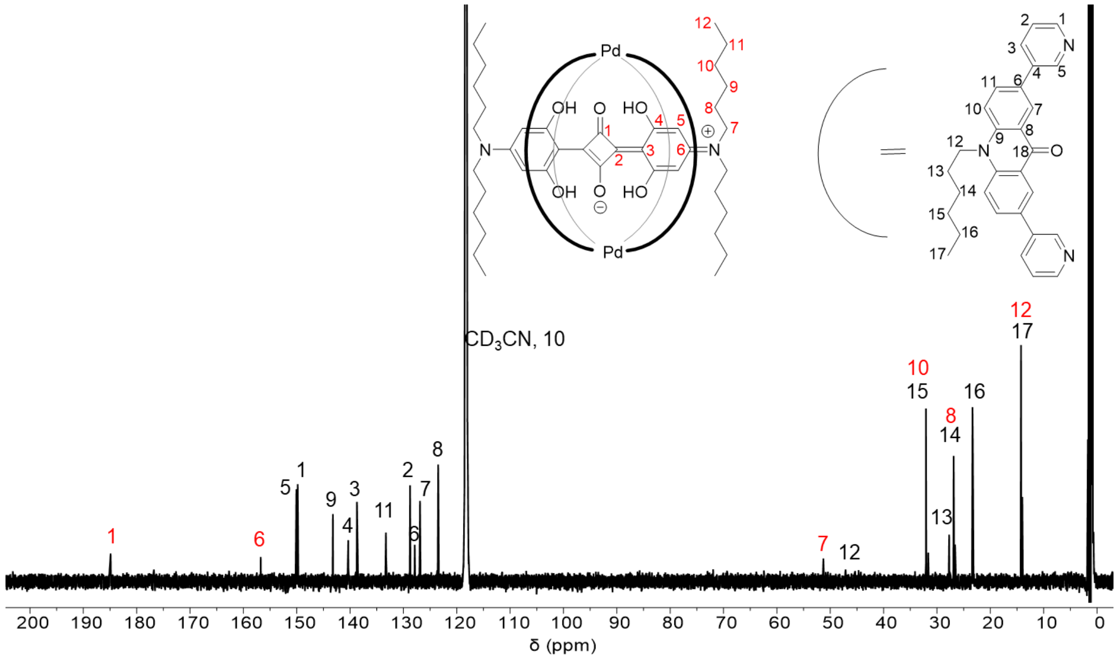

Figure S51. ^{13}C NMR spectrum (298 K, 600 MHz, CD_3CN) of **SQ2⊂2**. Several signals of the dye's carbons are missing due to their intermediate exchange rate.

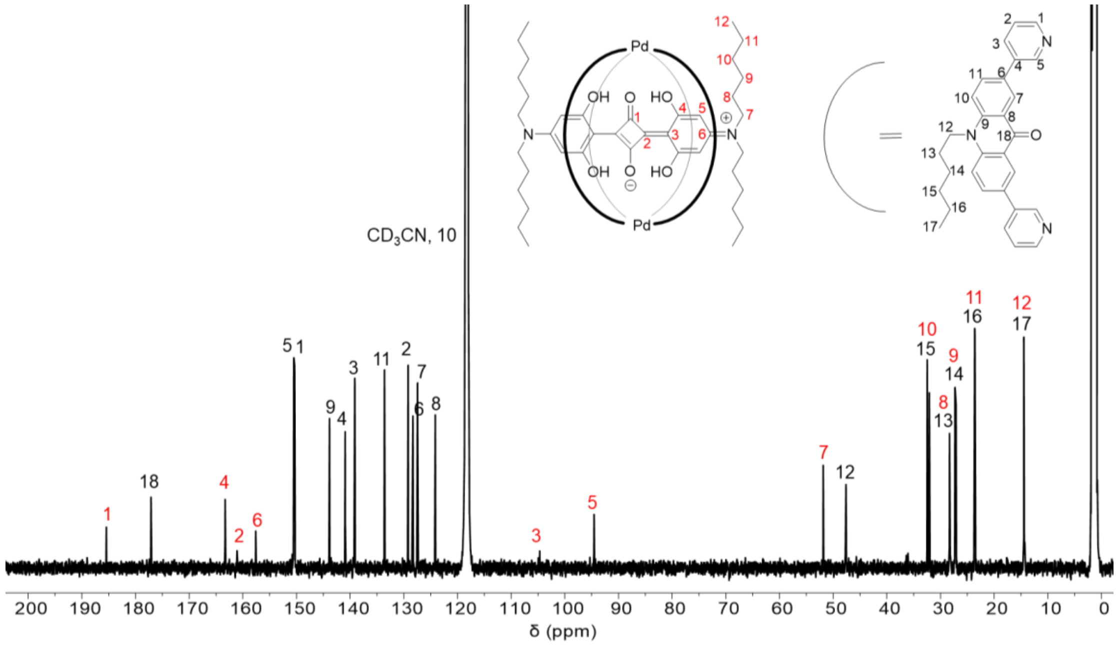

Figure S52. ^{13}C NMR spectrum (345 K, 600 MHz, CD_3CN) of **SQ2⊂2**.

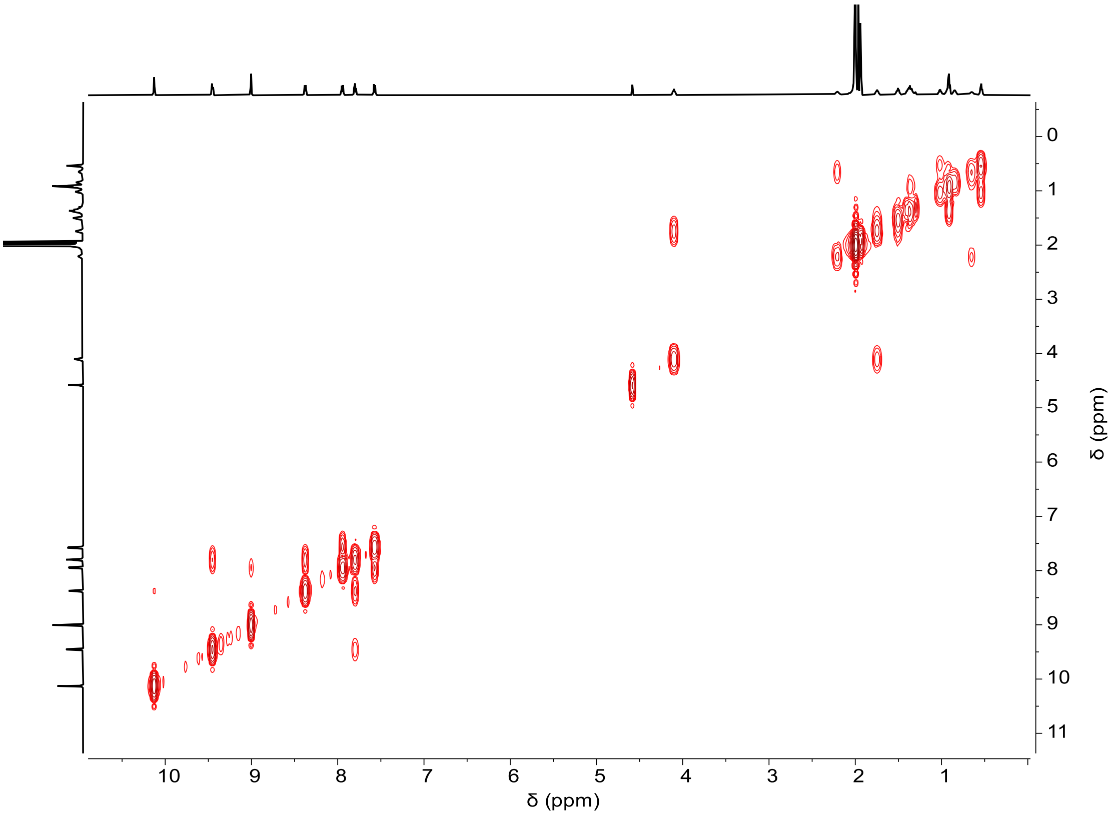

Figure S53. ^{1}H-^{1}H COSY NMR spectrum (345 K, 600 MHz, CD_3CN) of **SQ2⊂2**.

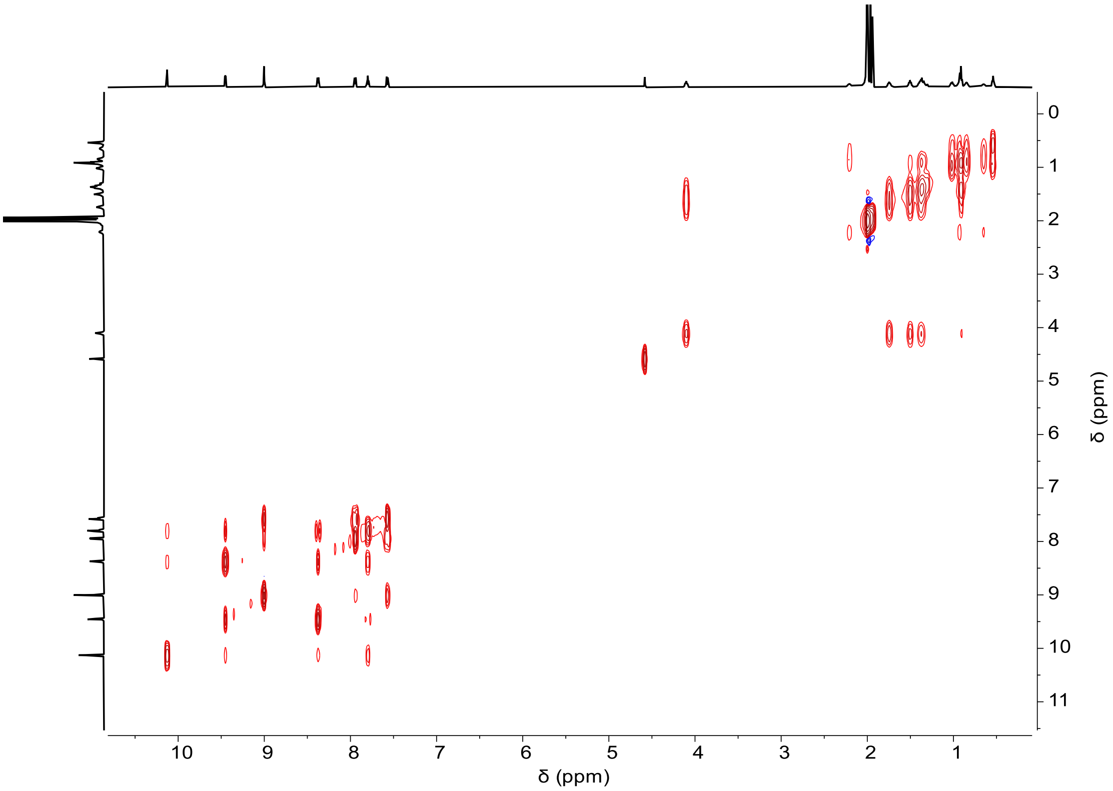

Figure S54. ^{1}H-^{1}H TOCSY NMR spectrum (345 K, 600 MHz, CD_3CN) of **SQ2⊂2**.

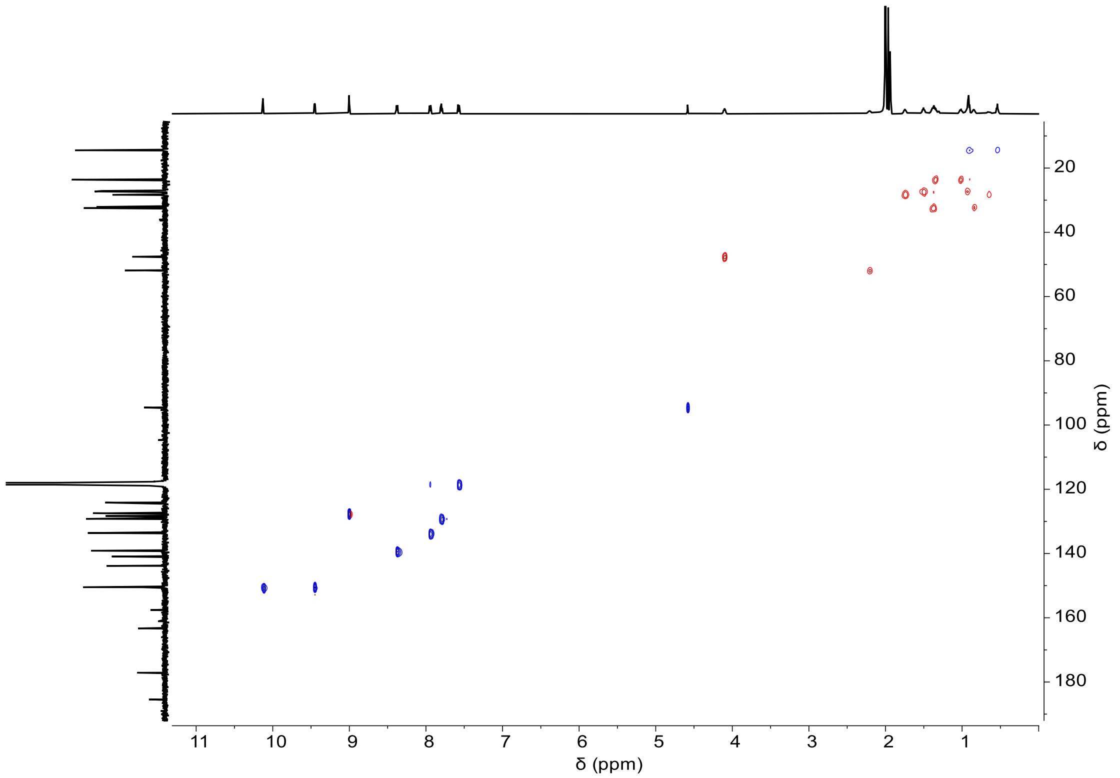

Figure S55. ^{1}H-^{13}C HSQC NMR spectrum (345 K, 600 MHz, CD_3CN) of **SQ2⊂2**.

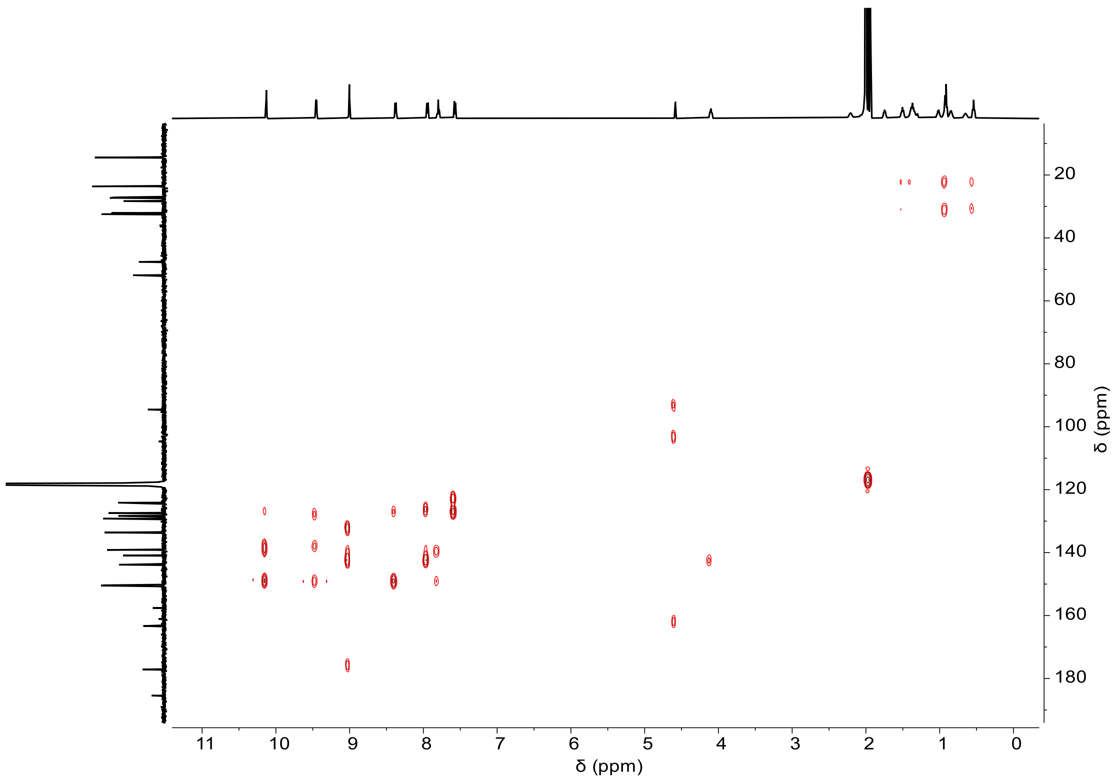

Figure S56. ^{1}H-^{13}C HMBC NMR spectrum (345 K, 600 MHz, CD_3CN) of **SQ2⊂2**.

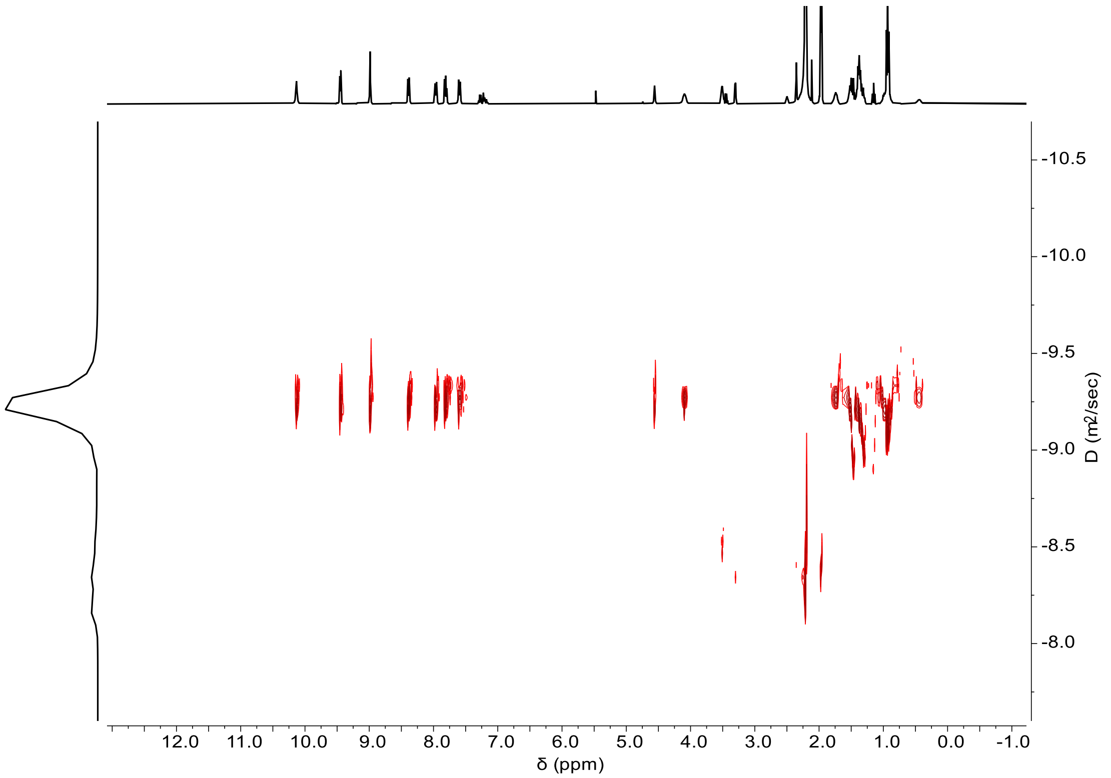

Figure S57. ^{1}H DOSY NMR spectrum (298 K, 400 MHz, CD_3CN) of **SQ2⊂2**, *D* = –9.22 m^2/s.

Attempts to synthesize **SQ3⊂2** via the general procedure described above only yielded the host-guest complex in trace amount.

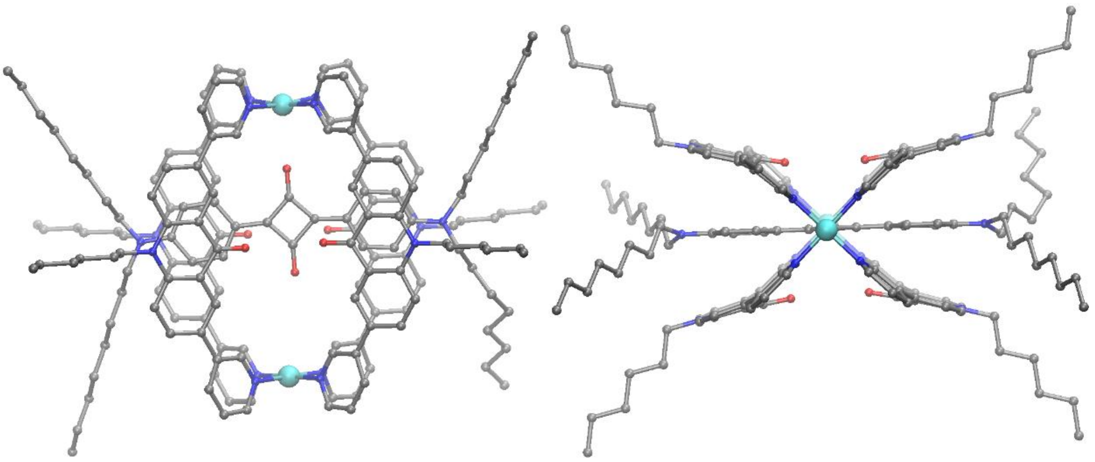

Figure S58. xTB-optimized structure of **SQ3⊂2**, viewed from the side and from the Pd-Pd axis. Hydrogens are omitted for clarity. C: gray, N: blue, O: red, Pd: cyan. For details, see Section 6.

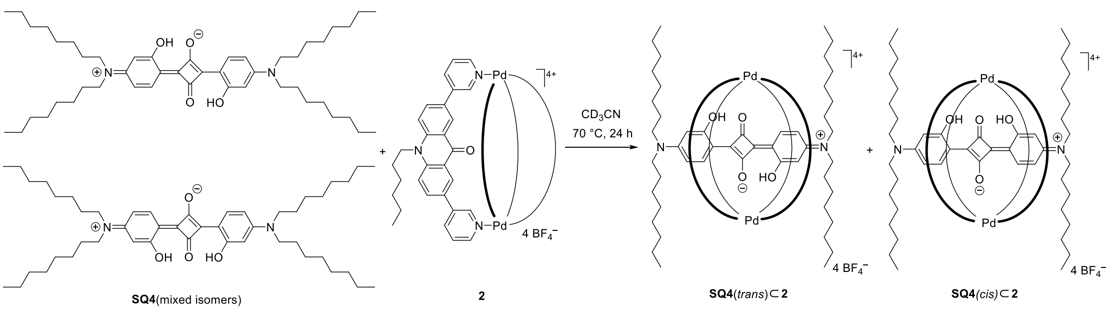

Scheme S9. Synthesis of **SQ4⊂2**.

Similarly to **SQ2⊂2**, **SQ4** could shuttle between two degenerate binding positions, and characterization was carried out at 333 K to make the exchange fast on ^{1}H NMR timescale (See variable temperature NMR studies, Figure S64).

Although **SQ4** was used as a 76:24 mixture of *trans* and *cis* isomers, the resulting mixture of **SQ4**(*trans*)⊂**2** and **SQ4**(*cis*)⊂**2** are in approximately 1:1 ratio. A possible explanation is that the *cis*-*trans* conversion is hindered when **SQ4** is encapsulated in the cage, and that the cage has similar affinity for both isomers.

^{1}H NMR (333K, 600 MHz, CD_3CN) δ 12.54 (s, 2H), 11.84 (s, 2H), 10.42 (d, *J* = 2.1 Hz, 4H), 10.23 (s, 4H), 10.18 (s, 4H), 9.98 (s, 4H), 9.52 (t, *J* = 5.1 Hz, 8H), 9.48 (t, *J* = 5.6 Hz, 8H), 9.09 (d, *J* = 2.5 Hz, 4H), 9.03 (d, *J* = 2.5 Hz, 4H), 8.94 (d, *J* = 2.4 Hz, 4H), 8.91 (d, *J* = 2.4 Hz, 4H), 8.44 – 8.36 (m, 16H), 7.99 – 7.93 (m, 8H), 7.94 – 7.87 (m, 8H), 7.87 – 7.78 (m, 16H), 7.75 (d, *J* = 6.7 Hz, 2H), 7.67 – 7.52 (m, 18H), 4.91 (d, *J* = 9.0 Hz, 2H), 4.69 – 4.64 (m, 4H), 4.61 – 4.53 (m, 2H), 4.23 – 4.08 (m, 16H), 2.37 – 2.21 (m, 16H), 1.82 – 1.70 (m, 16H), 1.55 – 1.46 (m, 16H), 1.44 – 1.29 (m, 32H), 1.12 (p, *J* = 7.4 Hz, 16H), 1.08 – 1.03 (m, 16H), 1.02 – 0.97 (m, 16H), 0.96 – 0.90 (m, 24H), 0.90 – 0.85 (m, 16H), 0.83 (t, *J* = 7.4 Hz, 24H), 0.80 (br.s, 16H), 0.71 (br.s, 16H) (Figure S63).

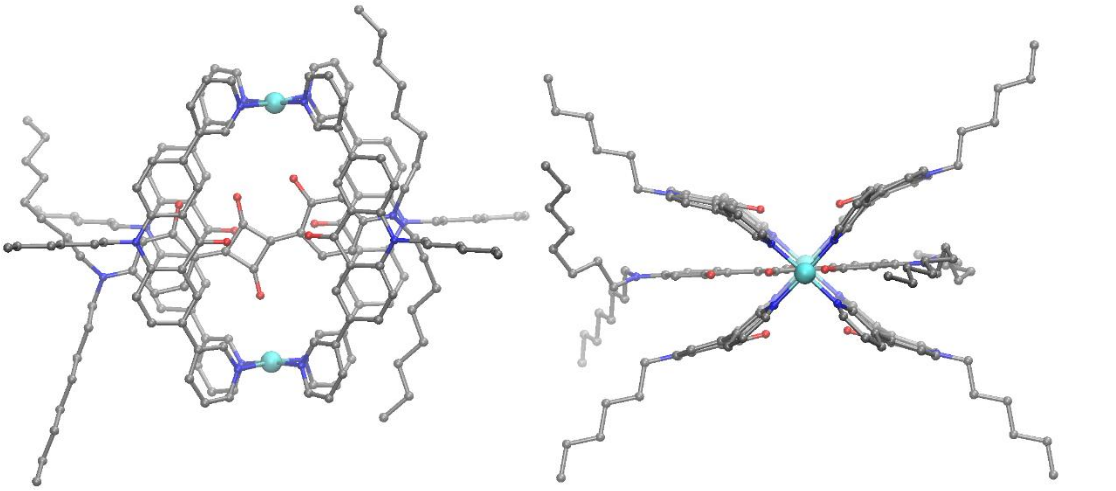

Figure S59. xTB-optimized structure of **SQ4**(*cis*)⊂**2**, viewed from the side and from the Pd-Pd axis. Hydrogens are omitted for clarity. C: gray, N: blue, O: red, Pd: cyan. For details, see Section 6.

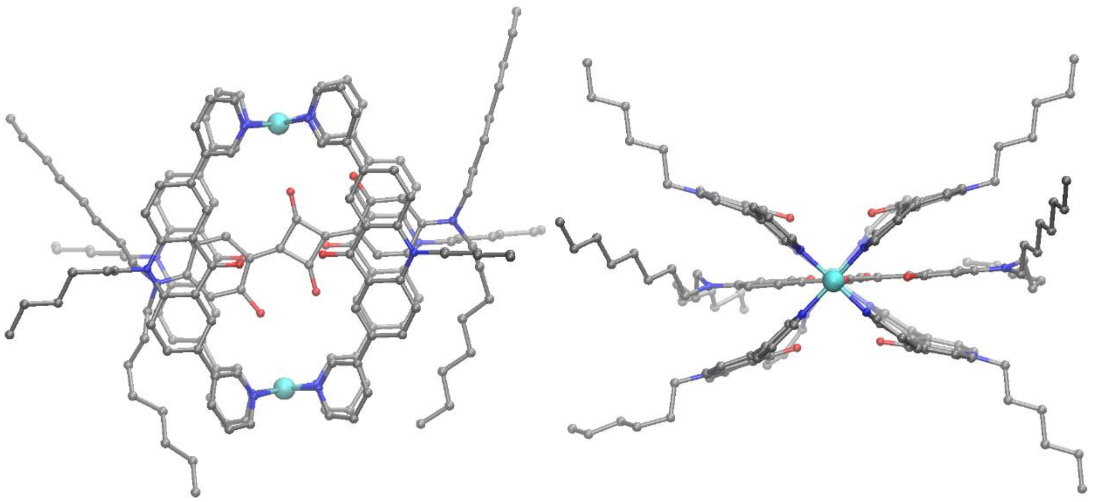

Figure S60. xTB-optimized structure of **SQ4**(*trans*)⊂**2**, viewed from the side and from the Pd-Pd axis. Hydrogens are omitted for clarity. C: gray, N: blue, O: red, Pd: cyan. For details, see Section 6.

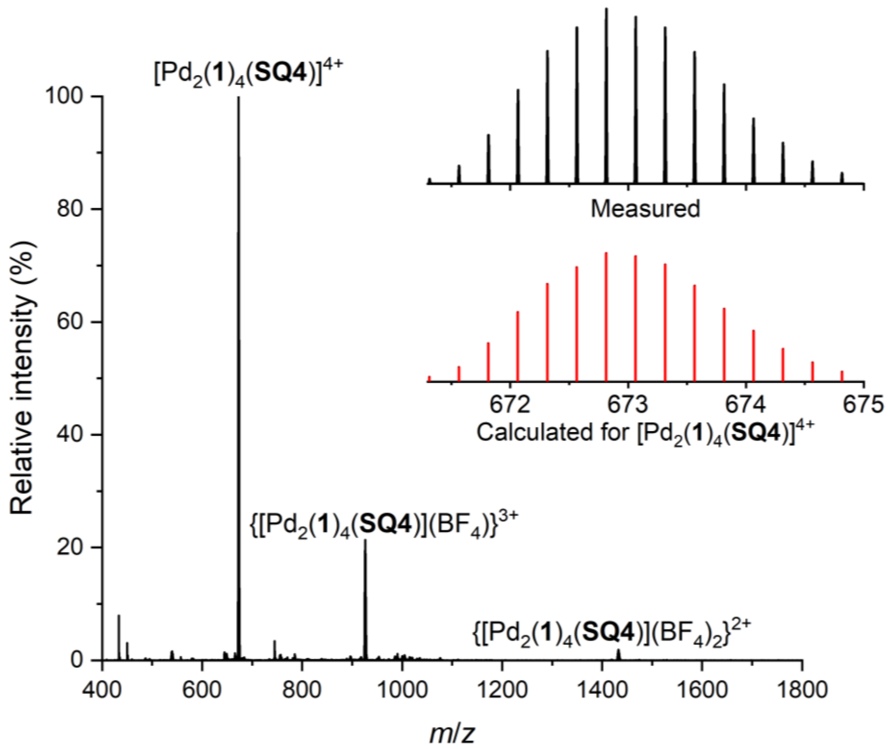

Figure S61. High-Resolution ESI mass spectrum of **SQ4**⊂**2**.

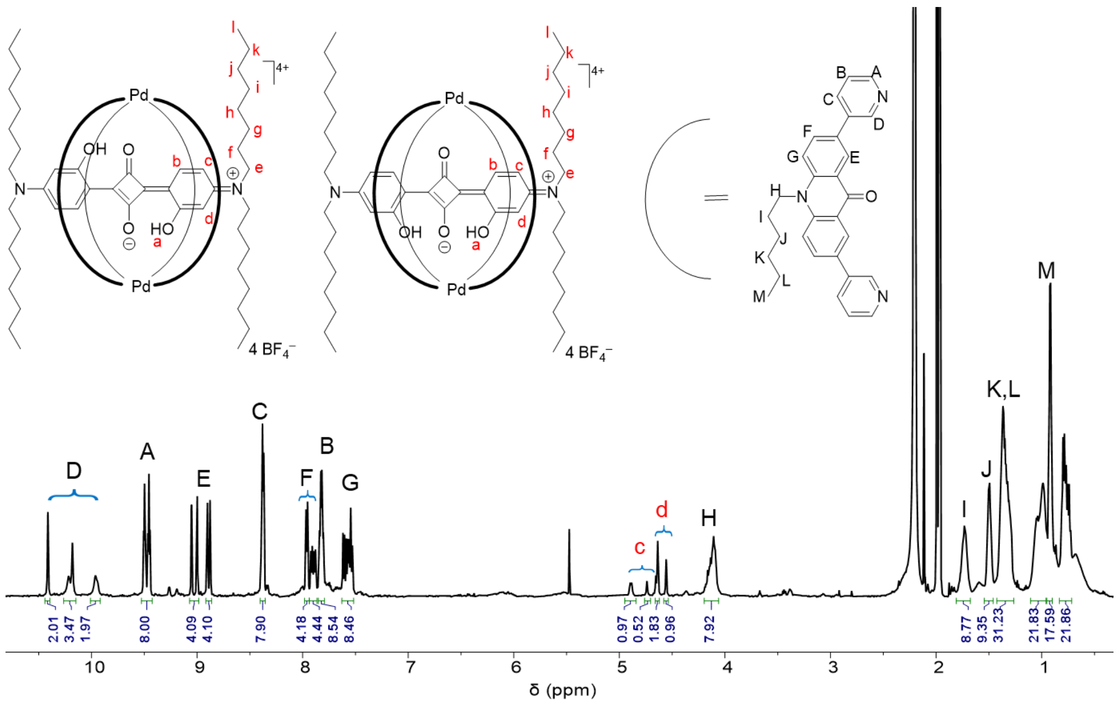

Figure S62. ^{1}H NMR spectrum (298 K, 600 MHz, CD_3CN) of **SQ4⊂2**.

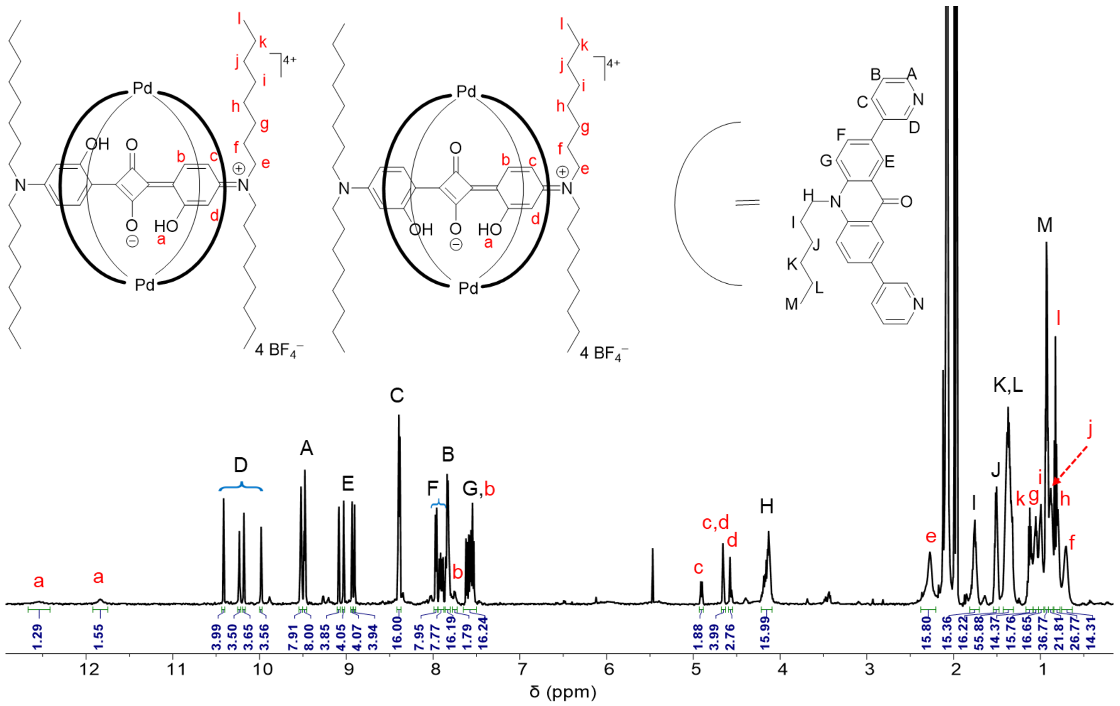

Figure S63. ^{1}H NMR spectrum (333 K, 600 MHz, CD_3CN) of **SQ4⊂2**.

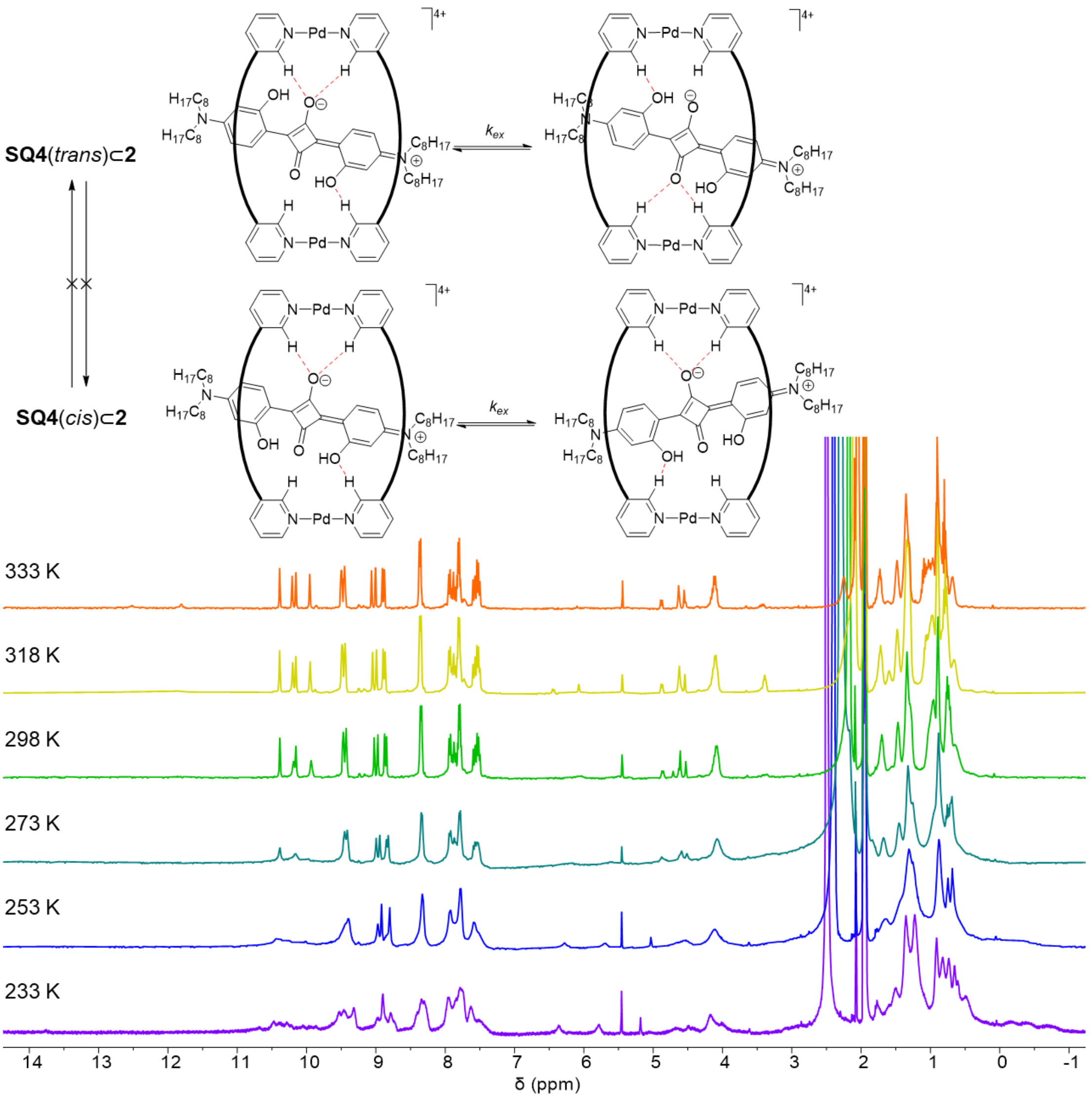

Figure S64. ^{1}H NMR spectrum (333 K–233 K, CD_3CN, 400 MHz) of **SQ2⊂2**. Above 318 K, the shuttling is fast on ^{1}H NMR timescale, and each signal from the cage's protons splits into four: two from **SQ4**(*trans*)**⊂2**, two from **SQ4**(*cis*)**⊂2**. At R.T., the exchange rate of one of the two isomers becomes comparable to ^{1}H NMR timescale, as evidenced by one set of pyridyl protons broadening and one set remaining sharp. Below 253 K, the shuttling of both isomers becomes slow, resulting in lower symmetry.

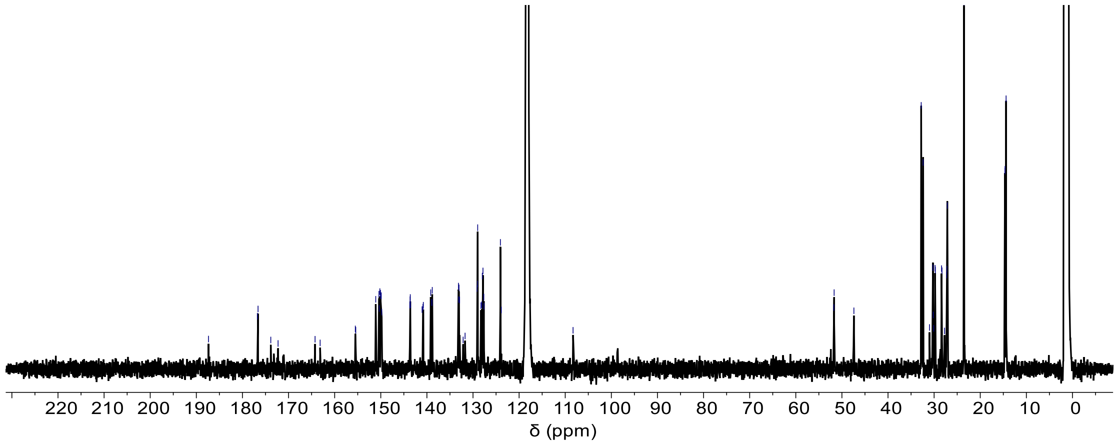

Figure S65. ^{13}C NMR spectrum (333 K, 600 MHz, CD_3CN) of **SQ4⊂2**.

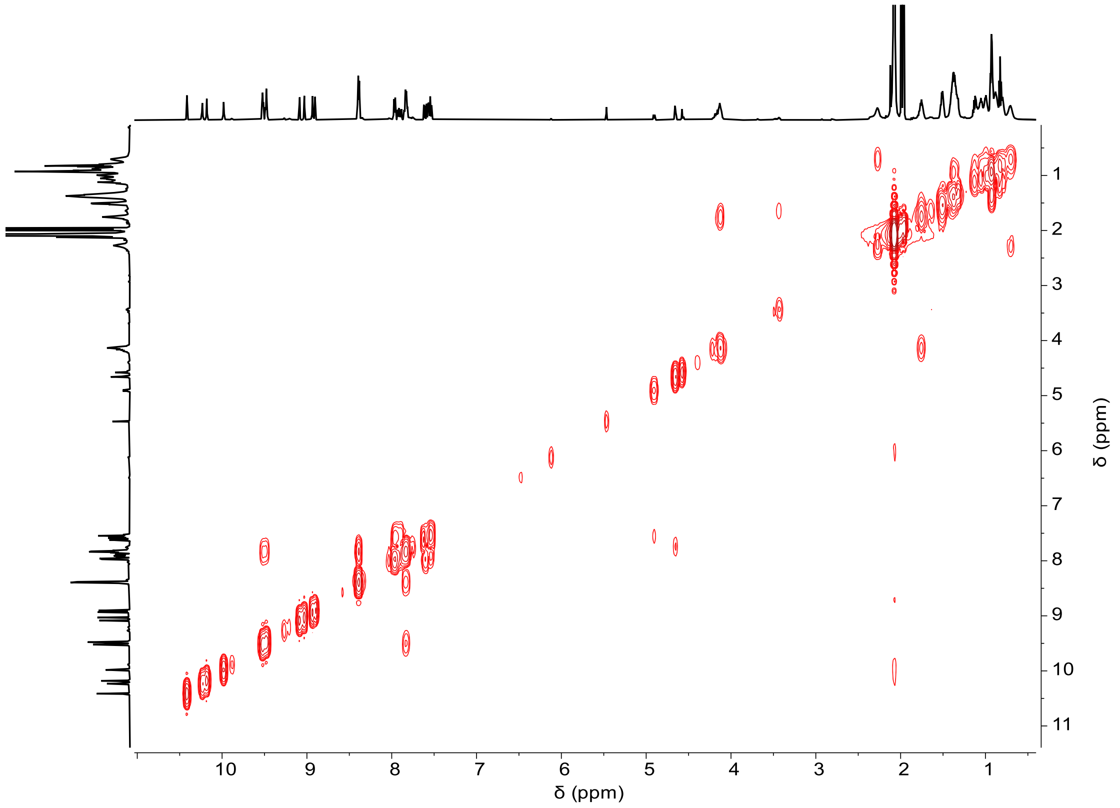

Figure S66. ^{1}H-^{1}H COSY NMR spectrum (333 K, 600 MHz, CD_3CN) of **SQ4⊂2**.

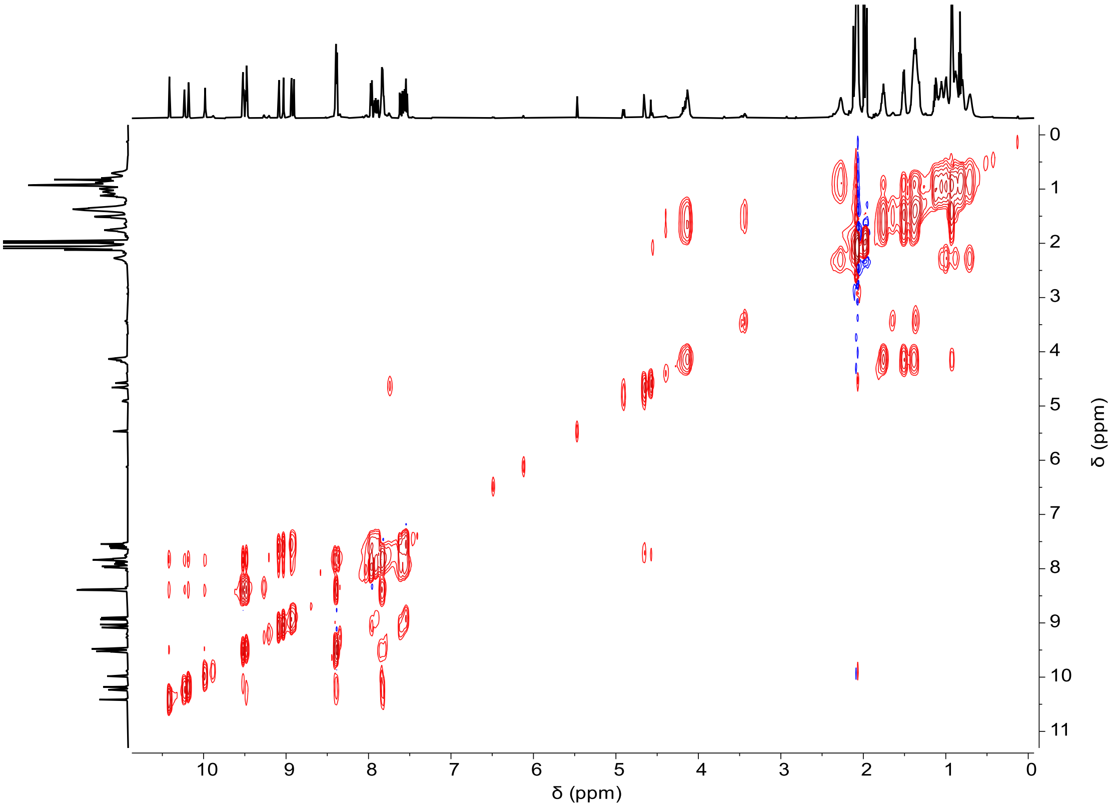

Figure S67. ^{1}H-^{1}H TOCSY NMR spectrum (333 K, 600 MHz, CD_3CN) of **SQ4⊂2**.

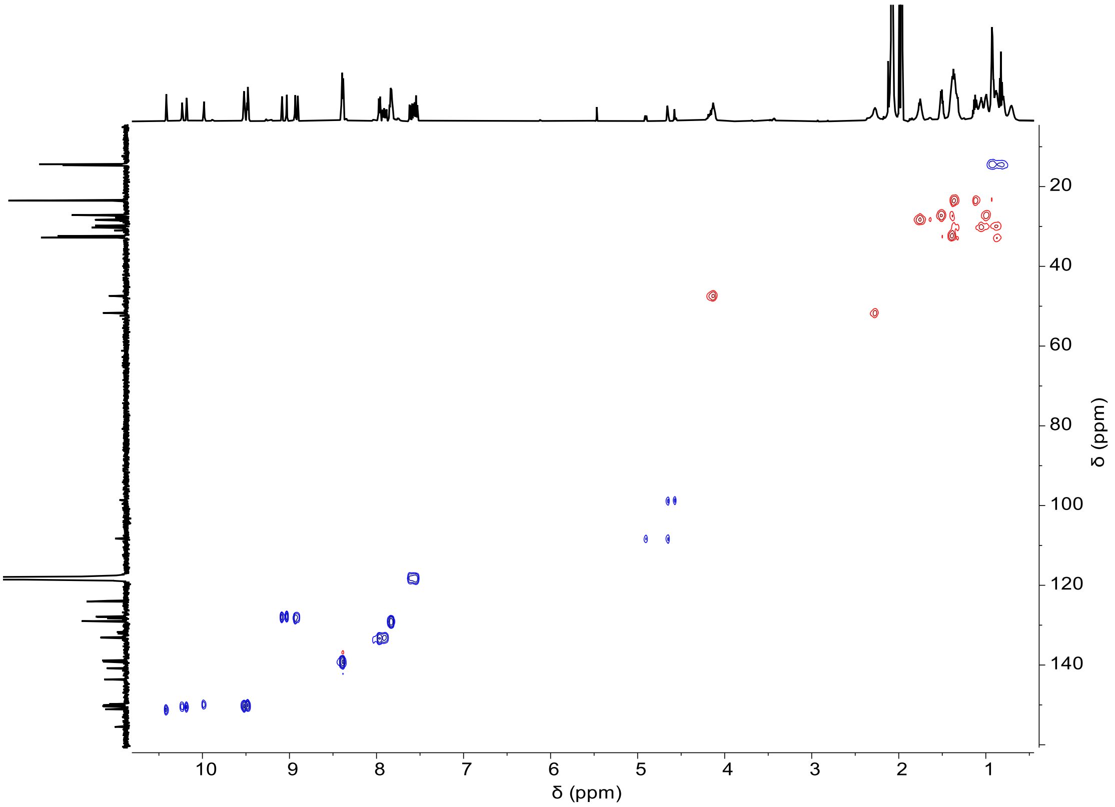

Figure S68. ^{1}H-^{13}C HSQC NMR spectrum (333 K, 600 MHz, CD_3CN) of **SQ4⊂2**.

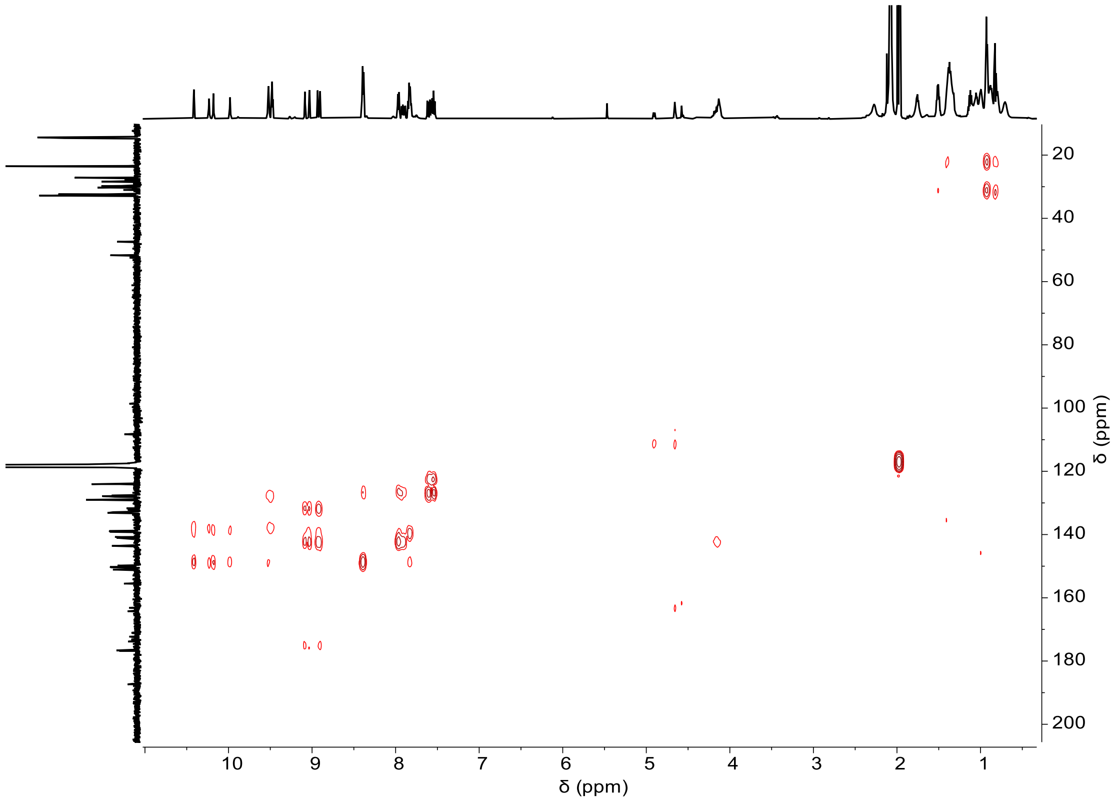

Figure S69. ^{1}H-^{13}C HMBC NMR spectrum (333 K, 600 MHz, CD_3CN) of **SQ4⊂2**.

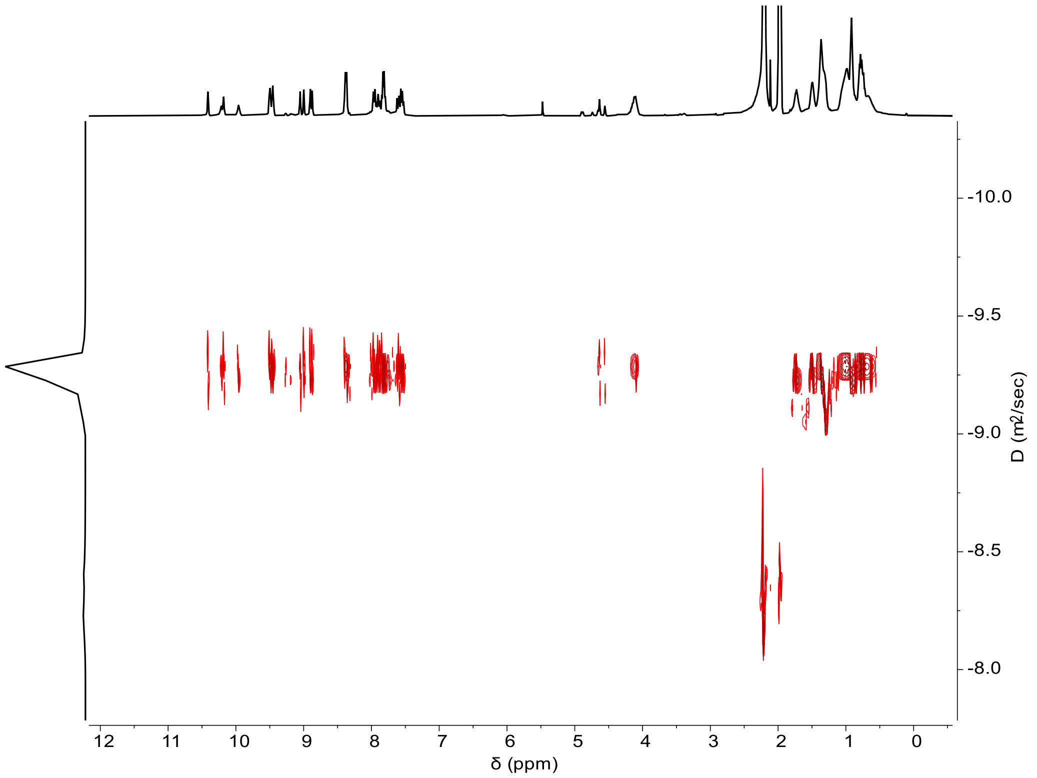

Figure S70. ^{1}H DOSY NMR spectrum (298 K, 400 MHz, CD_3CN) of **SQ4⊂2**, D = –9.29 m^2/s.

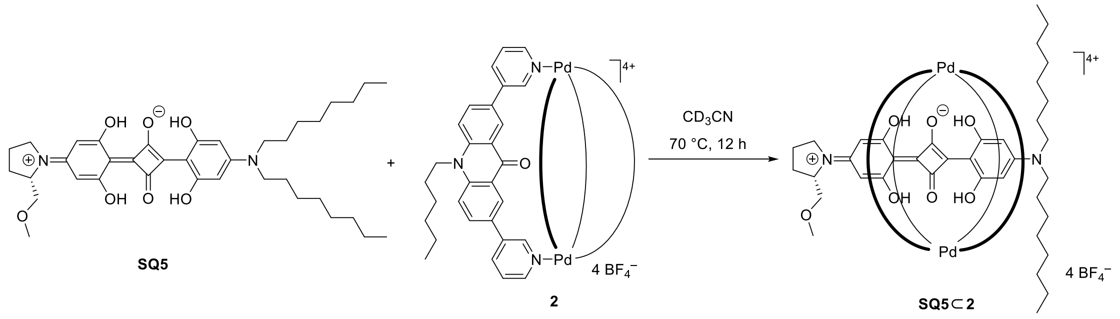

Scheme S10. Synthesis of **SQ5⊂2**.

^{1}H NMR (800 MHz, CD_3CN) δ 13.92 (s, 2H), 13.57 (s, 2H), 10.38 – 10.23 (m, 4H), 9.96 – 9.80 (m, 4H), 9.49 (d, *J* = 6.1 Hz, 4H), 9.39 – 9.31 (m, 4H), 9.02 – 8.94 (m, 4H), 8.91 (d, *J* = 15.2 Hz, 4H), 8.80 (s, 1H), 8.59 (s, 1H), 8.43 – 8.36 (m, 4H), 8.35 – 8.28 (m, 4H), 8.03 – 7.97 (m, 4H), 7.90 (d, *J* = 8.4 Hz, 1H), 7.86 – 7.80 (m, 7H), 7.78 – 7.74 (m, 4H), 7.71 – 7.67 (m, 4H), 7.53 (d, *J* = 8.6 Hz, 1H), 7.49 (t, *J* = 7.6 Hz, 2H), 7.43 (d, *J* = 8.5 Hz, 1H), 4.73 (s, 1H), 4.66 (s, 1H), 4.48 (s, 1H), 4.41 (s, 1H), 4.28 (t, *J* = 8.3 Hz, 4H), 3.93 – 3.71 (m, 5H), 3.43 (s, 3H), 3.33 – 3.26 (m, 3H), 3.15 (br.s, 1H), 2.08 – 2.01 (m, 4H), 1.82 – 1.76 (m, 4H), 1.67 – 1.59 (m, 4H), 1.55 (p, *J* = 7.3 Hz, 4H), 1.45 – 1.19 (m, 24H), 0.94 – 0.86 (m, 16H), 0.80 – 0.67 (m, 10H), 0.56 – 0.52 (m, 4H), 0.48 (br.s, 4H), 0.09 – –0.01 (m, 4H), –0.03 – –0.10 (m, 4H), –0.35 (br.s, 4H) (Figure S73).

^{13}C NMR (151 MHz, CD_3CN) δ 184.79, 177.56, 176.04, 163.87, 163.73, 162.71, 161.65, 161.21, 158.09, 156.52, 156.01, 150.38, 150.24, 150.03, 149.84, 143.50, 143.01, 140.90, 140.10, 139.02, 138.88, 138.62, 133.84, 133.02, 132.89, 128.87, 128.69, 128.55, 128.39, 127.56, 127.41, 126.97, 126.83, 123.51, 123.41, 107.20, 101.37, 96.17, 95.84, 93.20, 92.60, 74.29, 60.06, 59.73, 50.78, 49.70, 47.39, 32.43, 32.10, 32.03, 30.29, 29.44, 29.29, 29.14, 27.93, 27.01, 26.87, 26.81, 26.47, 23.47, 23.25, 14.63, 14.25 (Figure S74). The reduced symmetry resulted in low intensity in the ^{13}C spectrum, and not all the carbons could be detected.

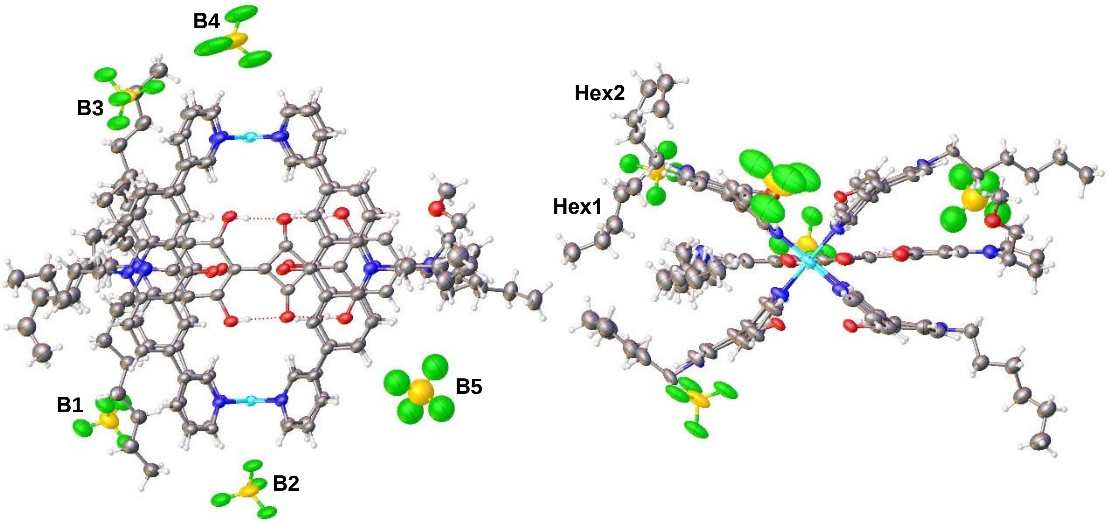

Figure S71. Solid-state structure of **SQ5⊂2** as determined by single-crystal XRD, viewed from the side and from the Pd-Pd axis, with thermal ellipsoids drawn at the 20% probability level. C: grey, N: blue, O: red, H: white, F: green, B: yellow, and Pd: cyan. The BF_4^- anions B3 and B5 have an occupancy of 50%. Alkyl chains Hex1 and Hex2 have occupancies of 53.8% and 46.2% respectively.

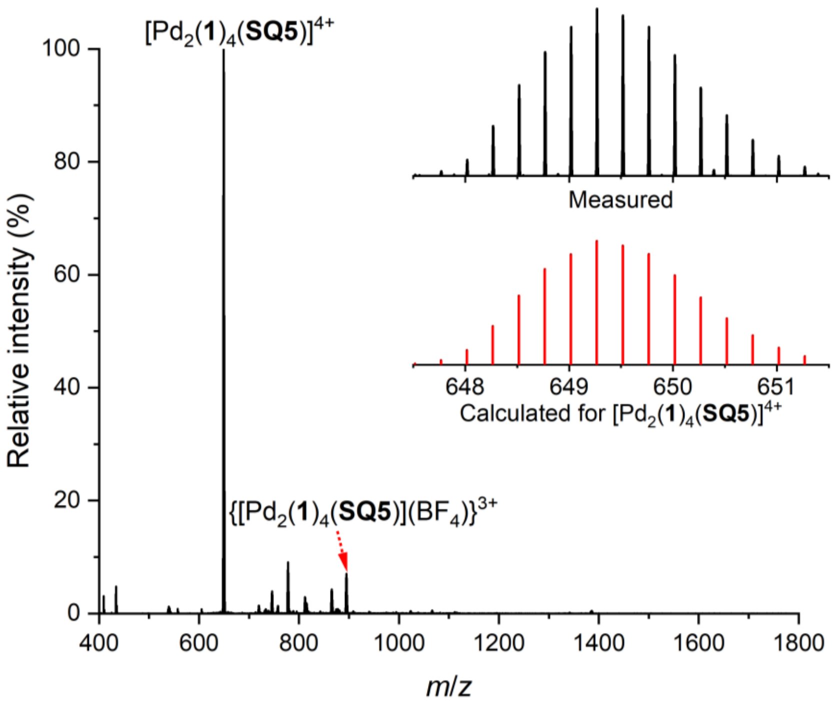

Figure S72. High-Resolution ESI mass spectrum of **SQ5⊂2**.

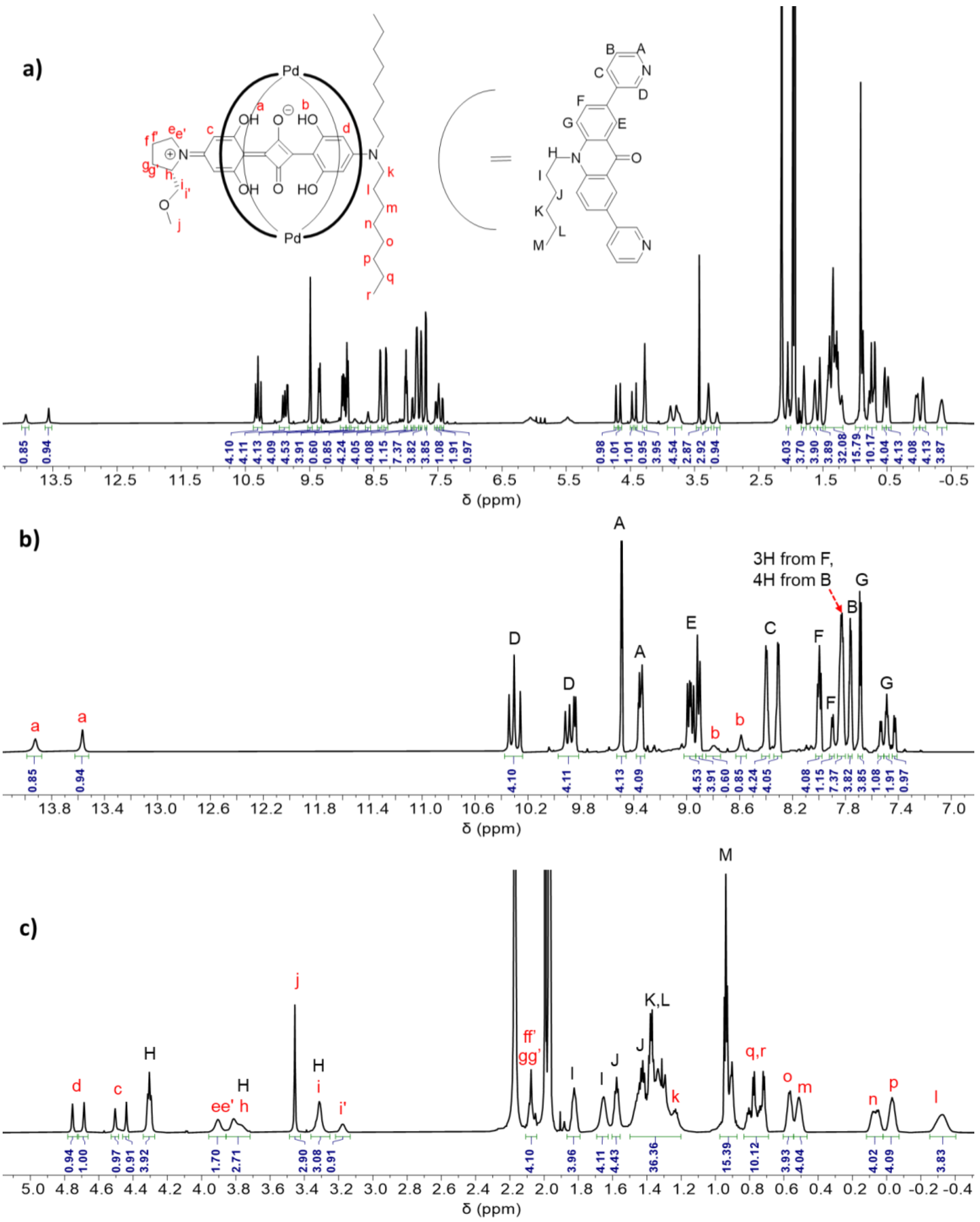

Figure S73. ^{1}H NMR spectrum (800 MHz, CD_3CN) of **SQ5⊂2**. (**a**) Full spectrum, (**b**) 14 to 7 ppm region and (**c**) 5 to –0.5 ppm region. For clarity, assignment was labeled on the zoomed-in parts. Each aromatic proton of the cage is split into 8, showing that the encapsulation of **SQ5** resulted in a lower symmetry. Similarly to **SQ1⊂2**, protons l, n and p of the dye are particularly shielded due to C–H --- π interactions

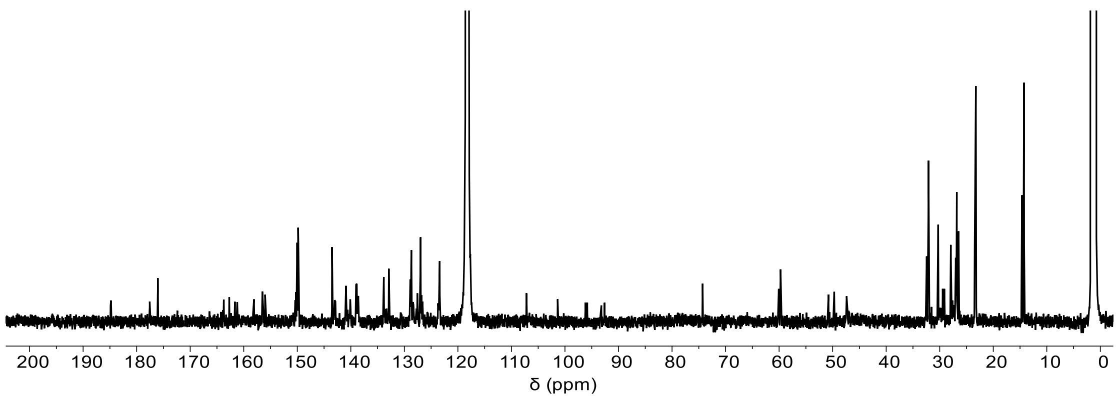

Figure S74. ^{13}C NMR spectrum (600 MHz, CD_3CN) of **SQ5⊂2**.

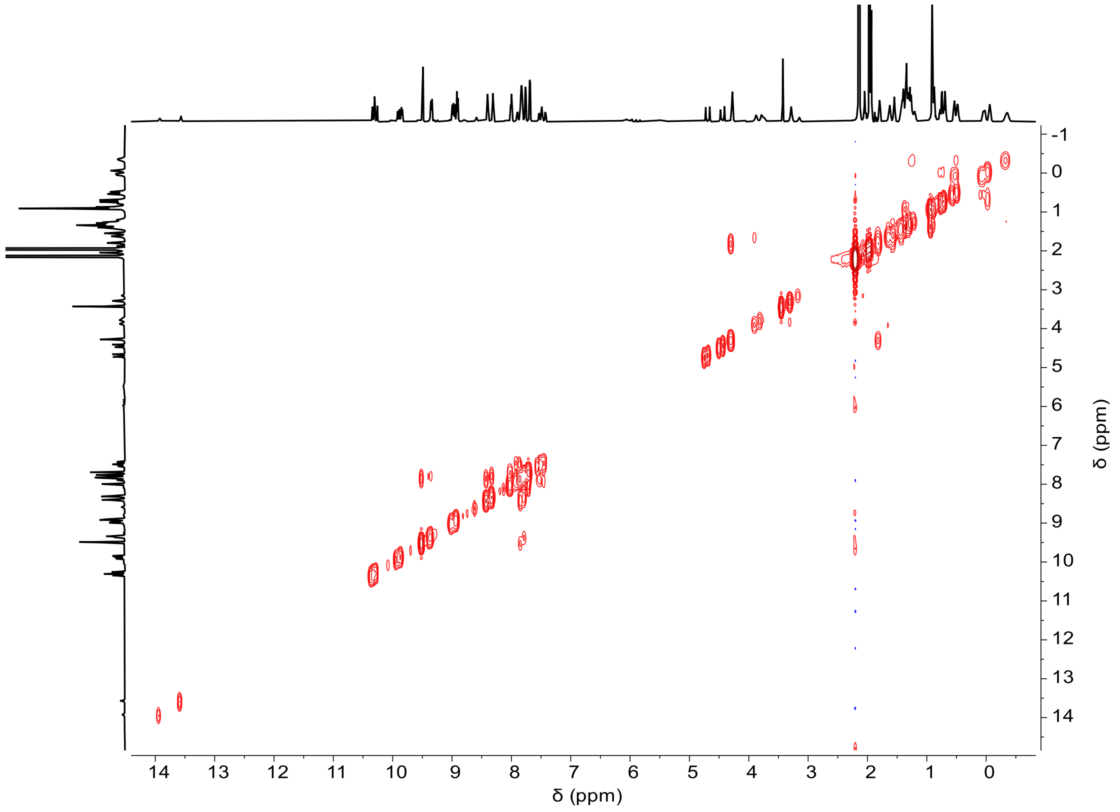

Figure S75. ^{1}H-^{1}H COSY NMR spectrum (600 MHz, CD_3CN) of **SQ5⊂2**.

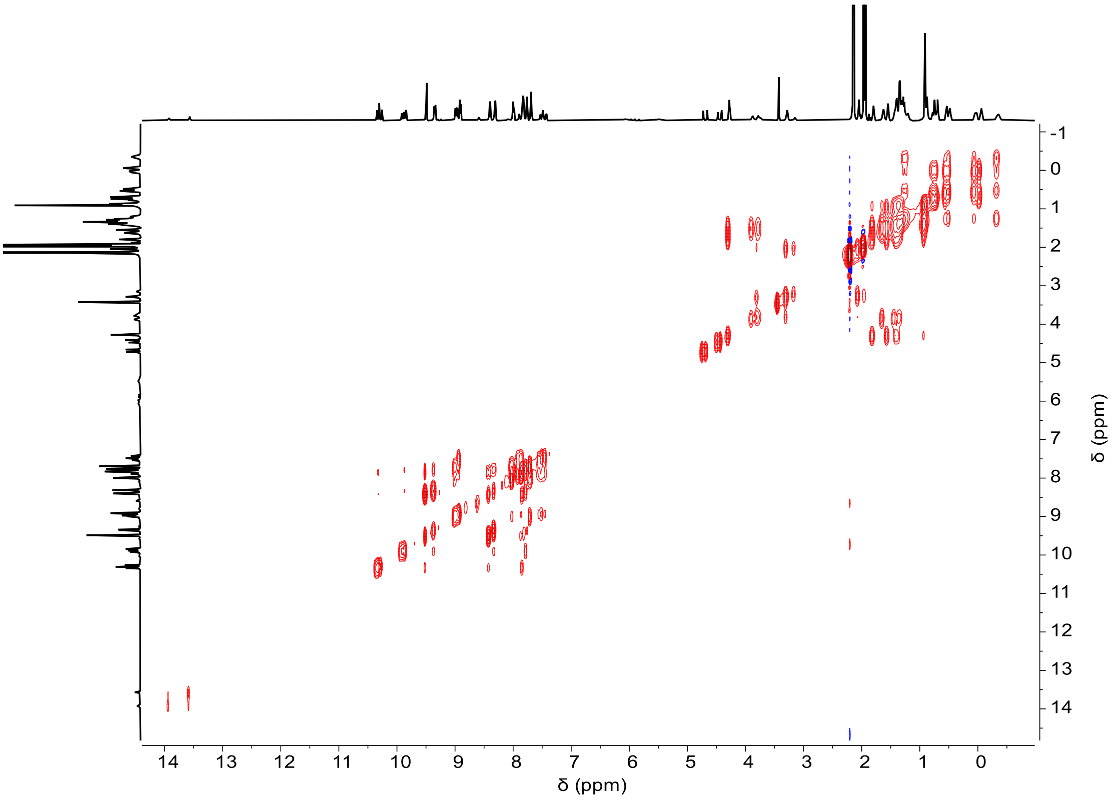

Figure S76. ^{1}H-^{1}H TOCSY NMR spectrum (600 MHz, CD_3CN) of **SQ5⊂2**.

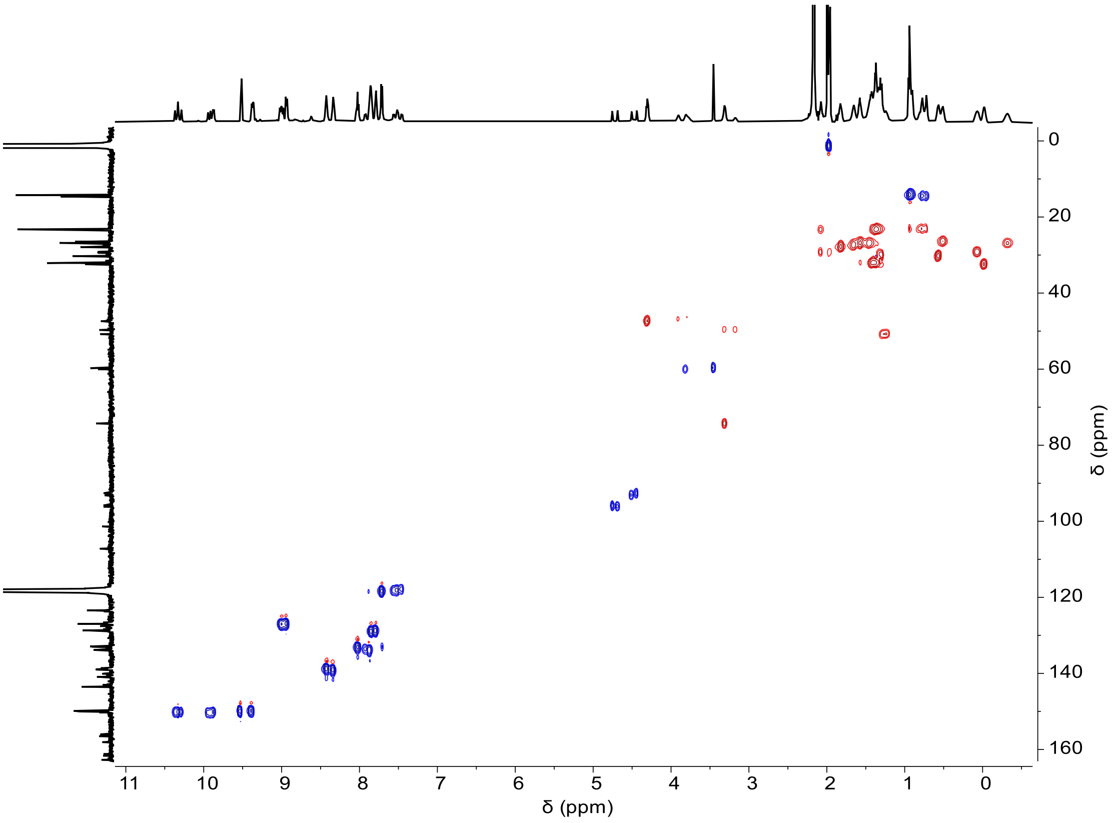

Figure S77. ^{1}H-^{13}C HSQC NMR spectrum (600 MHz, CD_3CN) of **SQ5⊂2**.

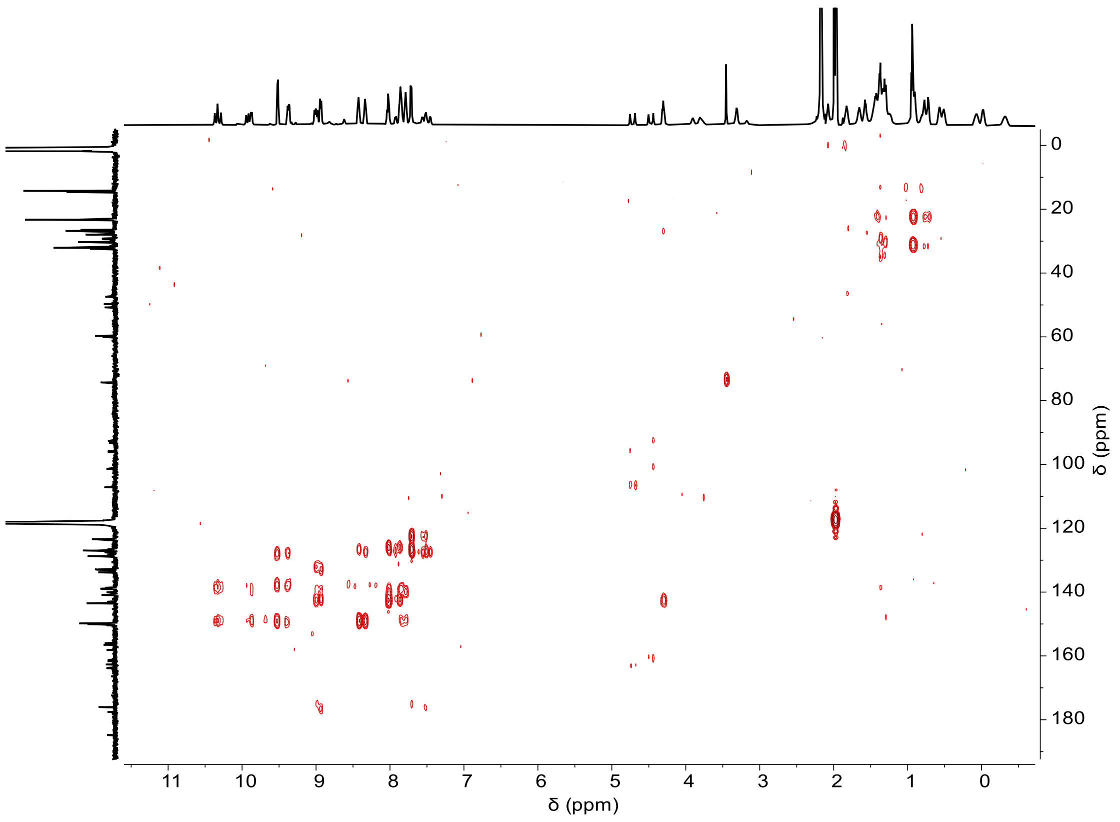

Figure S78. ^{1}H-^{13}C HMBC NMR spectrum (600 MHz, CD_3CN) of **SQ5⊂2**.

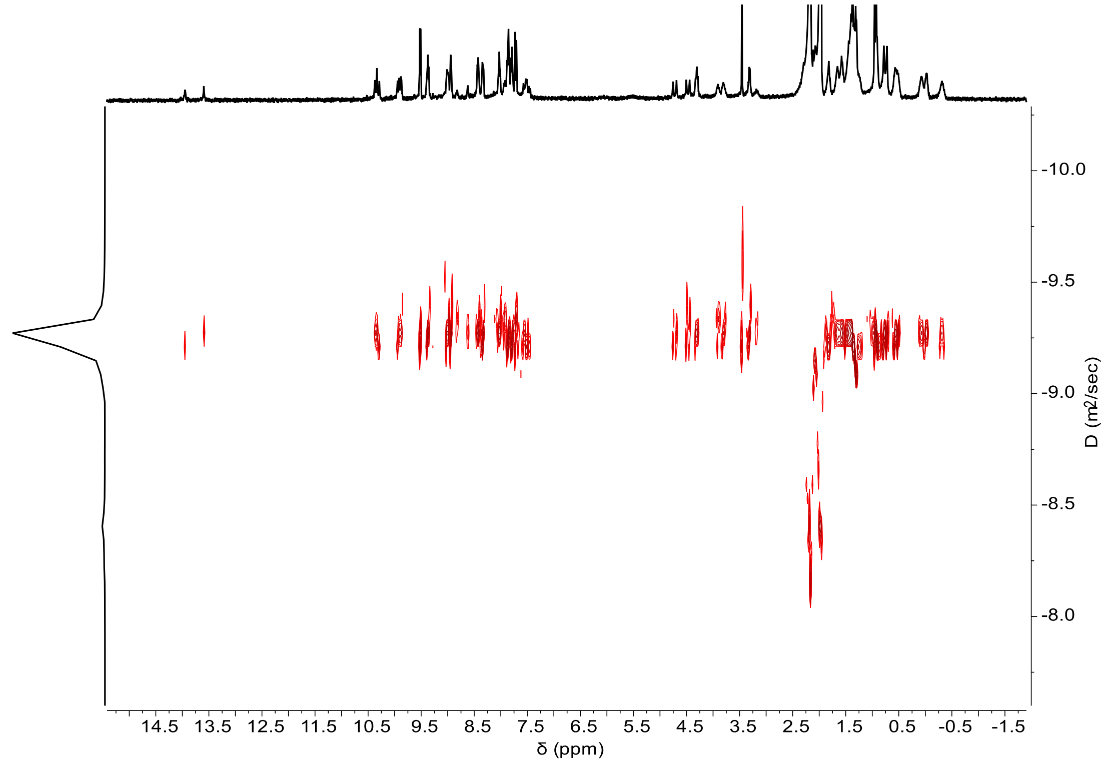

Figure S79. ^{1}H DOSY NMR spectrum (400 MHz, CD_3CN) of **SQ5⊂2**, D = –9.26 m^2/s.

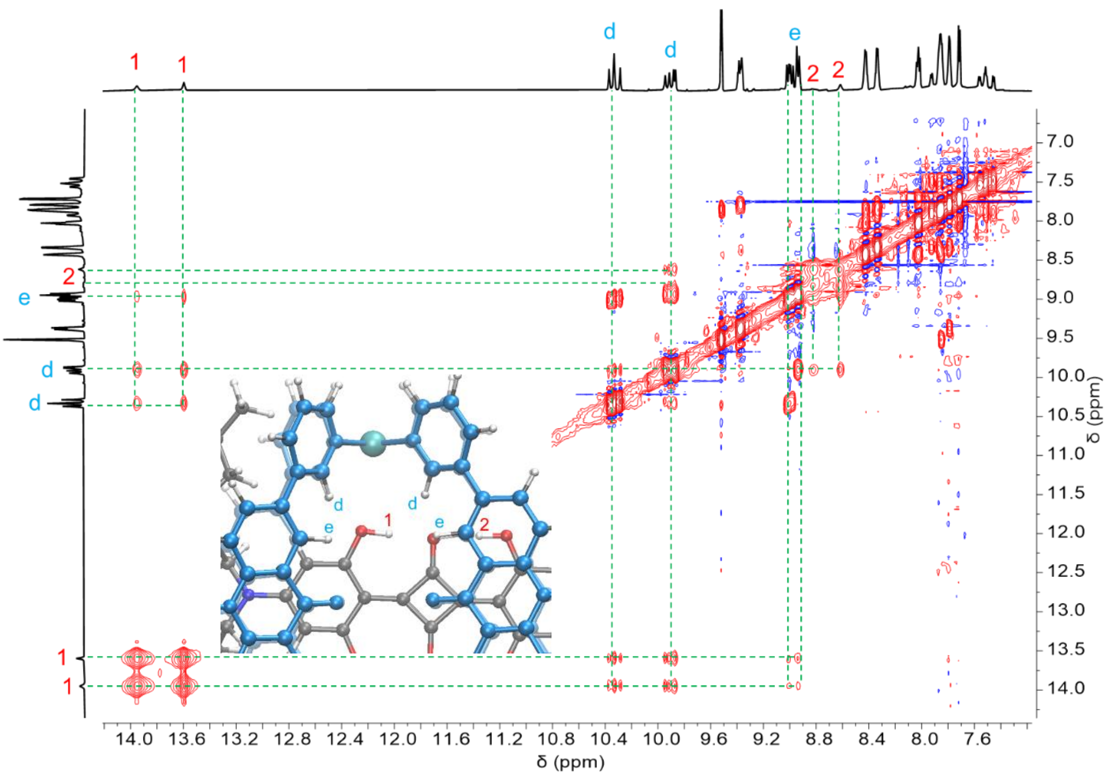

Figure S80. ^1H-^1H NOESY NMR spectrum (800 MHz, CD_3CN) of **SQ5⊂2**. Insert: binding motif illustrated with the xTB-optimized structure. Green dotted lines highlight cross peaks between OH protons of **SQ5** (H_1 and H_2) and inward-pointing protons of **2** (H_d and H_e). The two centered H_1 are close to all 8 H_d protons, while two the off-centered H_2 are only close to 4 of the H_d protons.

3. Host-guest studies

3.1. NMR titration experiments

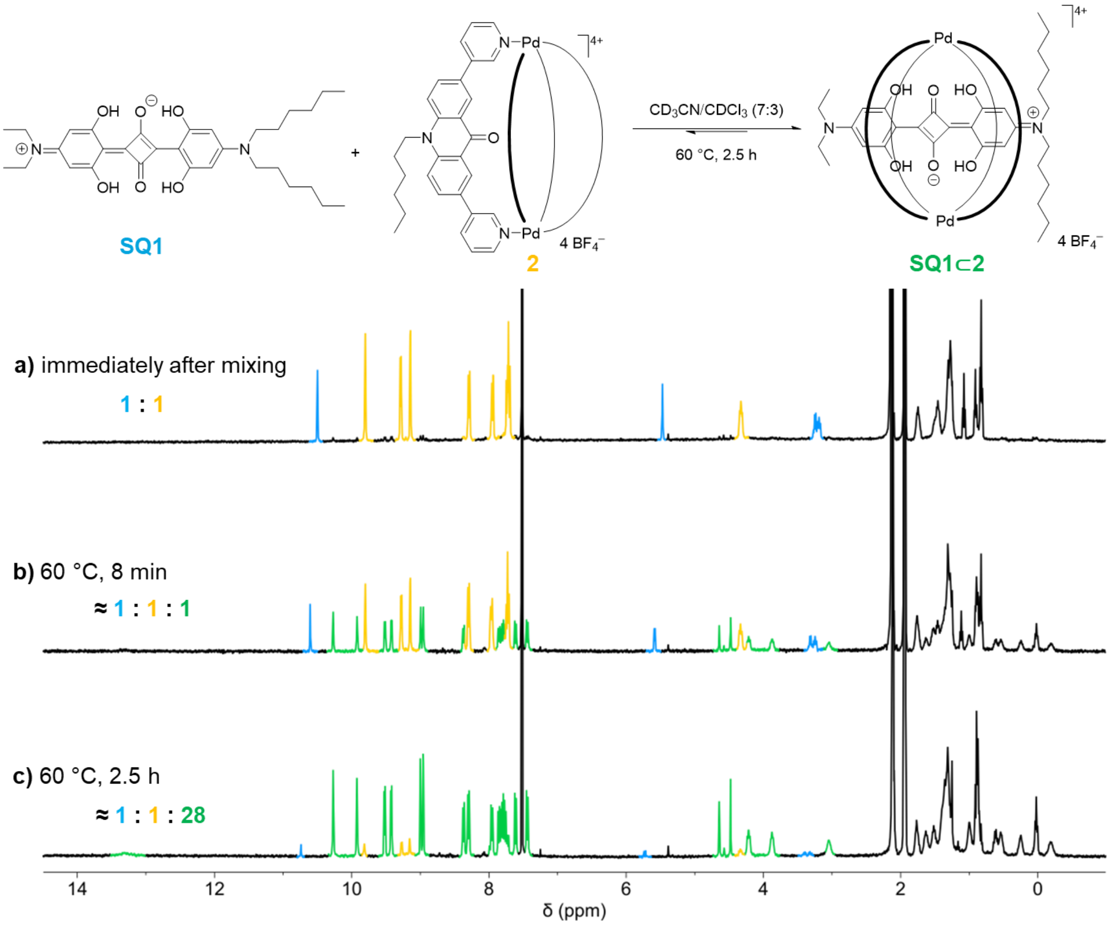

Figure S81. ^{1}H NMR spectra (400 MHz) of a mixture of **SQ1** (0.7 mM) and **2** (0.7 mM) in $CD_3CN/CDCl_3$ (7:3). (**a**) Immediately after mixing. (**b**) After heating to 60 °C for 8 min, approx. 50% of the receptor **2** were bound. (**c**) After heating for 2.5 h, equilibrium was reached.

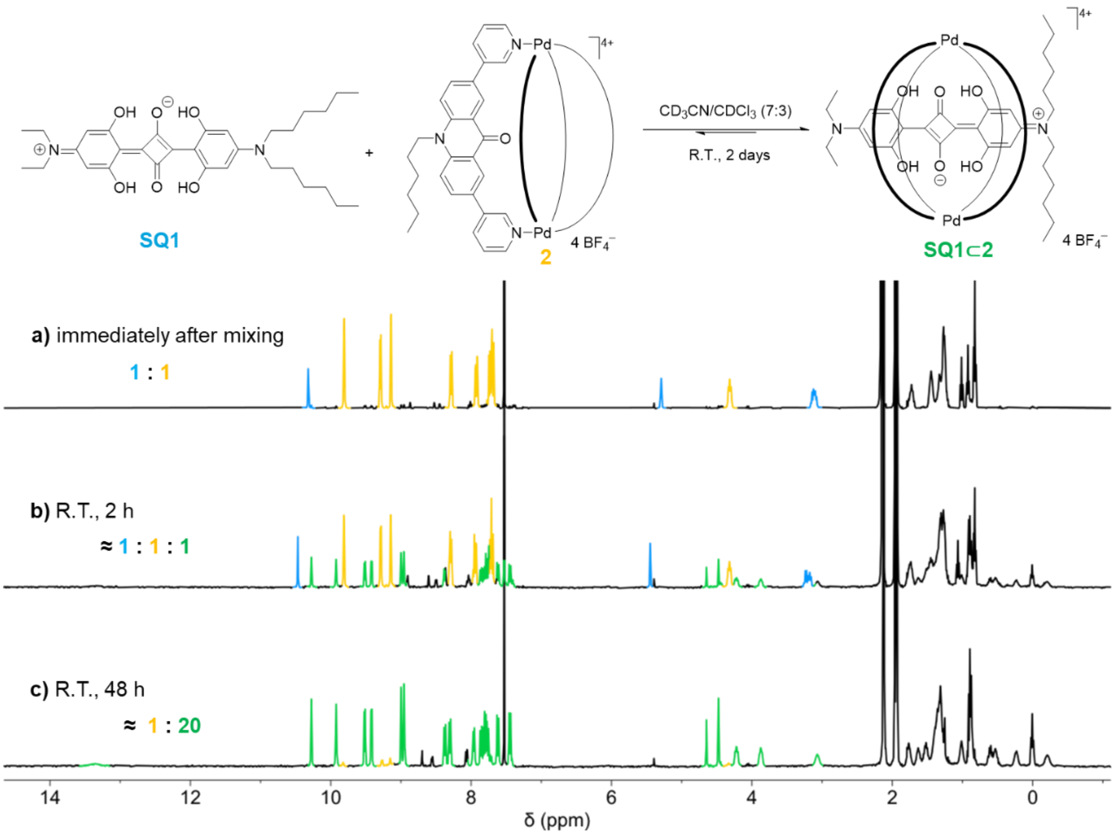

Figure S82. ^{1}H NMR spectra (400 MHz) of a mixture of **SQ1** (1.05 mM) and **2** (1.05 mM) in $CD_3CN/CDCl_3$ (7:3). (**a**) Immediately after mixing. (**b**) After 2 h at room temperature, approx. 50% of the receptor **2** were bound. (**c**) After 2 days at room temperature, equilibrium was reached.

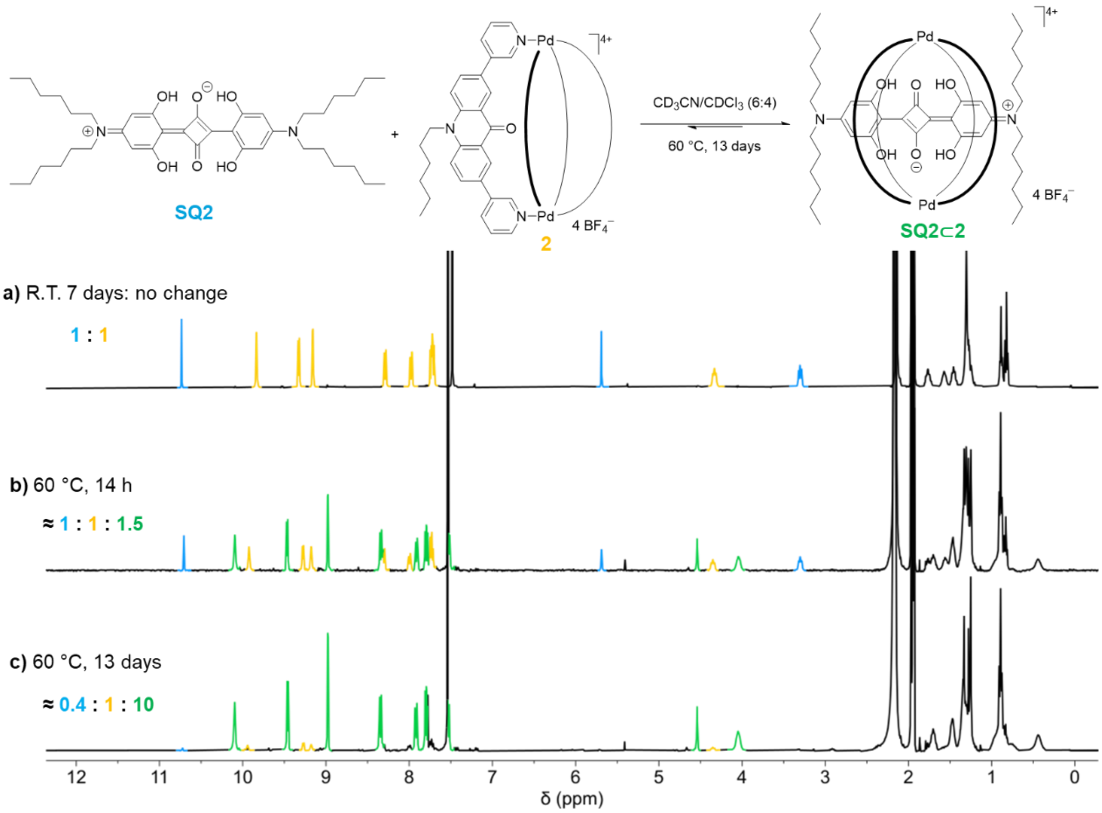

Figure S83. ^{1}H NMR spectra (400 MHz) of a mixture of **SQ2** (0.77 mM) and **2** (0.77 mM) in $CD_3CN/CDCl_3$ (6:4). (**a**) the spectrum remained unchanged for a week at R.T. (**b**) After 14 h at 60 °C, approx. 60% of the receptor **2** were bound. (**c**) After 13 days at 60 °C, equilibrium was reached.

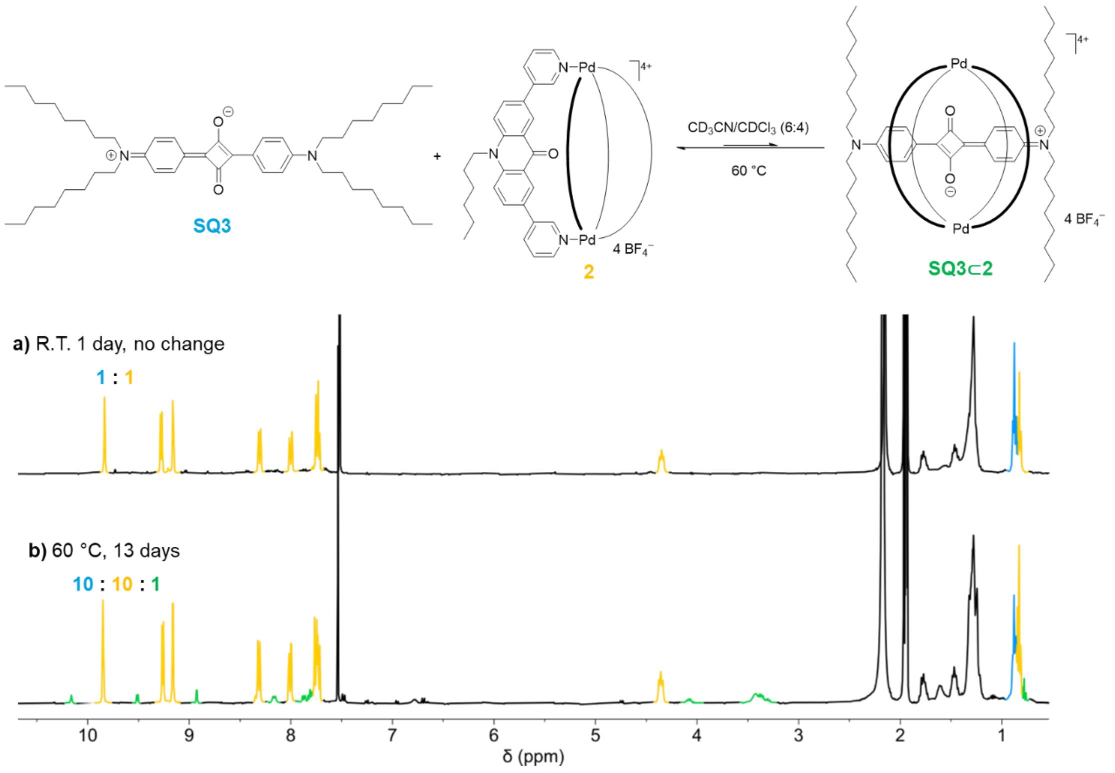

Figure S84. ^{1}H NMR spectra (400 MHz) of a mixture of **SQ3** (0.9 mM) and **2** (0.9 mM) in $CD_3CN/CDCl_3$ (6:4). (**a**) the spectrum remained unchanged for 1 day at R.T. (**b**) Only trace amounts of a new species were observed after 13 days at 60 °C. Heating for longer resulted in decomposition of **SQ3**, with a color change from blue-green to brown.

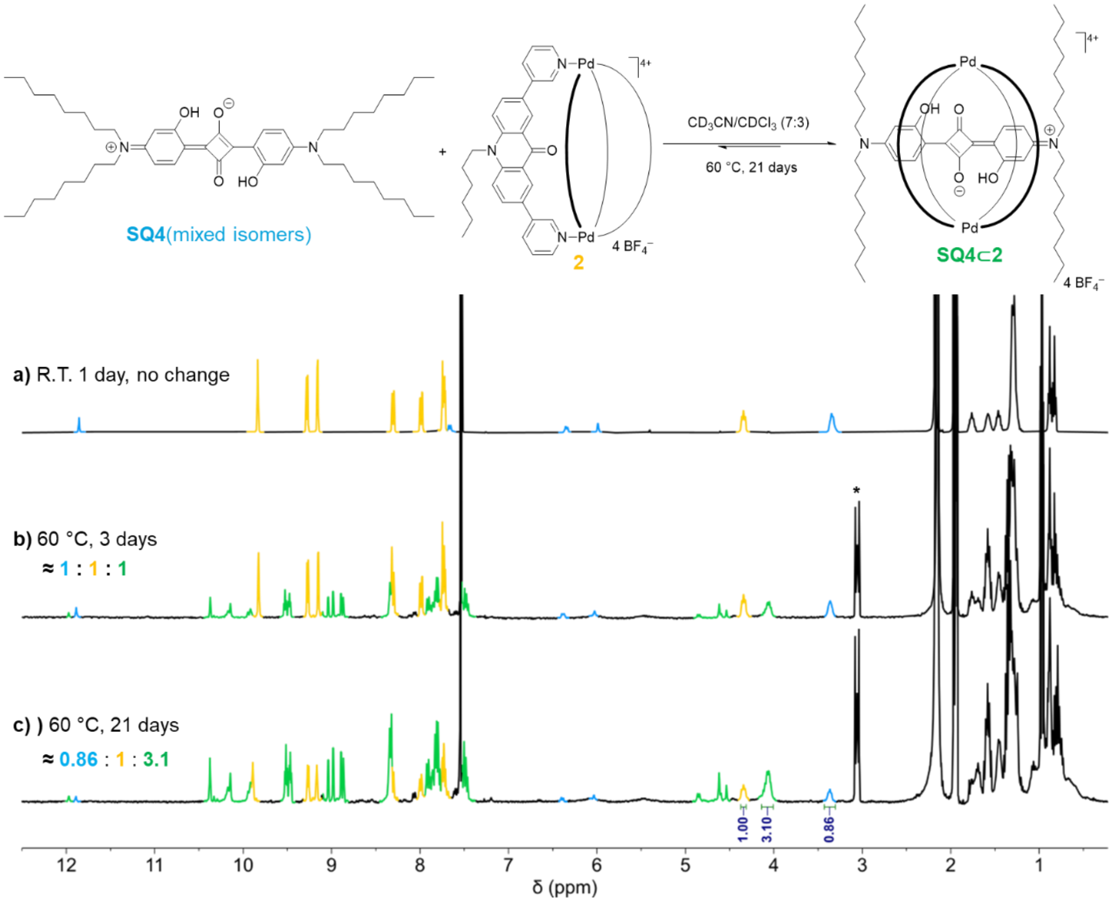

Figure S85. ^{1}H NMR spectra (400 MHz) of a mixture of **SQ4** (0.94 mM) and **2** (0.94 mM) in $CD_3CN/CDCl_3$ (7:3). (**a**) the spectrum remained unchanged after 1 day at R.T. (**b**) After 3 days at 60 °C, approx. 60% of the receptor **2** were bound. (**c**) After 21 days at 60 °C, equilibrium was reached. NBu_4BF_4 was added as a standard to cross-check concentrations (labeled with an asterisk). The association constant, $K_{a,\mathbf{SQ4}}$, defined as $K_{a,\mathbf{SQ4}} = \frac{[\mathbf{SQ4}\subset\mathbf{2}]}{[\mathbf{SQ4}]\times[\mathbf{2}]}$, was calculated to be 1.6×10^4 M^{-1}. The error is estimated to be 10%.

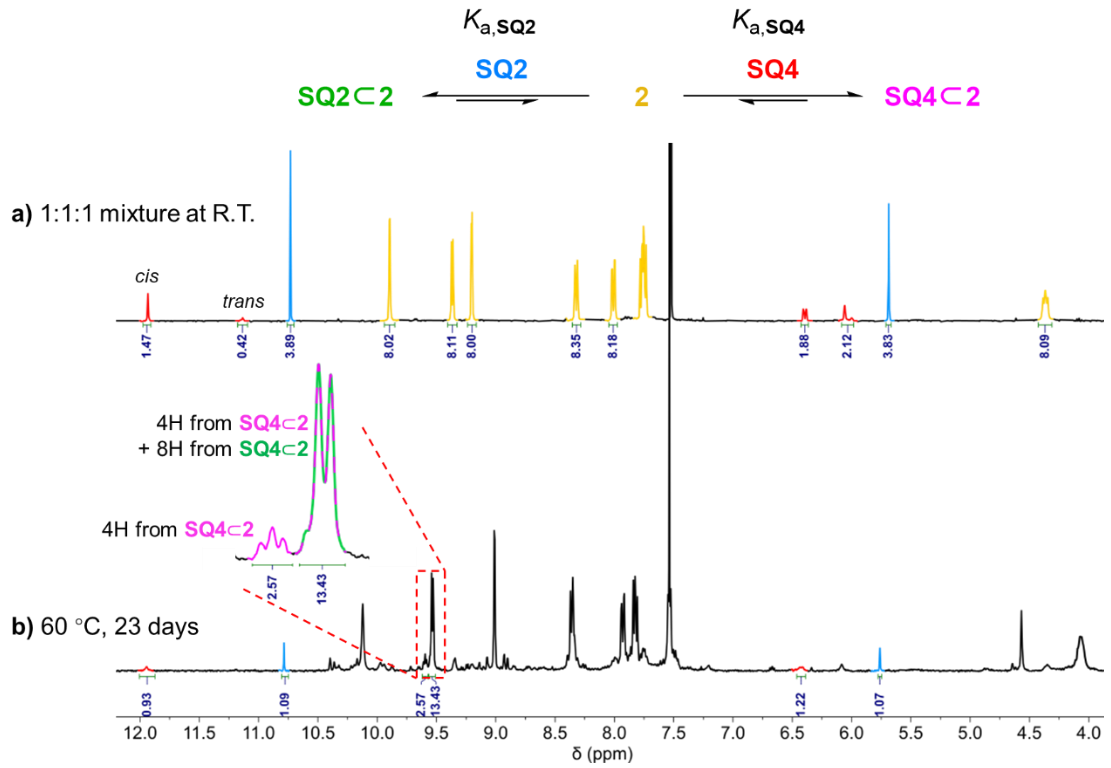

Figure S86. ^{1}H NMR spectra (400 MHz, 298 K) of a mixture of **SQ2**, **SQ4** and **2** (0.72 mM each) in $CD_3CN/CDCl_3$ (55:45). (**a**) Before heating and (**b**) after 23 days at 60 °C. From the phenyl protons of the dyes, the ratio [**SQ2**]/[**SQ4**] was calculated to be 1:2.26; from the outward-pointing pyridyl protons of host-guest complexes (insert), the ratio [**SQ2**⊂**2**]/[**SQ4**⊂**2**] was calculated to be 2.11:1. The relative association constant $K_{a,rel}$, defined as $K_{a,rel} = \frac{K_{a,\mathbf{SQ2}}}{K_{a,\mathbf{SQ4}}}$, is given by $K_{a,rel} = \frac{[\mathbf{SQ2}\subset\mathbf{2}]\times[\mathbf{SQ4}]}{[\mathbf{SQ4}\subset\mathbf{2}]\times[\mathbf{SQ2}]}$, and was calculated to be 4.8.

Therefore $K_{a,\mathbf{SQ2}} = K_{a,\mathbf{SQ2}} \times K_{a,rel} = 1.55 \times 10^4 \times 4.8 = 7.4 \times 10^4$ M^{-1}. Note that the association constants calculated by these NMR titrations correspond to equilibrium constants at 60 °C, and they are expected to differ from room temperature equilibrium constants.

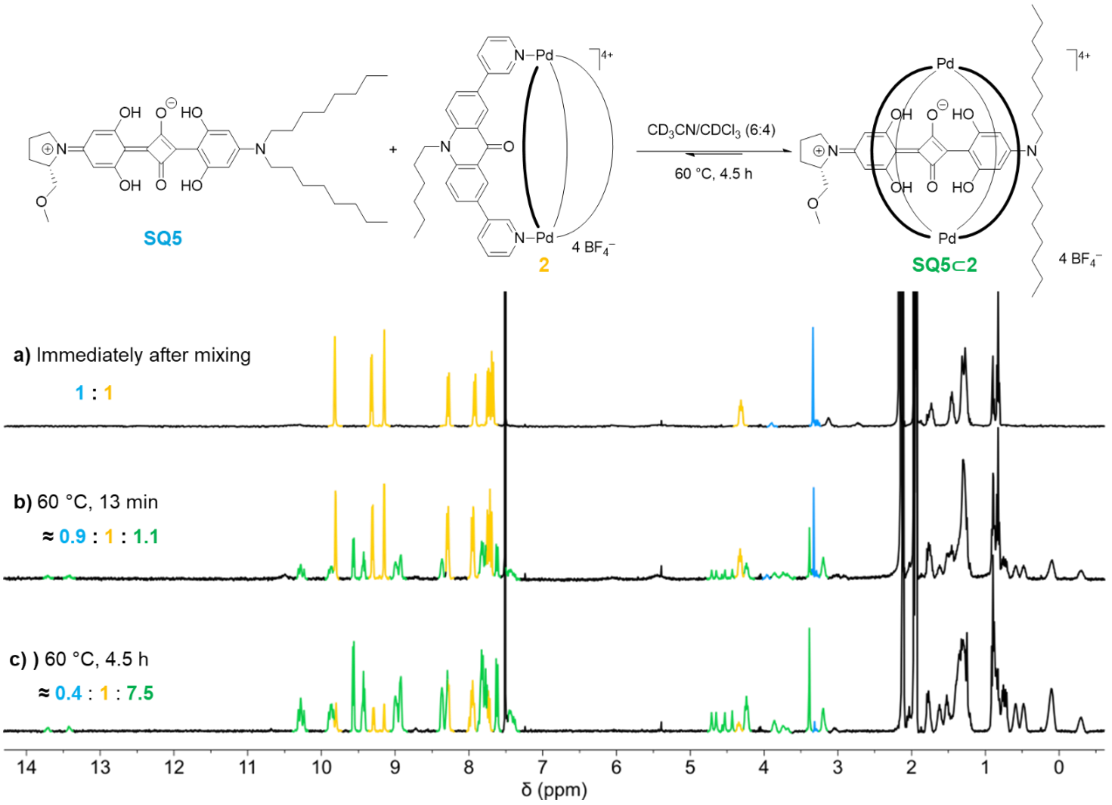

Figure S87. ^{1}H NMR spectra (400 MHz) of a mixture of **SQ5** (0.86 mM) and **2** (0.86 mM) in $CD_3CN/CDCl_3$ (6:4) (**a**) Immediately after mixing. (**b**) After heating to 60 °C for 13 min, approx. 53% of the receptor **2** were bound. (**c**) After heating for 4.5 h, equilibrium was reached.

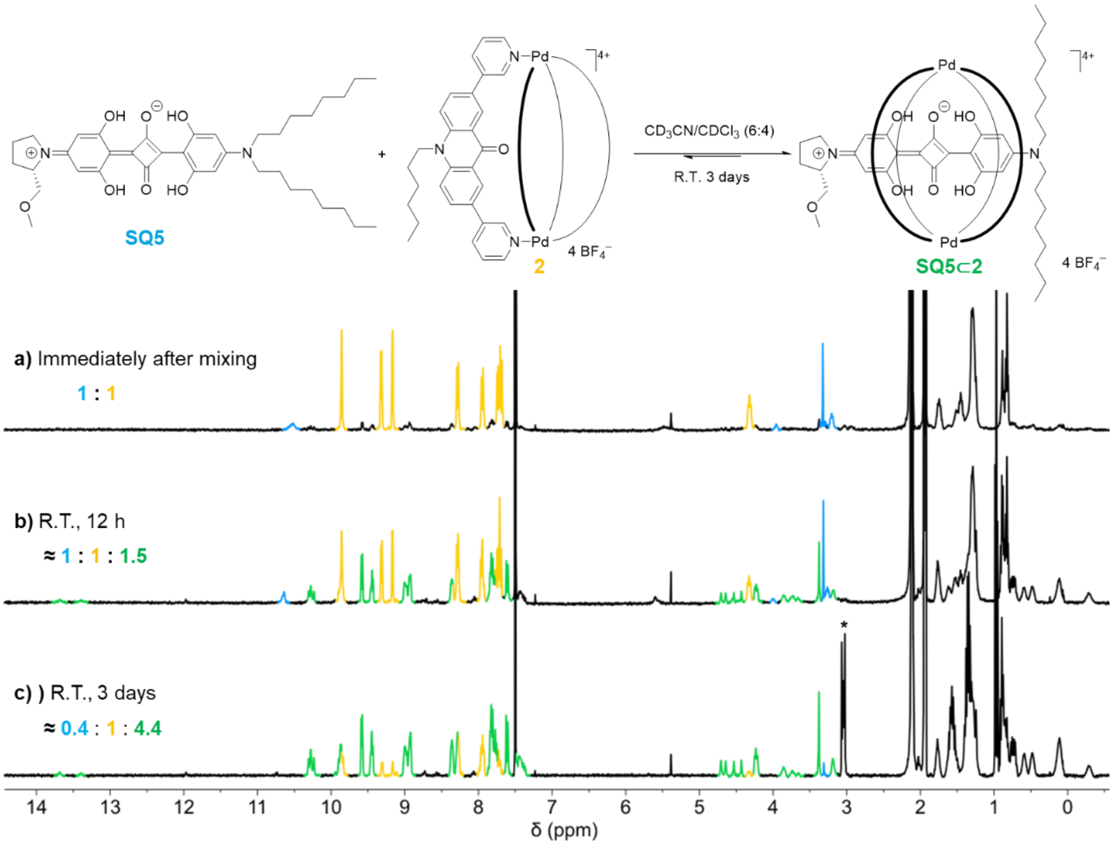

Figure S88. ^{1}H NMR spectra (400 MHz) of a mixture of **SQ5** (0.6 mM) and **2** (0.6 mM) in $CD_3CN/CDCl_3$ (6:4). (**a**) Immediately after mixing. (**b**) After 12 h at room temperature, approx. 60% of the receptor **2** were bound. (**c**) After 3 days at room temperature, equilibrium was reached.

3.2. UV-vis absorption titration of SQ1 + 2.

Common methods for UV-vis titration, involving adding increasing amounts of titrant into an analyte solution, could not be employed because of the slow equilibration (See Figures S100–S102). Instead, several samples were prepared, containing the same concentration of **SQ1** and different concentrations of **2**, in CH_3CN/CH_2Cl_2 (9:1). The solutions were heated to 60 °C for 2 h to accelerate the binding, and then allowed to equilibrate at R.T. for 12 h before measurement.

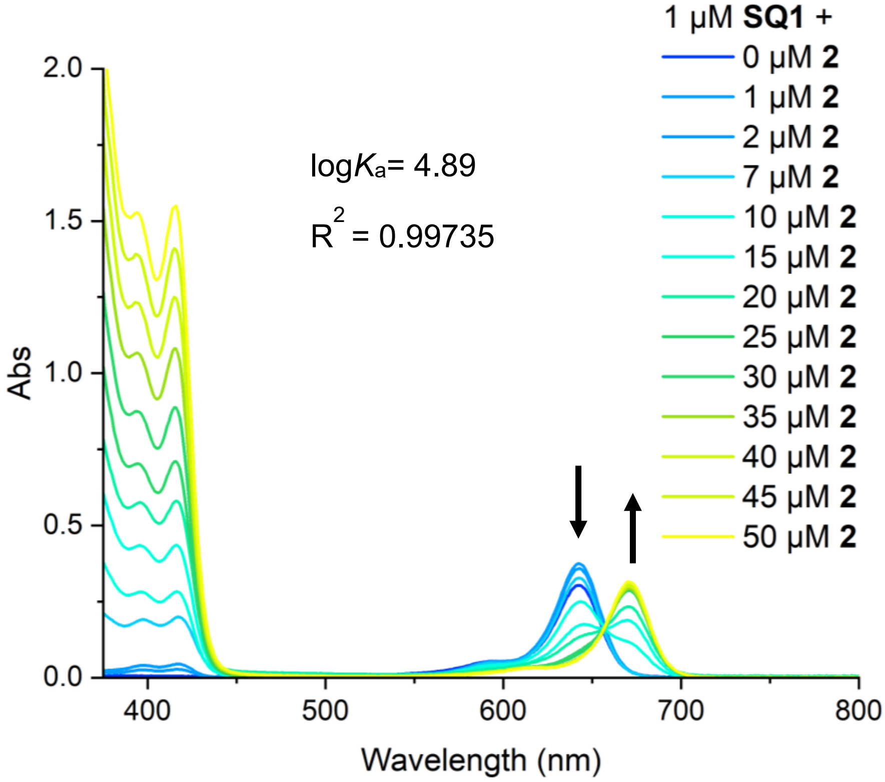

Figure S89. UV-vis absorption spectra of solutions containing 1.0 μM **SQ1** and 0–50 μM **2** (CH_3CN/CH_2Cl_2, 9:1).

The association constant was obtained by fitting the measured absorption data with a 1:1 binding model using SIVVU.org,[8] and the association constant $K_{a,\mathbf{SQ1}}$ was calculated to be 7.8×10^4 M^{-1}. The experimental error is estimated to be 10%.

This value is close to the $K_{a,\mathbf{SQ2}}$ = 7.4×10^4 M^{-1} calculated by NMR competition experiment (Figure S86). Therefore, it is justified to assume that the affinity of **2** for these squaraine dyes is mainly determined by the core of the dyes, rather than the substituents on the nitrogen atoms, and that the tetrahydroxy squaraine dyes (**SQ1**, **SQ2** and **SQ5**) likely have similar affinities for **2**.

4. Photophysical properties

4.1. UV-vis absorption and fluorescence

For the ligand **1** and squaraine dyes **SQ1**, **SQ2** and **SQ5**, fluorescence measurements were performed at a concentration ensuring Absorbance < 0.1 at the excitation or emission wavelengths to avoid inner-filter effect. For cage-containing samples, namely **2**, **SQ1⊂2**, **SQ2⊂2** and **SQ5⊂2**, the concentration was kept at 1.0 µM or above in order to minimize cage dissociation, and were measured immediately after dilution unless stated otherwise.

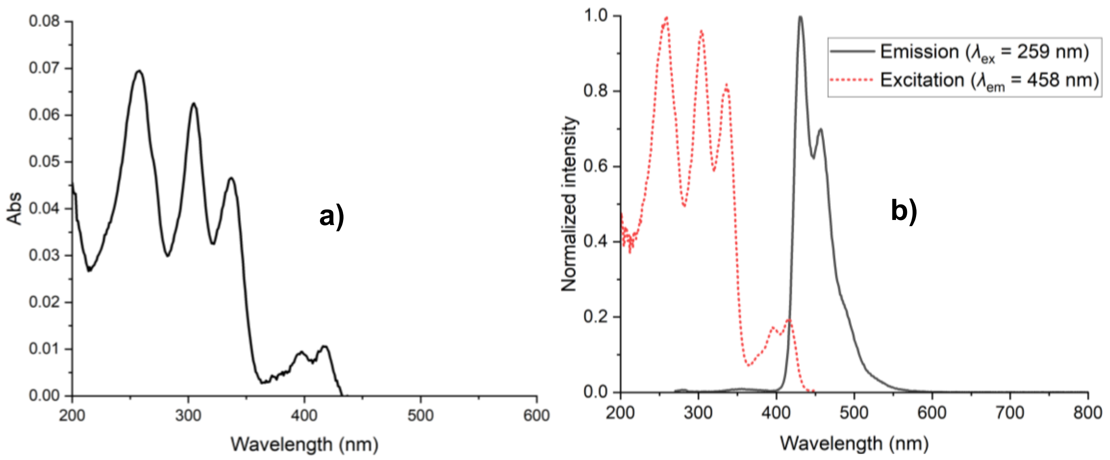

Figure S90. (**a**) UV-vis absorption spectrum and (**b**) normalized excitation and emission spectra of **1** (2.0 µM, CH_3CN).

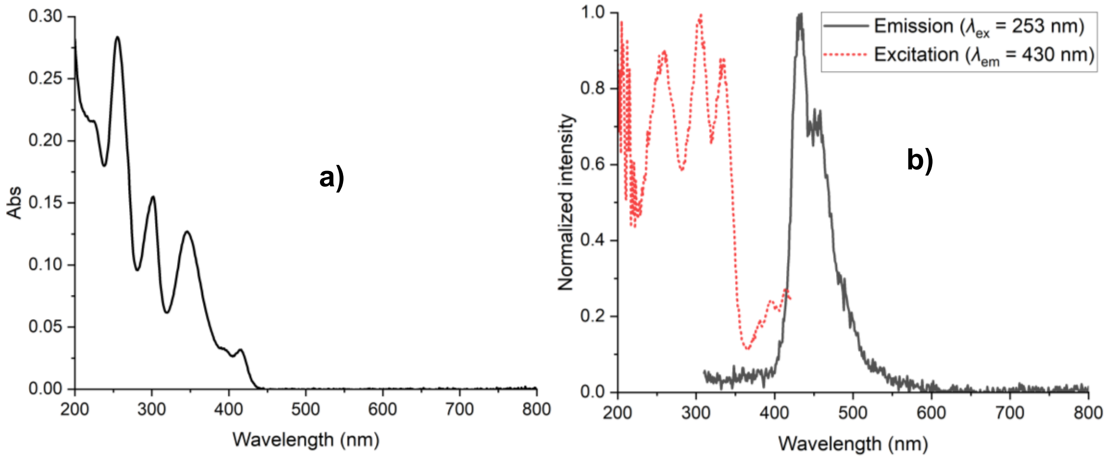

Figure S91. (**a**) UV-vis absorption spectrum and (**b**) normalized excitation and emission spectra of **2** (1.0 µM, CH_3CN), measured immediately after dilution. The fluorescence spectra are noisy because the intensity is very low (see comparison below).

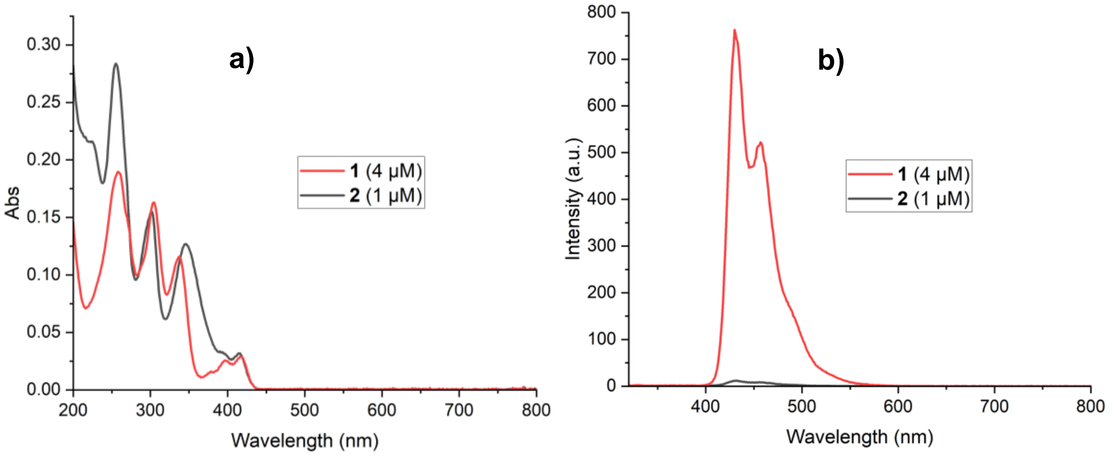

Figure S92. Overlay of the (**a**) UV-vis absorption spectra and (**b**) fluorescence emission spectra (λ_{ex} = 300 nm) of **1** (4.0 μM, CH_3CN) and **2** (1.0 μM, CH_3CN) measured under the same conditions for comparison. The concentrations of **1** and **2** are in 4:1 ratio to ensure the same amount of '**1**' in solution, since each cage **2** contains four ligands **1**. At this concentration, Abs(300 nm) > 0.1, therefore the emission intensities are not quantitative, but allow a qualitative comparison.

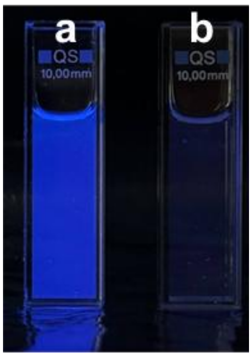

Figure S93. Pictures under 352 nm UV light of **1** (8.0 μM, CH_3CN) (**a**) and **2** (2.0 μM, CH_3CN) (**b**).

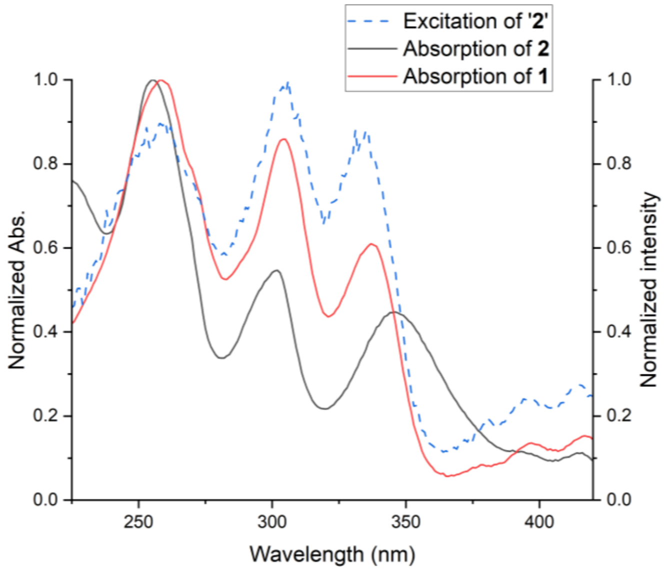

Figure S94. Overlay of the normalized UV-vis absorption spectra of **1** (4.0 μM, CH_3CN) and **2** (1.0 μM, CH_3CN) and the fluorescence excitation spectrum (λ_{em} = 430 nm) of **2**. One can observe that the shape of the excitation spectrum of cage **2** differs from its absorption spectrum, but resembles the absorption spectrum of ligand **1**. Therefore, we hypothesize that the observed emission of **2** mostly comes from a part of **2** that dissociated and released the strongly fluorescent ligand **1**. The true fluorescence of cage **2** should be lower than what we measured, meaning that the quenching upon cage formation is very efficient.

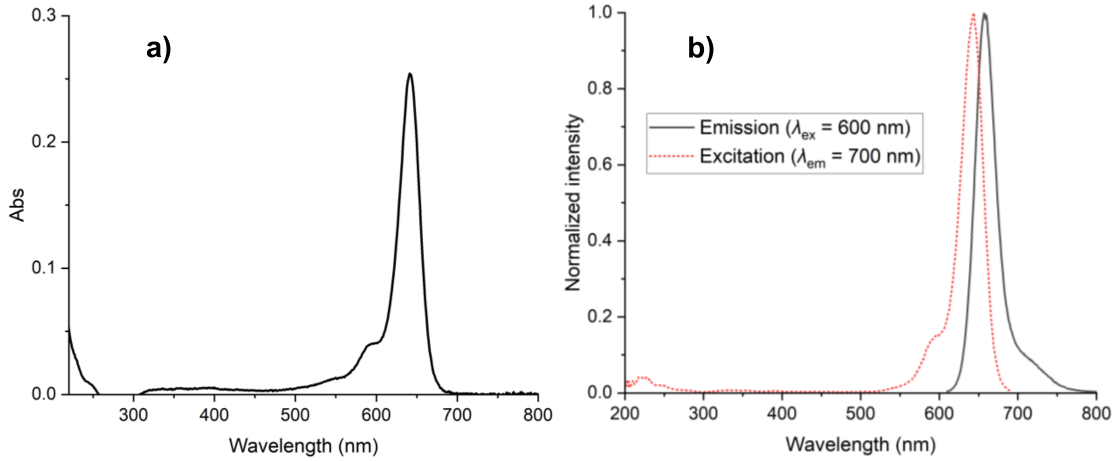

Figure S95. (**a**) UV-vis absorption spectrum and (**b**) normalized excitation and emission spectra of **SQ1** (1.0 μM, CH_3CN).

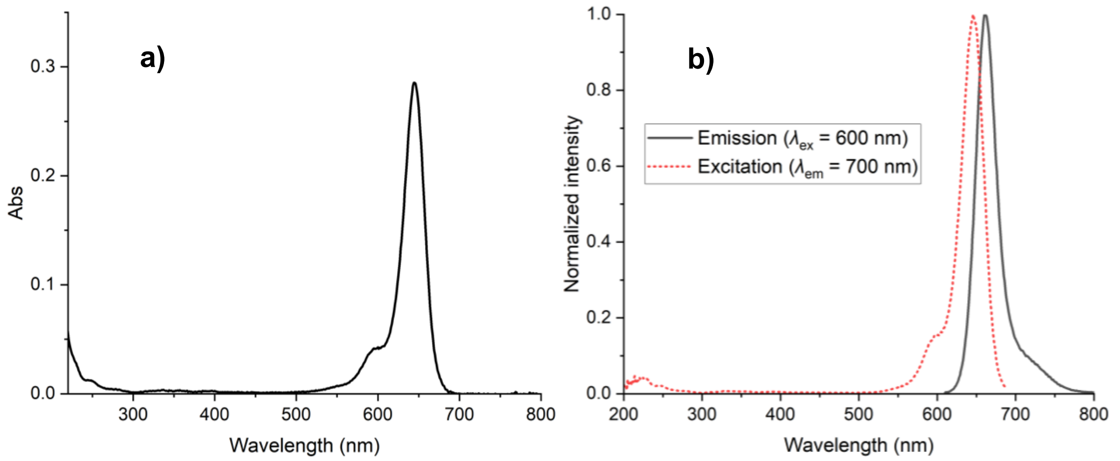

Figure S96. (**a**) UV-vis absorption spectrum and (**b**) normalized excitation and emission spectra of **SQ2** (1.0 μM, CH_3CN).

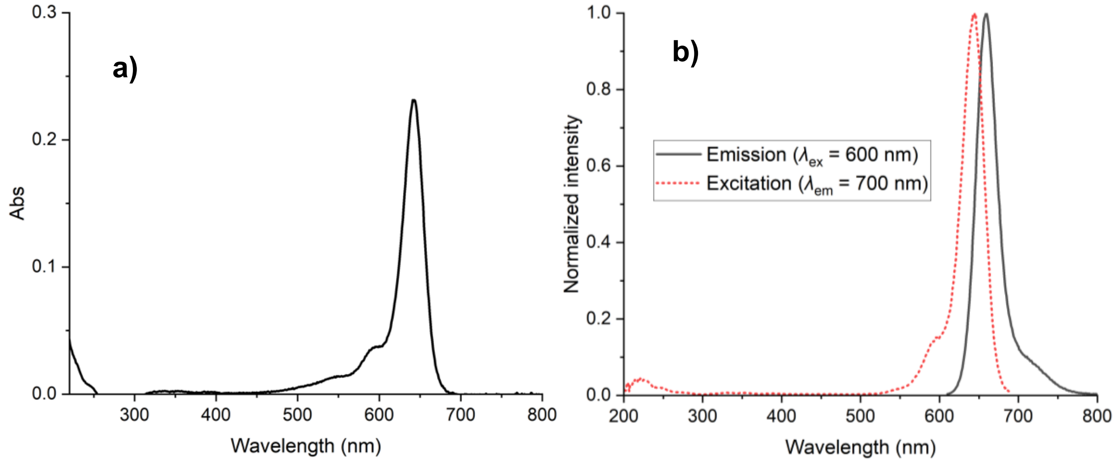

Figure S97. (**a**) UV-vis absorption spectrum and (**b**) normalized excitation and emission spectra of **SQ5** (1.0 μM, CH_3CN).

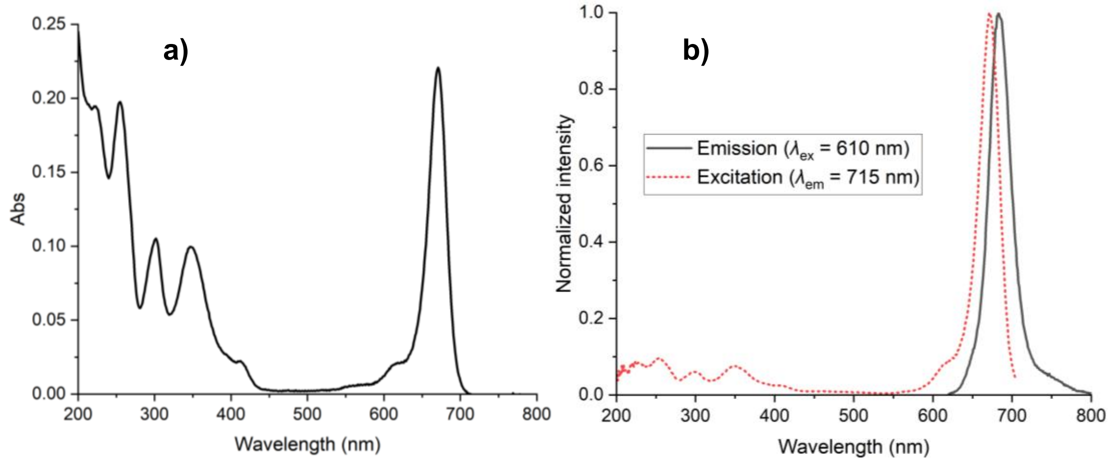

Figure S98. (**a**) UV-vis absorption spectrum and (**b**) normalized excitation and emission spectra of **SQ1⊂2** (1.0 μM, CH_3CN).

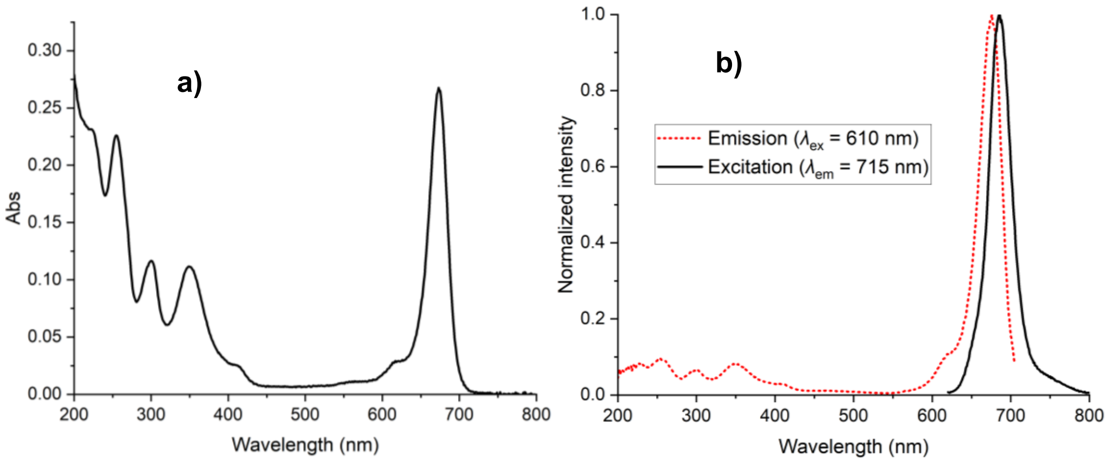

Figure S99. (**a**) UV-vis absorption spectrum and (**b**) normalized excitation and emission spectra of **SQ2⊂2** (1.0 μM, CH_3CN).

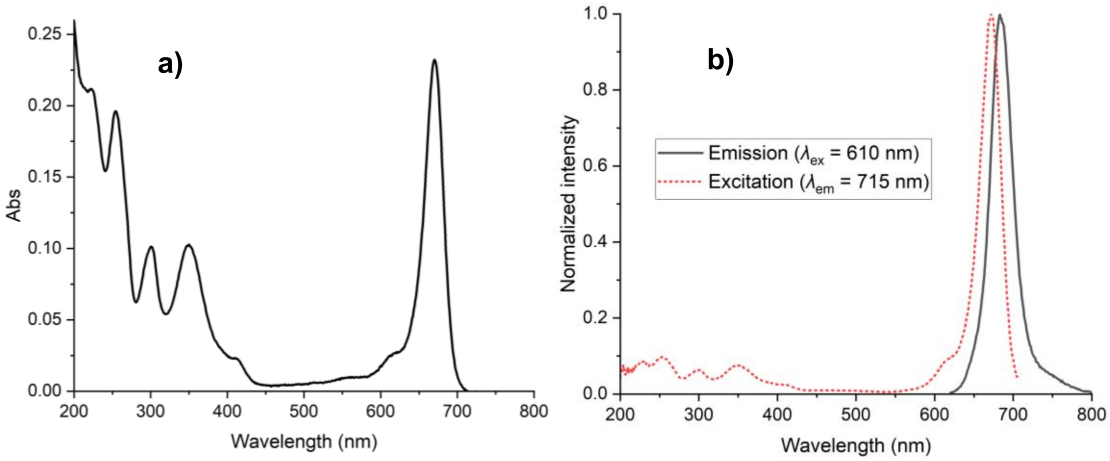

Figure S100. (**a**) UV-vis absorption spectrum and (**b**) normalized excitation and emission spectra of **SQ5⊂2** (1.0 μM, CH_3CN).

Table S1. Absorption, excitation, and emission maxima of the studied ligand, cage, dyes, and dye-in-cage adducts. The encapsulation of the squaraine dyes into **2** caused a red shift of 24–30 nm in all three maxima.

Compound	$\lambda_{abs, Max}$	$\lambda_{ex, Max}$	$\lambda_{em, Max}$
**1**	258	259	431
**2**	255	309*	433*
**SQ1**	641	644	657
**SQ1⊂2**	671	672	683
**SQ2**	645	646	661
**SQ2⊂2**	673	676	686
**SQ5**	641	645	659
**SQ5⊂2**	670	672	683

*As explained above, the observed fluorescence emission of **2** is most likely due to a small fraction of **2** dissociating upon dilution, releasing the emissive ligand **1**.

4.2. Unthreading kinetics monitored by absorption and fluorescence

After dilution of **SQ1⊂2**, **SQ2⊂2** and **SQ5⊂2** from millimolar concentrations to 1.0 μM, the time it takes for re-equilibration (by unthreading of the dyes) was monitored by UV-vis absorption and fluorescence emission spectroscopy. The cuvettes were sealed with parafilm to minimize evaporation.

In the absorption spectra, the release of free squaraine dyes is characterized by a decrease at 670 nm and an increase at 645 nm. In the emission spectra (λ_{ex} = 610 nm), the release of free squaraine dyes manifests as a strong increase at 660 nm. The reason why the latter is more pronounced is because the 610 nm excitation wavelength can excite free squaraine dyes much more efficiently than the encapsulated squaraine dyes (see Figures S95–S100).

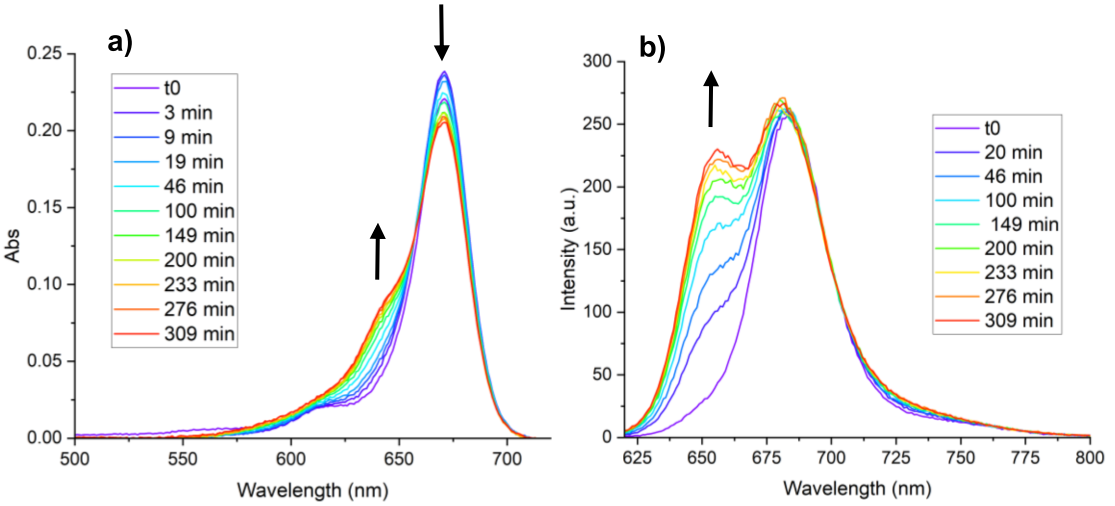

Figure S101. Evolution over time of (**a**) UV-vis absorption and (**b**) emission (λ_{ex} = 610 nm) of **SQ1⊂2** after dilution from 1.4 mM to 1.0 μM (CH_3CN).

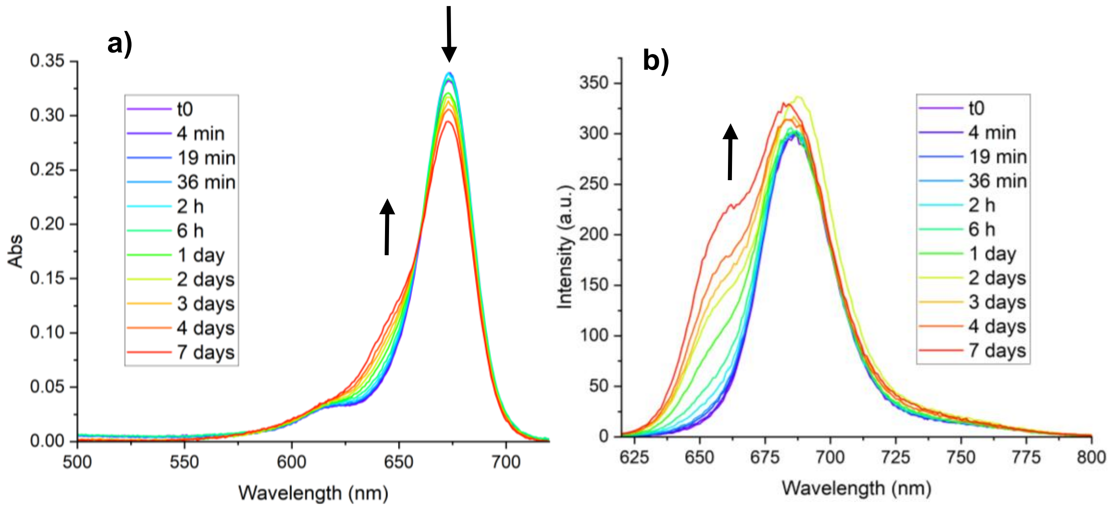

Figure S102. Evolution over time of (**a**) UV-vis absorption and (**b**) emission (λ_{ex} = 610 nm) of **SQ2⊂2** after dilution from 1.28 mM to 1.0 μM (CH_3CN).

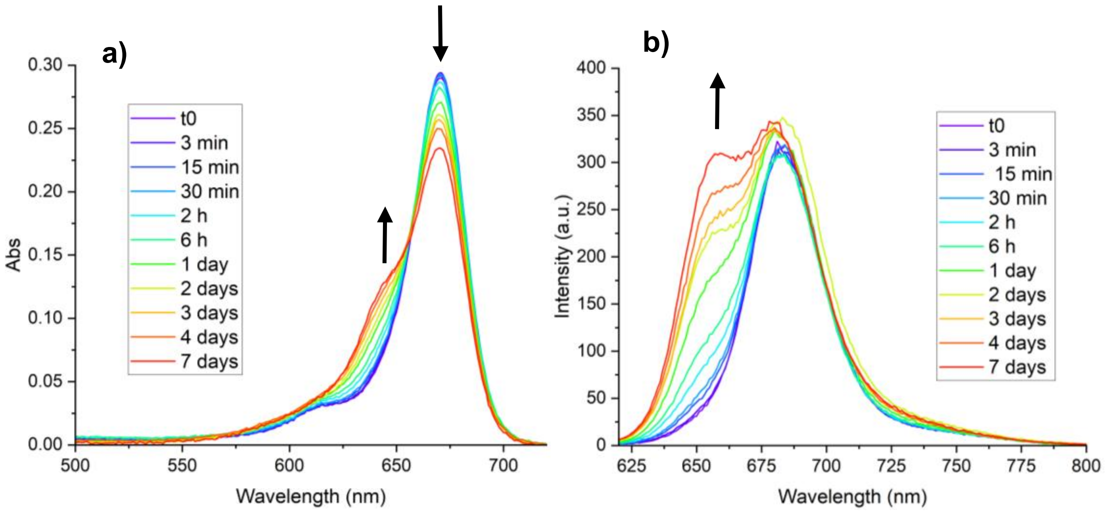

Figure S103. Evolution over time of (**a**) UV-vis absorption and (**b**) emission (λ_{ex} = 610 nm) of **SQ5⊂2** after dilution from 1.27 mM to 1.0 μM (CH_3CN).

By comparing these measurements, we can observe a trend in kinetic stability: **SQ1⊂2** < **SQ5⊂2** < **SQ2⊂2**. Therefore **SQ2⊂2** was used for the following studies to minimize dye release during the experiments.

4.3. Dark resonance energy transfer in SQ2⊂2

To confirm that encapsulation of the squaraine dyes into **2** was necessary for energy transfer, we studied the following two samples under the same conditions, so the intensities are directly comparable:

- '**SQ2** + **2**': 1.0 µM of **SQ2** and 1.0 µM of **2** in CH_3CN, obtained by diluting from a 0.3 mM CH_2Cl_2 stock solution of **SQ2** and a 2.6 mM CD_3CN stock solution of **2**. Final solvent composition > 99.6% CH_3CN.
- **SQ2⊂2**: 1.0 µM, CH_3CN, diluted from a 1.28 mM CD_3CN stock solution.

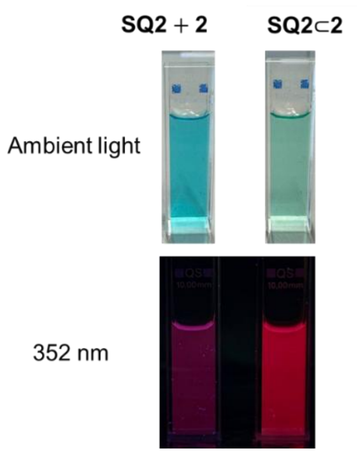

Figure S104. Pictures under ambient light (**top**) and 352 nm UV light (**bottom**) of **SQ2** + **2** (2.8 µM each, CH_3CN) (**left**) and **SQ2⊂2** (2.8 µM, CH_3CN) (**right**).

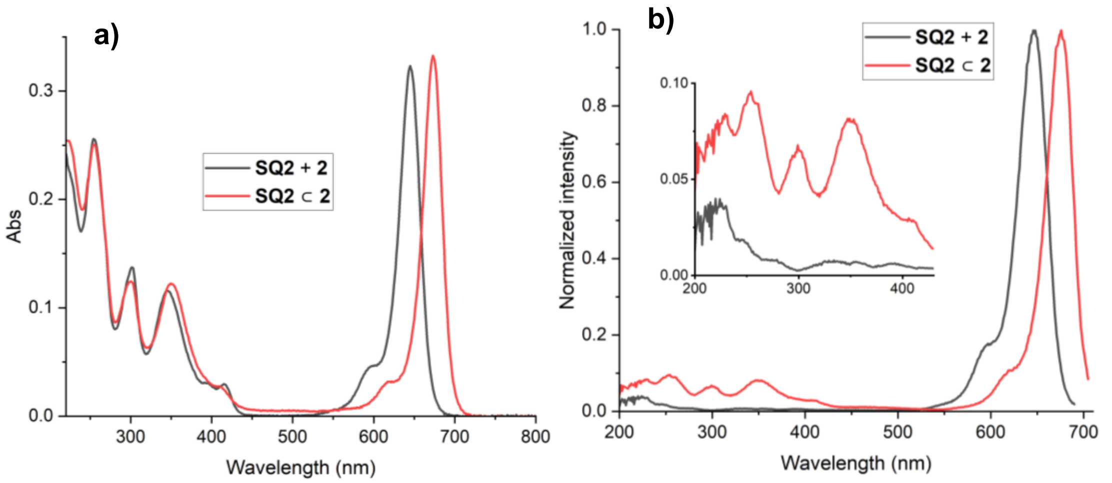

Figure S105. (**a**) UV-vis absorption spectra and (**b**) Normalized excitation spectra (λ_{em} = 700 nm) of **SQ2** + **2** (1.0 µM each, CH_3CN) (**black**) and **SQ2⊂2** (1.0 µM, CH_3CN) (**red**). Insert: zoom of the 200–420 nm region. Upon encapsulation, the squaraine dye showed three new excitation bands at 253 nm, 300 nm, and 350 nm, coinciding with the absorption maxima of the cage (See the main text, Figure 4c), while the dye outside the cage could only be weakly excited in this region.

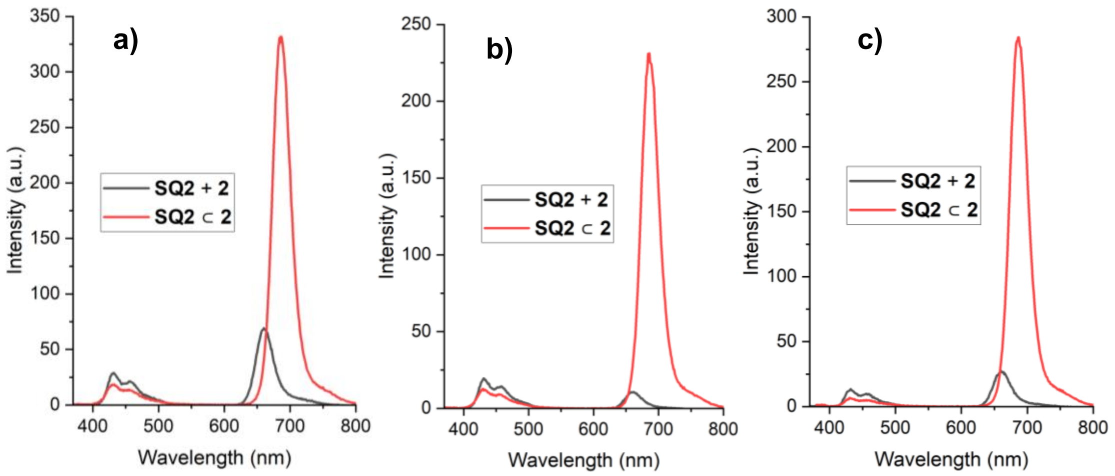

Figure S106. Fluorescence emission spectra of **SQ2 + 2** (1.0 µM each, CH_3CN) (**black**) and **SQ2⊂2** (1.0 µM, CH_3CN) (**red**), with excitation wavelengths at 253 nm (**a**), 300 nm (**b**) and 350 nm (**c**). When excited at the three aforementioned maxima, the encapsulated squaraine emits with increased intensity compared to the free squaraine, despite the latter being in the same solution as the cage. This shows that encapsulation into the cage is required for energy transfer to occur.

4.4. Photoluminescence quantum yield

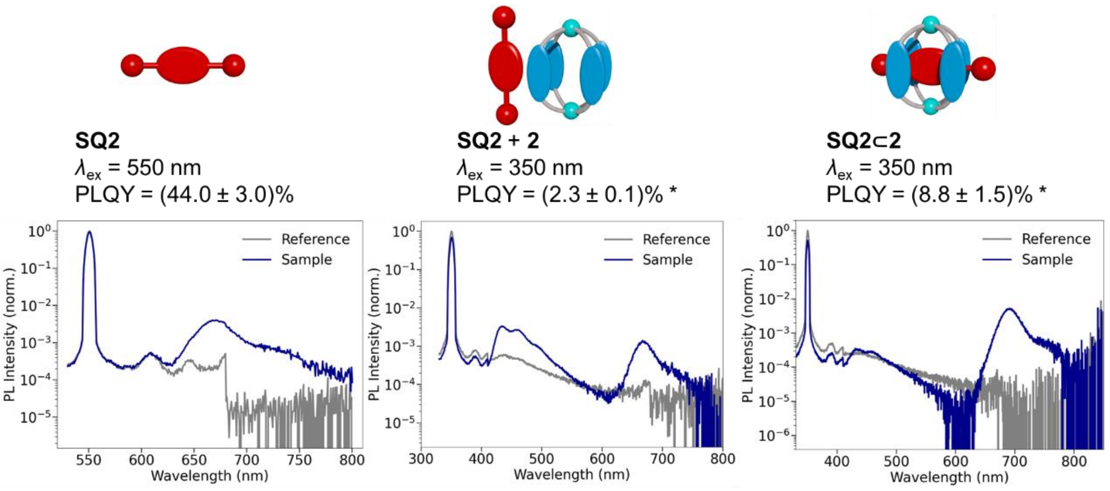

Figure S107. Photoluminescence spectra of (**a**) **SQ2** (2.0 µM, CH_3CN/CH_2Cl_2 (9:1), λ_{ex} = 550 nm), (**b**) **SQ2** + **2** (2.0 µM each, CH_3CN/CH_2Cl_2 (9:1), λ_{ex} = 350 nm), and (**c**) **SQ2⊂2** (2.0 µM, CH_3CN, λ_{ex} = 350 nm). *: Integrated only over the 600–850 nm region. Note that, as shown in Figure S94, the cage is partially degraded upon dilution, causing the ‘true’ concentration of the encapsulated dye to be lower. Therefore, the PLQY can only provide a qualitative comparison.

4.5. Circular dichroism

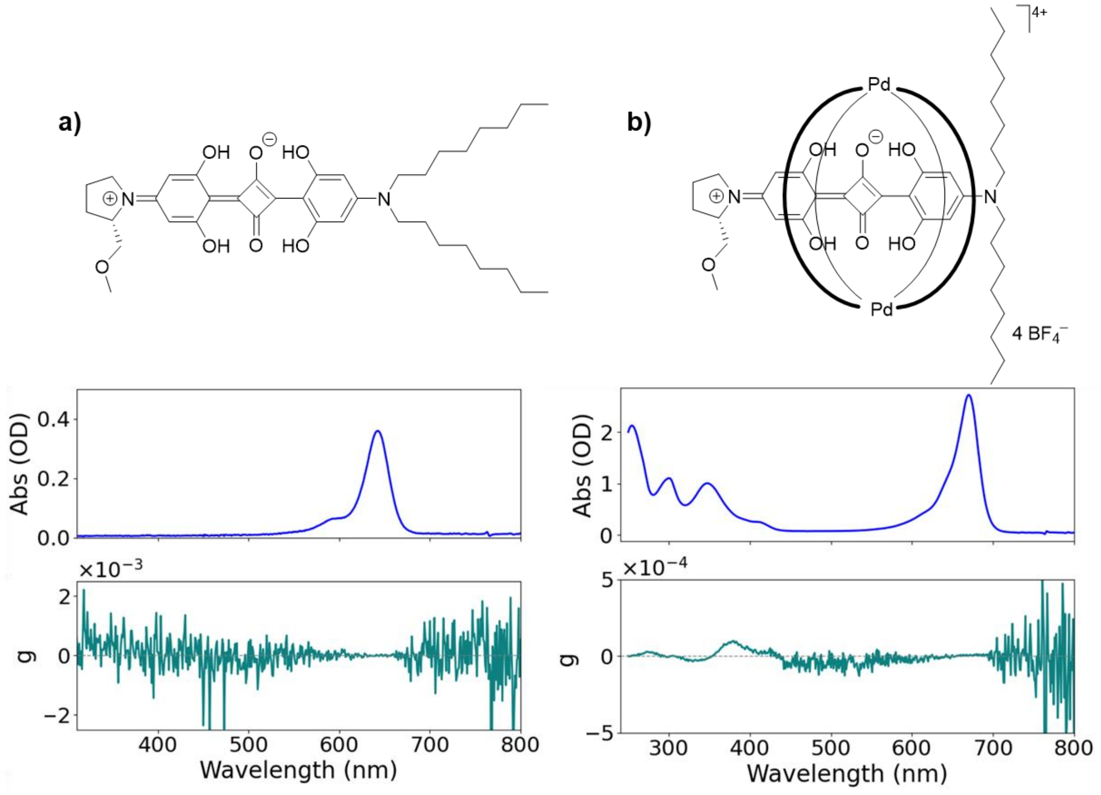

Figure S108. UV-vis absorption and CD spectra of **SQ5** (2 µM, CH_3CN/CH_2Cl_2 (1:1)) (**a**) and **SQ5⊂2** (10 µM, CH_3CN) (**b**).

4.6. Ultrafast transient absorption

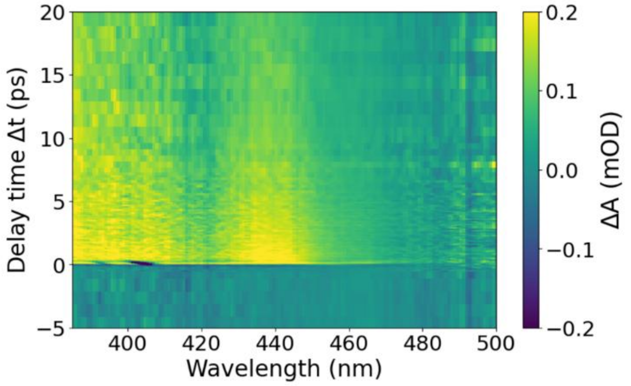

Figure S109. Transient absorption map of **2** (20 µM, CH_3CN) following 360 nm photoexcitation. The color scale represents the change in absorbance (ΔA). The ground state bleaching is not resolvable, possibly because it happens on sub-300 fs timescale.

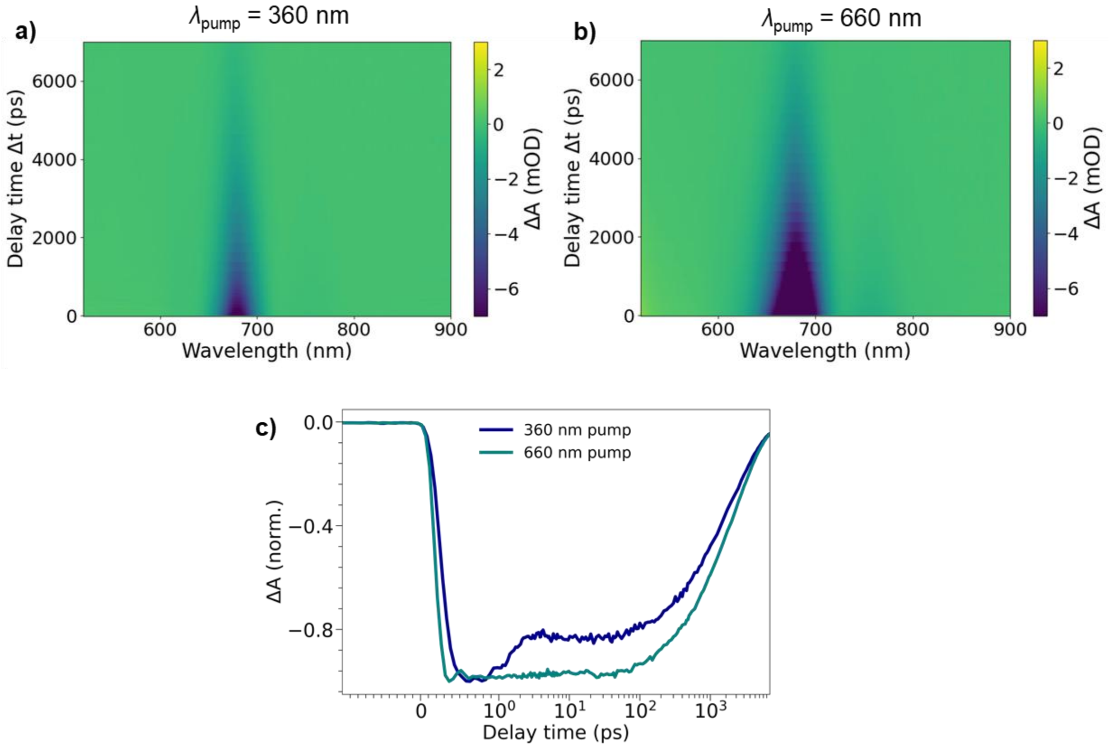

Figure S110. Transient absorption maps of **SQ2⊂2** (20 µM, CH_3CN) following 360 nm (**a**) or 660 nm (**b**) photoexcitation, and TA kinetic traces at a probe wavelength of 680 nm, following 360 nm (blue curve) or 660 nm (green curve) photoexcitation (**c**). A zoom of the < 10 ps region was shown in the main text, Figure 4d.

5. Crystallographic Data

5.1. $[Pd_2(\mathbf{1})_4](CF_3SO_3)_4$

In order to facilitate the growth of single crystals suitable for X-ray diffraction, a cage analogous to **2** was prepared using palladium triflate ($Pd(CF_3SO_3)_2$) as the palladium source.

Single crystals of $[Pd_2(\mathbf{1})_4](CF_3SO_3)_4$ were obtained by slow (several weeks) vapor diffusion of diethyl ether and ethyl acetate (vapor pressure 1:1) into a CD_3CN solution of $[Pd_2(\mathbf{1})_4](CF_3SO_3)_4$ (1.1 mM).

A clear light-yellow prism-shaped crystal with dimensions 0.16 × 0.12 × 0.06 mm was mounted. Data were collected using a XtaLAB Synergy R, DW system, HyPix-Arc 150 diffractometer operating at T = 139.99(10) K.

Data were measured using *w* scans with Cu*Kα* radiation. The diffraction pattern was indexed and the total number of runs and images was based on the strategy calculation from the program CrysAlis^{Pro} system (CCD 44.104a 64-bit (release 03-04-2025)).[9] The maximum resolution achieved was Θ = 75.615°(0.80 Å).

The unit cell was refined using CrysAlis^{Pro}[9] on 28535 reflections, 40% of the observed reflections.

Data reduction, scaling and absorption corrections were performed using CrysAlis^{Pro}.[9] The final completeness is 99.60 % out to 75.615° in Θ. A Gaussian absorption correction was performed using CrysAlis^{Pro} 1.171.44.103a.[9] Numerical absorption correction based on Gaussian integration over a multifaceted crystal model. Empirical absorption correction using spherical harmonics as implemented in SCALE3 ABSPACK scaling algorithm. The absorption coefficient μ of this material is 3.668 mm^{-1} at this wavelength (λ = 1.54184Å) and the minimum and maximum transmissions are 0.651 and 1.000.

The structure was solved in the space group $P\bar{1}$ (# 2) by ShelXT 2018/2 [10] using dual methods. It was refined by full matrix least squares minimization on $|F|^2$ using version 2019/3 of ShelXL.[11] All non-hydrogen atoms were refined anisotropically.

Hydrogen atom positions were calculated geometrically and refined using the riding model.

A solvent mask was calculated and 57 electrons were found in a volume of 177 Å^3 in 2 voids per unit cell. This is consistent with the presence of 2.5[CH_3CN] per Asymmetric Unit which account for 55 electrons per unit cell.

The value of Z' is 0.5.[12] This means that only half of the formula unit is present in the asymmetric unit, with the other half consisting of symmetry equivalent atoms. The moiety formula is $C_{116}H_{108}N_{12}O_4Pd_2$, 4($CF_3O_3S$), 4($C_2H_3N$), 2.5[$C_2H_3N$].

Table S2. Crystal data and structure refinement for $[Pd_2(\mathbf{1})_4](CF_3SO_3)_4$.

**Compound**	$[Pd_2(\mathbf{1})_4](CF_3SO_3)_4$
Formula	$C_{133}H_{127.5}F_{12}N_{18.5}O_{16}Pd_2S_4$
$D_{calc.}$/ g cm^{-3}	1.471
μ/mm^{-1}	3.668
Formula Weight	2810.07
Color	clear light yellow
Shape	prism-shaped
Size/mm^3	0.16×0.12×0.06
T/K	139.99(10)
Crystal System	triclinic
Space Group	$P\bar{1}$
a/Å	12.40356(15)
b/Å	16.2836(2)
c/Å	17.8075(2)
α/°	67.9425(13)
β/°	88.3685(10)
γ/°	72.8980(12)
V/Å^3	3172.36(8)
Z	1
Z'	0.5
Wavelength/Å	1.54184
Radiation type	Cu$K\alpha$
Θ_{min}/°	2.689
Θ_{max}/°	75.615
Measured Refl's.	71056
Indep't Refl's	12840
Refl's I≥2σ(I)	11926
R_{int}	0.0201
Parameters	879
Restraints	280
Largest Peak/e Å^{-3}	1.249
Deepest Hole/e Å^{-3}	–0.949
GooF	1.048
wR_2 (all data)	0.1414
wR_2	0.1393
R_1 (all data)	0.0547
R_1	0.0519
CCDC number	2491403

5.2. $[Pd_2(1)_4(SQ5)](BF_4)_4$

Single crystals of $[Pd_2(\mathbf{1})_4(\mathbf{SQ5})](BF_4)_4$ were obtained by slow (several weeks) vapor diffusion of diethyl ether and ethyl acetate (vapor pressure 1:1) into a CD_3CN solution of $[Pd_2(\mathbf{1})_4(\mathbf{SQ5})](BF_4)_4$ (1.3 mM).

A metallic dark green block-shaped crystal with dimensions 0.42 × 0.14 × 0.06 mm was mounted. Data were collected using a XtaLAB Synergy R, DW system, HyPix-Arc 150 diffractometer operating at T = 100.00(10) K.

Data were measured using *w* scans with Cu*Kα* radiation. The diffraction pattern was indexed and the total number of runs and images was based on the strategy calculation from the program CrysAlis^{Pro} system (CCD 44.113a 64-bit (release 02-06-2025)).[9]The maximum resolution achieved was Θ = 75.125°.

The unit cell was refined using CrysAlis^{Pro}[9] on 44388 reflections, 26% of the observed reflections.

Data reduction, scaling and absorption corrections were performed using CrysAlis^{Pro}.[9] The final completeness is 100.00 % out to 75.125° in Θ. A Gaussian absorption correction was performed using CrysAlis^{Pro} 1.171.44.115a.[9] Numerical absorption correction based on Gaussian integration over a multifaceted crystal model. Empirical absorption correction using spherical harmonics as implemented in SCALE3 ABSPACK scaling algorithm. The absorption coefficient μ of this material is 2.482 mm^{-1} at this wavelength (λ = 1.54184Å) and the minimum and maximum transmissions are 0.335 and 1.000.

The structure was solved in the space group $C222_1$ (# 20) by ShelXT 2018/2[10] using dual methods. It was refined by full matrix least squares minimization on $|F|^2$ using version 2019/3 of ShelXL 2019/3.[11] All non-hydrogen atoms were refined anisotropically. Hydrogen atom positions were calculated geometrically and refined using the riding model.

The model is refined as a 2-component inversion twin.
In the crystal structure, atom C29B lies on a special position corresponding to a crystallographic twofold rotation axis parallel to the *b*-axis. It is shared between two distinct orientations of the alkyl chains and is modelled with full occupancy. Due to this positional disorder and the symmetry constraints imposed by the special position, the hydrogen atoms of the methyl group could not be reliably located.

A solvent mask was calculated, and 1724 electrons were found in a volume of 6600Å^3 in 1 void per unit cell. This is consistent with the presence of 5 solvent molecules of diethyl ether per Asymmetric Unit which account for 1680 electrons per unit cell.

There is a single formula unit in the asymmetric unit, which is represented by the reported sum formula. In other words: Z is 8 and Z' is 1. The moiety formula is $C_{116}H_{106}N_{12}O_4Pd_2$, 4($BF_4$), $C_{38}H_{54}N_2O_7$, 5[$C_4H_{10}O$].[12]

The Flack parameter was refined to 0.109(12). Determination of absolute structure using Bayesian statistics on Bijvoet differences using the Olex2 results in 0.0246(9). The chiral atoms in this structure are: Pd1(R), Pd2(S), C26B(R), C124(S), C126(R), C136(S). Note: The Flack parameter is used to determine chirality of the crystal studied, the value should be near 0, a value of 1 means that the stereochemistry is wrong and the model should be inverted. A value of 0.5 means that the crystal consists of a racemic mixture of the two enantiomers.

Table S3. Crystal data and structure refinement for $[Pd_2(\mathbf{1})_4(\mathbf{SQ5})](BF_4)_4$.

**Compound**	$[Pd_2(\mathbf{1})_4(\mathbf{SQ5})](BF_4)_4$
Formula	$C_{174.02}H_{210.56}B_4F_{16}N_{14}O_{16}Pd_2$
$D_{calc.}$/ g cm^{-3}	1.344
μ/mm^{-1}	2.482
Formula Weight	3314.38
Color	metallic dark green
Shape	block-shaped
Size/mm^3	0.42×0.14×0.06
T/K	100.00(10)
Crystal System	orthorhombic
Flack Parameter	0.109(12)
Space Group	$C222_1$
a/Å	23.5236(4)
b/Å	39.5485(3)
c/Å	35.2072(8)
α/°	90
β/°	90
γ/°	90
V/Å^3	32754.0(10)
Z	8
Z'	1
Wavelength/Å	1.54184
Radiation type	Cu$K\alpha$
Θ_{min}/°	2.185
Θ_{max}/°	75.125
Index range h	–28 ≤ h ≤ 29
Index range k	–34 ≤ k ≤ 49
Index range l	–43 ≤ l ≤ 43
Measured Refl's.	168481
Indep't Refl's	32771
Refl's I≥2σ(I)	22452
R_{int}	0.0303
Parameters	1908
Restraints	3274
Largest Peak/e Å^{-3}	0.948
Deepest Hole/e Å^{-3}	–1.025
GooF	1.123
R_1 (I≥2*s*(I) / all)	0.0916 / 0.1128
wR_2 (I≥2*s*(I) / all)	0.2688 / 0.2924
CCDC number	2491404

6. Computational details

Geometry optimization was performed only on the cationic part of the host guest complexes (i.e., BF_4^- anions were not taken into account). Initial guesses for the structures of **SQ1⊂2**, **SQ2⊂2**, **SQ3⊂2**, **SQ4**(*cis*)**⊂2**, and **SQ4**(*trans*)**⊂2** were constructed in software Avogadro 1.2.0,[13] by positioning the host and guest molecules similarly to the binding mode observed in the crystal structure of **SQ5⊂2**. For **SQ1⊂2**, two binding modes were found to be close in energy, with either the NEt_2 side or the $NHex_2$ side closer to the center of the cage. The NMR analysis in section 2.2 helped us to determine that the latter should be the most favored.

The geometries were optimized first with the UFF force field in Avogadro, then with the GFN2-xTB[14] method implemented in the software xTB 6.6.1,[14] in implicit acetonitrile treated with the analytical linearized Poisson-Boltzmann solvent model (ALPB).[15]

A full description of the theory of NCI analysis can be found in the original works by Johnson et al.[16] and Contreras-García et al.[17]

To reduce computational costs, the NCI analysis was based on a model compound, $[Pd_2($**1***$)_4$**SQ***$]^{4+}$ (Scheme S11).

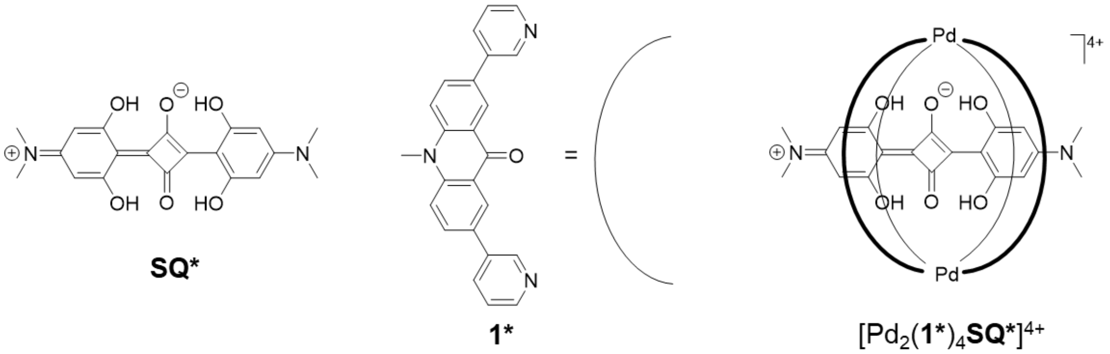

Scheme S11. Model compounds **SQ***, **1***, and $[Pd_2($**1***$)_4$**SQ***$]^{4+}$.

The initial geometry of $[Pd_2($**1***$)_4$**SQ***$]^{4+}$ was obtained by removing alkyl and (methoxymethyl)pyrrolidine substituents from the crystal structure of **SQ5⊂2**, leaving only N-methyl groups. This simplified structure was optimized at the GFN2-xTB level as mentioned above.

The electron density was obtained from a single-point calculation at the B3LYP/6-31+G(d,p),LanL2DZ level[18–22] in implicit acetonitrile treated by the polarizable continuum model (PCM)[23], using software Gaussian 16.[24] Software Multiwfn 3.8 [25,26] was used to convert the output file format from .FChk to .wfx for compatibility. The NCI analysis was performed with software NCIPLOT.[17] The s–sign(λ_2)ρ plots were generated by software gnuplot 6.0,[27] and the RDG isosurface was visualized in software VMD.[28]

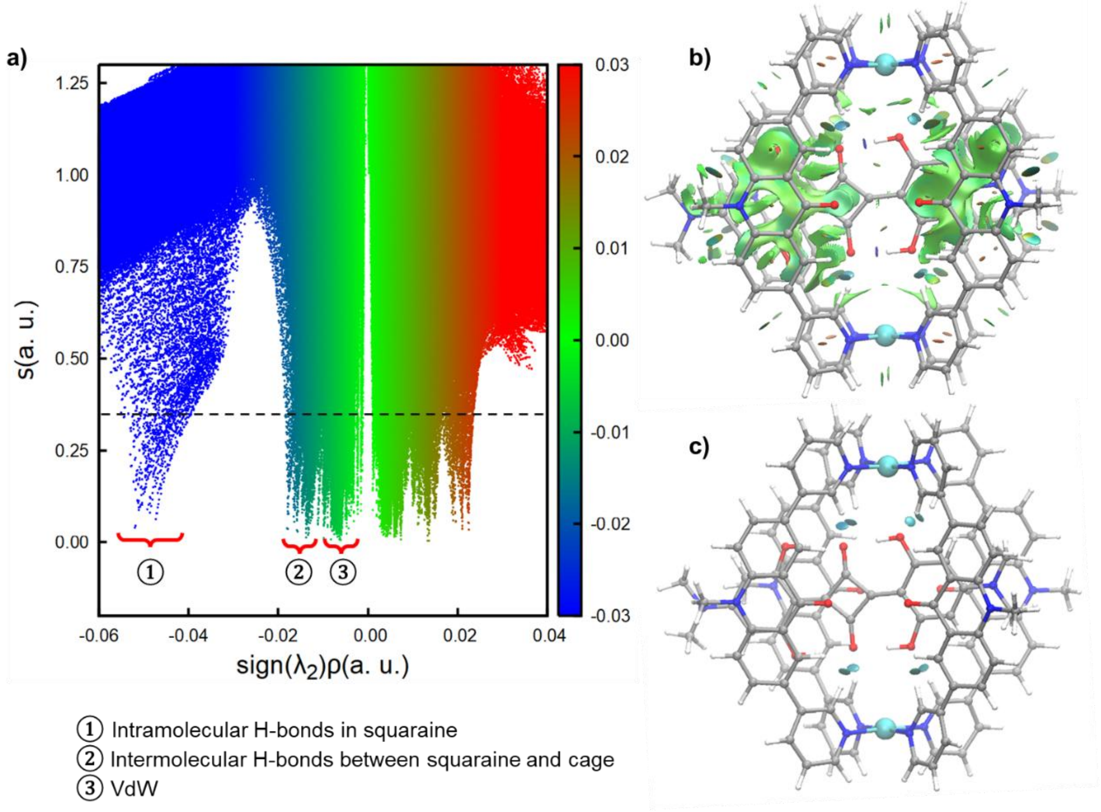

Figure S111. NCI analysis of the model compound [Pd_2(**1***)$_4$**SQ***]$^{4+}$. (a) Scatter plot of the RDG (*s*) as a function of sign(λ_2)ρ, with a blue-green-red colour scale of –0.03 a.u. < ρ < 0.03 a.u. The dashed line represents the s = 0.35 a.u. cut-off that was chosen for the visualization in real space. (b) RDG isosurface (s = 0.35 a.u.) of [Pd_2(**1***)$_4$**SQ***]$^{4+}$. (c) Selected parts of the RDG isosurface (s = 0.35 a.u.) corresponding to intermolecular H-bonds. The surface was calculated within 0.5 Å radius around chosen coordinates using the RADIUS keyword.

7. References

[1] S. Löffler, J. Lübben, L. Krause, D. Stalke, B. Dittrich, G. H. Clever, *J. Am. Chem. Soc.* **2015**, *137*, 1060–1063.

[2] Yoon Sung Cheol, Lee Chang Jin, Lee Jaemin, Eom Seung Hun, **2023**, US11767318B2.

[3] C. W. Dirk, W. C. Herndon, F. Cervantes-Lee, H. Selnau, S. Martinez, P. Kalamegham, A. Tan, G. Campos, M. Velez, J. Zyss, I. Ledoux, L.-T. Cheng, *J. Am. Chem. Soc.* **1995**, *117*, 2214–2225.

[4] D. Schindler, A. L. Meza-Chincha, M. Roth, F. Würthner, *Chem. Eur. J.* **2021**, *27*, 16938–16946.

[5] B. Rama Raju, S. Naik, P. J. G. Coutinho, M. S. T. Gonçalves, *Dyes and Pigments*, **2013**, *99*, 220–227.

[6] J. Wojtyk, A. Mckerrow, P. Kazmaier, E. Buncel, *Can. J. Chem.* **1999**, *77*, 903–912.

[7] P. M. Kazmaier, G. K. Hamer, R. A. Burt, *Can. J. Chem.* **1990**, *68*, 530–536.

[8] Vander Griend, D.A.; DeVries, M. J.; Greeley, M.; Kim, Y.; Wang, N.; Buist, D; Ulry, C. SIVVU, http://sivvu.org, **2021**.

[9] CrysAlisPro Software System, Rigaku Oxford Diffraction, **2025**.

[10] G. M. Sheldrick, *Acta Crystallogr. Sect. A*, **2015**, *71*, 3–8.

[11] G. M. Sheldrick, *Acta Crystallogr. Sect. C*, **2015**, *71*, 3–8.

[12] O. V Dolomanov, L. J. Bourhis, R. J. Gildea, J. A. K. Howard, H. Puschmann, *J. Appl. Cryst.* **2009**, *42*, 339–341.

[13] M. D. Hanwell, D. E. Curtis, D. C. Lonie, T. Vandermeersch, E. Zurek, G. R. Hutchison, *J. Cheminf.* **2012**, *4*, 17.

[14] C. Bannwarth, S. Ehlert, S. Grimme, *J. Chem. Theory Comput.* **2019**, *15*, 1652–1671.

[15] S. Ehlert, M. Stahn, S. Spicher, S. Grimme, *J. Chem. Theory Comput.* **2021**, *17*, 4250–4261.

[16] E. R. Johnson, S. Keinan, P. Mori-Sánchez, J. Contreras-García, A. J. Cohen, W. Yang, *J. Am. Chem. Soc.* **2010**, *132*, 6498–6506.

[17] J. Contreras-García, E. R. Johnson, S. Keinan, R. Chaudret, J. P. Piquemal, D. N. Beratan, W. Yang, *J. Chem. Theory Comput.* **2011**, *7*, 625–632.

[18] A. D. Becke, *J. Chem. Phys.* **1992**, *96*, 2155–2160.

[19] C. Lee, eitao Yang, R. G. Parr, *Phys. Rev. B,* **1988**, *37*, 785–789.

[20] P. J. Stephens, F. J. Devlin, C. F. Chabalowski, M. J. Frisch, *Phys. Chem.* **1994**, *98*, 11623–11627.

[21] W. J. Hehre, K. Ditchfield, J. A. Pople, *J. Chem. Phys.* **1972**, *56*, 2257–2261.

[22] P. J. Hay, W. R. Wadt, *J. Chem. Phys.* **1985**, *82*, 270–283.

[23] J. Tomasi, B. Mennucci, R. Cammi, *Chem. Rev.* **2005**, *105*, 2999–3093.

[24] Gaussian 16, Revision C.01, M. J. Frisch, G. W. Trucks, H. B. Schlegel, G. E. Scuseria, M. A. Robb, J. R. Cheeseman, G. Scalmani, V. Barone, G. A. Petersson, H. Nakatsuji, X. Li, M. Caricato, A. V. Marenich, J. Bloino, B. G. Janesko, R. Gomperts, B. Mennucci, H. P. Hratchian, J. V. Ortiz, A. F. Izmaylov,

J. L. Sonnenberg, D. Williams-Young, F. Ding, F. Lipparini, F. Egidi, J. Goings, B. Peng, A. Petrone, T. Henderson, D. Ranasinghe, V. G. Zakrzewski, J. Gao, N. Rega, G. Zheng, W. Liang, M. Hada, M. Ehara, K. Toyota, R. Fukuda, J. Hasegawa, M. Ishida, T. Nakajima, Y. Honda, O. Kitao, H. Nakai, T. Vreven, K. Throssell, J. A. Montgomery, Jr., J. E. Peralta, F. Ogliaro, M. J. Bearpark, J. J. Heyd, E. N. Brothers, K. N. Kudin, V. N. Staroverov, T. A. Keith, R. Kobayashi, J. Normand, K. Raghavachari, A. P. Rendell, J. C. Burant, S. S. Iyengar, J. Tomasi, M. Cossi, J. M. Millam, M. Klene, C. Adamo, R. Cammi, J. W. Ochterski, R. L. Martin, K. Morokuma, O. Farkas, J. B. Foresman, and D. J. Fox, Gaussian, Inc., Wallingford CT, **2016**.

[25] T. Lu, F. Chen, *J. Comput. Chem.* **2012**, *33*, 580–592.

[26] T. Lu, *J. Chem. Phys.* **2024**, *161*, DOI 10.1063/5.0216272.

[27] T. Williams, C. Kelley, E. A. Merritt, gnuplot, Version 6.0, **2024,** https://www.gnuplot.info

[28] W. Humphrey, A. Dalke, K. Schulten, *J. Mol. Graph.* **1996**, *14*, 33–38.
